# A Systematic Analysis of Host Factors Reveals a Med23-Interferon-λ Regulatory Axis against Herpes Simplex Virus Type 1 Replication

**DOI:** 10.1371/journal.ppat.1003514

**Published:** 2013-08-08

**Authors:** Samantha J. Griffiths, Manfred Koegl, Chris Boutell, Helen L. Zenner, Colin M. Crump, Francesca Pica, Orland Gonzalez, Caroline C. Friedel, Gerald Barry, Kim Martin, Marie H. Craigon, Rui Chen, Lakshmi N. Kaza, Even Fossum, John K. Fazakerley, Stacey Efstathiou, Antonio Volpi, Ralf Zimmer, Peter Ghazal, Jürgen Haas

**Affiliations:** 1 Division of Pathway Medicine, University of Edinburgh, Edinburgh, United Kingdom; 2 Preclinical Target Development and Genomics and Proteomics Core Facilities, German Cancer Research Center, Heidelberg, Germany; 3 MRC-University of Glasgow Centre for Virus Research, Glasgow, United Kingdom; 4 Division of Virology, Department of Pathology Cambridge University, Cambridge, United Kingdom; 5 University of Rome Tor Vergata, Rome, Italy; 6 Institute for Informatics, Ludwig-Maximilians Universität München, München, Germany; 7 The Roslin Institute and Royal (Dick) School of Veterinary Studies, University of Edinburgh, Edinburgh, United Kingdom; 8 Centre for Systems Biology at Edinburgh, University of Edinburgh, Edinburgh, United Kingdom; 9 Max von Pettenkofer Institut, Ludwig-Maximilians Universität München, München, Germany; University of Southern California Keck School of Medicine, United States of America

## Abstract

Herpes simplex virus type 1 (HSV-1) is a neurotropic virus causing vesicular oral or genital skin lesions, meningitis and other diseases particularly harmful in immunocompromised individuals. To comprehensively investigate the complex interaction between HSV-1 and its host we combined two genome-scale screens for host factors (HFs) involved in virus replication. A yeast two-hybrid screen for protein interactions and a RNA interference (RNAi) screen with a druggable genome small interfering RNA (siRNA) library confirmed existing and identified novel HFs which functionally influence HSV-1 infection. Bioinformatic analyses found the 358 HFs were enriched for several pathways and multi-protein complexes. Of particular interest was the identification of Med23 as a strongly anti-viral component of the largely pro-viral Mediator complex, which links specific transcription factors to RNA polymerase II. The anti-viral effect of Med23 on HSV-1 replication was confirmed in gain-of-function gene overexpression experiments, and this inhibitory effect was specific to HSV-1, as a range of other viruses including Vaccinia virus and Semliki Forest virus were unaffected by Med23 depletion. We found Med23 significantly upregulated expression of the type III interferon family (IFN-λ) at the mRNA and protein level by directly interacting with the transcription factor IRF7. The synergistic effect of Med23 and IRF7 on IFN-λ induction suggests this is the major transcription factor for IFN-λ expression. Genotypic analysis of patients suffering recurrent orofacial HSV-1 outbreaks, previously shown to be deficient in IFN-λ secretion, found a significant correlation with a single nucleotide polymorphism in the IFN-λ3 (IL28b) promoter strongly linked to Hepatitis C disease and treatment outcome. This paper describes a link between Med23 and IFN-λ, provides evidence for the crucial role of IFN-λ in HSV-1 immune control, and highlights the power of integrative genome-scale approaches to identify HFs critical for disease progression and outcome.

## Author Summary

Herpes simplex virus type 1 (HSV-1) infects the vast majority of the global population. Whilst most people experience the relatively mild symptoms of cold sores, some individuals suffer more serious diseases like viral meningitis and encephalitis. HSV-1 is also becoming more common as a cause of genital herpes, traditionally associated with HSV-2 infection. Co-infection with HSV-2 is a major contributor to HIV transmission, so a better understanding of HSV-1/HSV-2 disease has wide implications for global healthcare. After initial infection, all herpesviruses have the ability to remain dormant, and can awaken to cause a symptomatic infection at any stage. Whether the virus remains dormant or active is the result of a finely tuned balance between our immune system and evasion techniques developed by the virus. In this study we have found a new method by which the replication of the virus is counteracted. The cellular protein Med23 was found to actively induce an innate anti-viral immune response in the form of the Type III interferons (IFN-lambda), by binding IRF7, a key regulator of interferons, and modulating its activity. Interferon lambda is well known to be important in the control of Hepatitis C infection, and a genetic mutation correlating to an increase in interferon lambda levels is strongly linked to clearance of infection. Here we find the same association between this genetic mutation and the clinical severity of recurrent cases of HSV-1 infection (coldsores). These data identify a Med23-interferon lambda regulatory axis of innate immunity, show that interferon lambda plays a significant role in HSV-1 infection, and contribute to the expanding evidence for interferon lambda in disease control.

## Introduction

Up to 90% of the global population is infected with the α-herpesvirus Herpes simplex virus type I (HSV-1). Whilst HSV-1 is largely responsible for outbreaks of vesicular oral skin lesions (fever blisters, or cold sores), it can also cause a variety of more severe diseases including encephalitis, meningitis and keratitis [1], [2]. Furthermore, the frequency of association with genital lesions (previously associated mainly with HSV-2 infection) is increasing. As co-infection with HSV is a significant contributing factor to transmission of the Human Immunodeficiency Virus (HIV), our understanding of HSV disease, and herpesviruses in general, has wide implications for global healthcare.

Like all herpesviruses, HSV-1 establishes lytic (epithelial cells) and asymptomatic latent infection (sensory neurons in trigeminal and sacral ganglia) which undergoes periodic reactivation [3]. The equilibrium between these two infection states requires a fine balance between innate and adaptive immune responses, and viral immune evasion mechanisms [4]. Whilst aspects of the HSV-1 replication cycle have been intensively investigated, there remain gaps in our understanding of the complexity of virus:host interactions. For example, a proteomics study identified over 100 changes in the cellular proteome within the first 6h of infection with HSV-1 [5], and a recent analysis of virion-incorporated cellular proteins found that about 30% of these directly affected virus growth [6].

To systematically identify host factors (HFs) required for viral replication, RNAi screens have been performed with a range of different RNA and DNA viruses including HIV-1 [7], [8], [9], Influenza A virus [10], [11], [12], Hepatitis C virus [13], West Nile virus [14], Dengue virus [15], Enterovirus [16] and Vaccinia virus [17], [18]. The overlap between the results of these studies is generally very low [19], reflecting either differences in biology, or different experimental set-ups, cutoff and selection criteria. In addition, microenvironmental effects might also play a role for the differences of the results [20].

Whilst loss-of-function siRNA screens provide functional information on specific genes, protein interaction studies can provide insight into the mechanism of action by identifying physical interaction partners between pathogen and host. Genome-scale virus-host protein interaction screens using the yeast-two-hybrid system have been performed for HCV [21], Influenza A virus [22], Epstein Barr virus (EBV) [23], Vaccinia virus [24], [25], SARS coronavirus [26] and several non-human viruses [27]. Based on these genome-scale studies and individual interactions found by literature curation, several virus-host interaction databases have been created including the HIV-1, human protein interaction database at NCBI [28], VirHostNet [29], VirusMINT [30], PIG [31] and HPIDB [32]. Although there is little overlap between individual cellular interactors of different viruses, targeting of a number of cellular processes such as cell cycle regulation, nuclear transport and immune response appears to be conserved [33].

Understanding the complex interplay between viral and host components is critical to the definition of herpesvirus infection and pathogenesis. As herpesviruses encode a large number of proteins, in contrast to small RNA viruses such as HIV and Influenza, many cellular processes may be directly affected by viral proteins, and whilst there exists a wealth of information on individual viral proteins, there remain large gaps in our understanding of the HSV-1 life cycle and its interaction with its host. Here, we present data from the first integrative and systematic screening approach to characterise the role of cellular proteins in the HSV-1 life cycle. A genome-scale RNAi knockdown screen to identify HFs functionally influencing HSV-1 replication was performed in parallel with a yeast two-hybrid (Y2H) protein interaction screen to simultaneously gain insight into potential mechanisms of action. Combined analyses confirmed the importance of known cellular proteins involved in processes such as cell cycle, proteins transport and gene expression important for virus replication. Furthermore, we identified a subunit of the Mediator multi-protein complex, Med23, as a key regulator of IFN-λ induction, which appears to be of crucial significance for the control of HSV-1 both *in vitro* and *in vivo*. These data demonstrate the power of a combined screening strategy to investigate pathogen:host interactions and identify novel host factors and cellular pathway targets for the development of essential clinical interventions.

## Results

### A siRNA depletion screen identifies host factors (HFs) for HSV-1 replication

**Figure 1:**
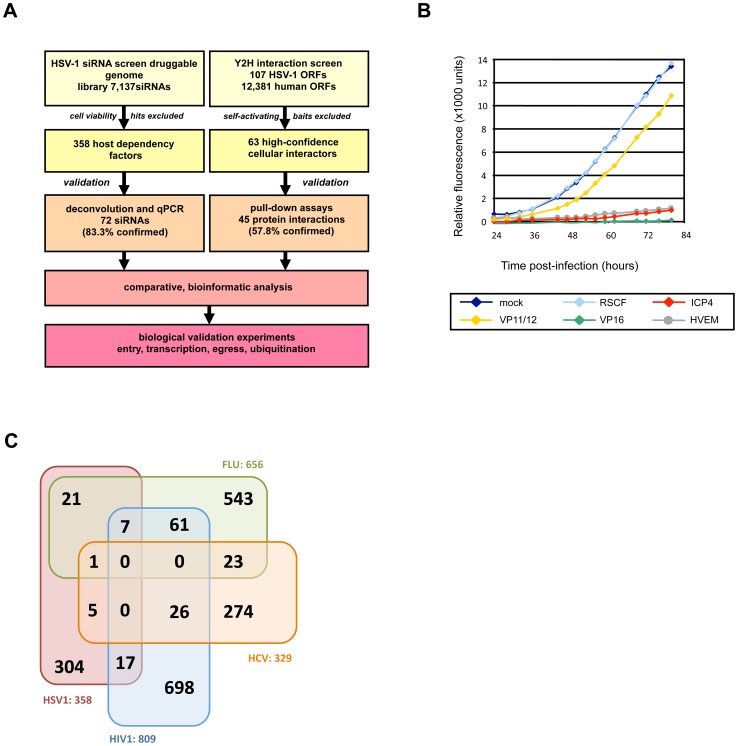
Identification of HSV-1 host factors by RNAi and Y2H screens. (a) Strategy to identify host factors interacting with viral proteins or influencing HSV-1 replication. (b) RNAi perturbation screen by kinetic analysis of HSV-1 replication. Hela cells were reverse-transfected with siRNA SMARTpools (4 siRNAs per gene). After 48 h the siRNAs were tested for cytotoxicity (3 replicates) or the capacity to influence replication of the HSV-1 GFP reporter virus C12 (6 replicates) from 24 to 80 h post-infection. Virus replication slopes during the linear phase were calculated and normalized to mock-transfected cells. Replication slopes were then compared to replication upon knockdown of essential (ICP4, VP16) or non-essential (VP11/12) viral genes, a cellular receptor for HSV-1 (HVEM) or control RISC-free siRNA (RSCF). (c) Overlap between the HSV-1 HFs identified in this study with those published in HIV-1 [7], [8], [9], Hepatitis C Virus (HCV) [13] and Influenza A virus [10], [11], [12].

Host factors (HFs) which positively or negatively regulate HSV-1 replication were identified by screening a druggable genome siRNA library (4 siRNAs per gene) targeting 7,237 human genes against a HSV-1 reporter virus expressing the enhanced green fluorescent protein (eGFP; HSV-1 strain C12) in the epithelial Hela cell line, due to their ease of transfection and susceptibility to HSV-1 infection [34]. To generate a robust and reliable dataset the screen was carried out three times in triplicate, with one replicate used in a cell viability assay to determine any cytotoxic effects of gene depletion and duplicates infected for the virus infection assay. The siRNA library was reverse-transfected into Hela cells before infecting with HSV-1 and monitoring virus growth kinetics as a measure of GFP-fluorescence (**Figure 1b**). By following virus growth over multiple rounds of replication, host proteins involved in all stages of the virus life cycle can be identified. Replication slopes during linear growth were normalized to controls (mock-transfected cells, and cells transfected with a siRNA unable to be processed by the RNA Silencing Complex, RSCF) and the mean of six replicates was calculated. siRNAs found to be cytotoxic (81 in total) were excluded from further analyses, and a hitlist of 358 containing the top 2.5% inhibitory and the top 2.5% enhancing HFs was generated (**Table S1 in Text S2**). The identified HSV-1 HFs were compared to datasets from published siRNA depletion screens aimed at identifying cellular factors affecting HIV-1 [7], [8], [9], West Nile Virus (WNV) [14], Hepatitis C Virus (HCV) [13], Dengue virus [15] and Influenza A virus [10], [11], [12]. Of our 358 HFs, 54 cellular proteins (15.1%) overlapped with these other virus screens (Influenza A, 29; HIV-1, 24; HCV, 6; WNV, 2; Dengue virus, 1) (**Figure 1c** **; Table S2 in Text S2**).

### A genome-scale yeast two-hybrid protein interaction screen identifies novel viral protein interaction partners

HSV-1 is currently known to encode at least 84 proteins, expressed sequentially under strict temporal regulation during infection. To gain further mechanistic insight into host factors involved in HSV-1 infection, in parallel to the siRNA depletion screen we carried out a yeast two-hybrid protein interaction screen to identify cellular interaction partners of viral proteins. We generated a collection of 107 partial and full-length HSV-1 cDNA constructs and tested them for interactions with proteins encoded by a library of 12,381 human cDNA clones [35]. 231 HSV-1-human protein interactions were detected once (low confidence), and 63 more than once (high-confidence)(**Table S3 in Text S2**). Using these high-confidence interactions, the previously reported HSV-1 interactome [36] was connected into a human interactome (62,310 published protein interactions) to generate a combined pathogen-host interactome (**Figure S1a**). Both degree centrality (which indicates the number of interactions a protein has, where high values represent highly interactive ‘hubs’) and betweenness centrality (which indicates the number of shortest paths between any pair of proteins passing through the protein considered) were significantly increased for HSV-1 interactors, particularly in the high-confidence network (**Figure S1b–e**). These data suggest HSV-1 proteins preferentially target highly connected central human proteins in the cellular interaction network, similar to other viruses [23].

Analysis of this interactome for HFs identified by RNAi found they were enriched in the fraction of cellular proteins that directly interact with viral proteins or that interact via one intermediate, in comparison to proteins that only interact via 2 or more intermediates (*p* = 0.036, Fisher's exact test)(**Figure S1f**). A direct comparison of HSV-1 protein interaction partners and the siRNA screen HFs found 215 genes in common. Of those, ten (4.6%) were identified as a hit in both screens (**Table S4 in Text S2**), suggesting that these technologies identify complimentary yet not necessarily overlapping HFs.

### Validity and specificity of HSV-1 HFs

**Figure 2:**
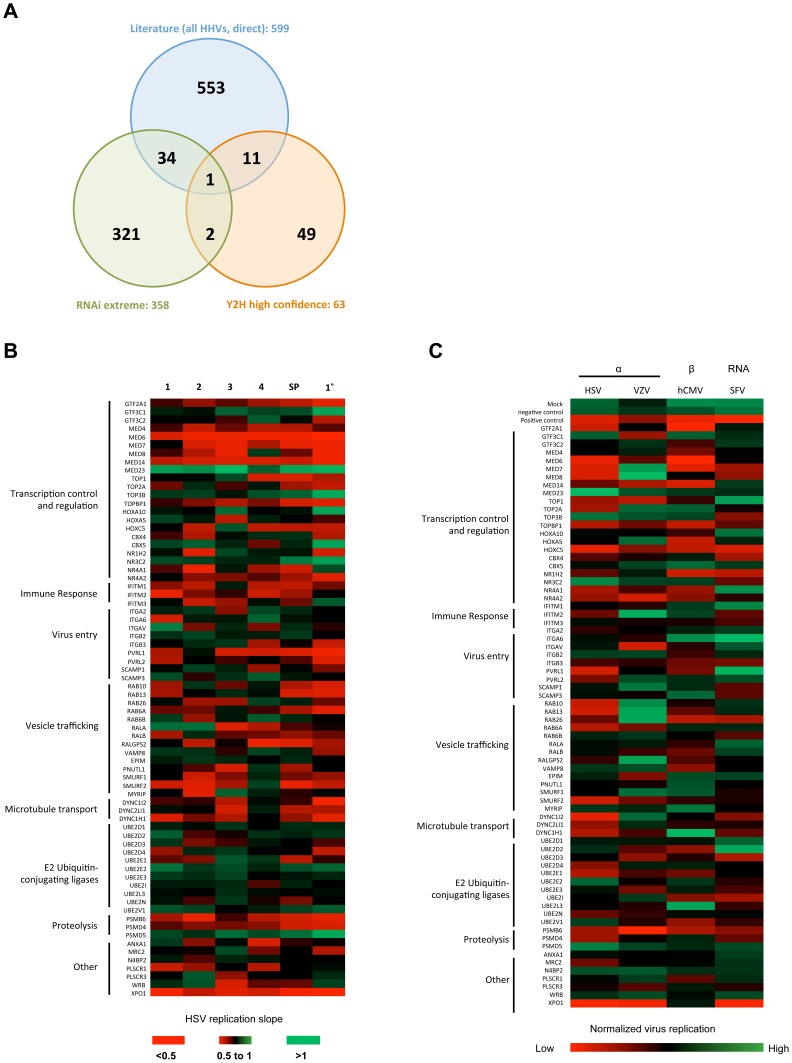
Primary validation of HFs for HSV-1. (a) Overlap between HSV-1 HFs, cellular protein interactors of HSV-1 proteins identified by Y2H system, and published protein interactors of all human herpesviral proteins. (b) Validation of a subset of HFs by siRNA deconvolution. A subset of HFs was selected for validation with deconvoluted siRNAs to confirm the phenotype observed in the primary screen. The effect of the four individual siRNAs (1–4) and a reconstituted SMARTpool (SP) were tested by reverse-transfecting into Hela cells before infecting after 48 h with HSV-1-eGFP (C12) and monitoring replication. Replication slopes were calculated and normalized as described, and compared to the primary screen slope (1°). A heat map of replication slopes was generated where red represents inhibition (replication slope <0.5) and green represents enhancement (slope >1). The phenotype was considered validated if ≥2 siRNAs produced the same or better phenotype as the primary screen. (c) Virus specificity of HSV-1 HFs. The effect of HF siRNA SMARTpools on the replication of VZV (α-herpesvirus), hCMV (β-herpesvirus) or Semliki Forest virus (SFV; RNA virus) was determined and compared to HSV-1. Normalized replication ±2×STDEV of the controls was considered inhibiting/enhancing.

An extended literature and database search identified 599 cellular proteins that interact with or are involved in infection with human herpesviruses. The overlap between the high-confidence Y2H cellular interactors (63) and HFs (358) with this set was statistically significant (*p = *0.008) (**Figure 2a** **; Figure S1h; Table S5 in Text S2**). From this combined analysis, a subset of HFs was chosen for further validation. Protein interactions were tested in a mammalian cell system by LUMIER pull-down assay [37]. Of the 45 interactions tested, 26 (57.8%) were confirmed, with 15 strongly positive (z-score >2) and 11 weakly positive (z-score 1–2) (**Figure S1g**). siRNA deconvolution (4 siRNAs per gene tested individually) was used to further validate 72 HFs (**Figure 2b** **; Figure S2**). The replication phenotype could be confirmed (≥2 or more siRNAs gave the same or better replication slope than observed in the primary screen) in a high proportion (83.3%) of candidates, highlighting the reliability of the primary screen dataset. Quantitative RT-PCR analysis of mRNA expression levels found a minimum depletion of 60% (mean 88%) in a subset of 52 genes (data not shown; **Table S6 in Text S2**) confirming the observed effects on HSV-1 replication are genuine and not due to ‘off-target’ effects or insufficient gene knockdown. To further investigate the virus-specificity of our identified HFs, we tested this subset for their effect on the replication of an additional α-herpesvirus (Varicella-Zoster virus, VZV), the β-herpesvirus Cytomegalovirus (CMV), and a completely unrelated RNA virus, Semliki Forest Virus (SFV). None of the three proteins which enhanced HSV-1 replication upon knockdown had an effect on either VZV or CMV, and one (*NR3C2*) was even inhibitory for SFV (**Figure 2c**). Of the 64 siRNAs which inhibited HSV-1, 27 (42.2%) were also inhibitory for VZV, 60 for CMV (93.8%) and 23 (35.9%) for SFV replication (**Table S7 in Text S2**). Some functional groups (transcriptional regulators) were required by most viruses, but there were notable differences between other proteins. For example IFITM-1, previously identified as an inhibitor of Influenza A, Dengue virus and WNV [10], inhibited VZV yet had a positive effect on HSV-1 replication. These data suggest that whilst there are some HFs which are broad in their effects on virus replication, a large proportion are species-specific.

### HSV-1 HFs are involved in diverse cellular pathways and at multiple stages of the HSV-1 life cycle

Functional and pathway analysis of the 358 HSV-1 HFs identified in the siRNA depletion screen (**Figure S3a**), and of direct and indirect virus-host interactions with multiple interaction partners (**Figure S3b**), found a significant enrichment of a wide range of cellular processes involved in multiple stages of virus replication (**Table S8 in Text S2**). Pathways included those involved in gene expression, transcription, splicing and translational regulation (RNAi screen), and protein transport, cell cycle, and transcriptional repressor activity (Y2H screen). A combined analysis of HFs from both screens found dominant functional categories centred on the regulation of transcription (RNA polymerase II-associated genes, splicing factors, transcription activation and the Mediator complex) (**Figure S3c, d**). The physiological relevance of some HFs and pathways was confirmed by further biological validation. Protein transport pathways (in the form of dynein microtubule networks) are exploited by HSV-1 early after infection to shuttle viral capsids to the nucleus. These screens confirmed known interactions between dynein subunits and viral proteins, and identified additional previously unknown interactions (**Text S1 and** **Figure S4a**). Several dynein chain subunits were found to be essential for virus replication, whilst the moderate effect of depletion of other subunits demonstrated a level of functional redundancy in HSV-1 capsid transport (**Figure S4b–e**) [38], [39]. Intrinsic anti-viral host defense mechanisms, in the context of cellular E2 ubiquitin ligases, were also investigated. The immediate-early viral protein ICP0, an E3 ubiquitin ligase, is crucial for blocking anti-viral defense mechanisms by degrading promyeloctic leukemia (PML) nuclear bodies (ND10 domains) in the presence of cellular E2-ubiquitin-conjugating enzymes (E2s). Our siRNA screen found multiple E2s were required for this, and suggests that HSV-1 ICP0 is promiscuous in its exploitation of E2s to mediate PML degradation and ensure successful infection (**Text S1 and Figure S5**).

### Med23 is an anti-viral component of the pro-viral Mediator complex

**Figure 3:**
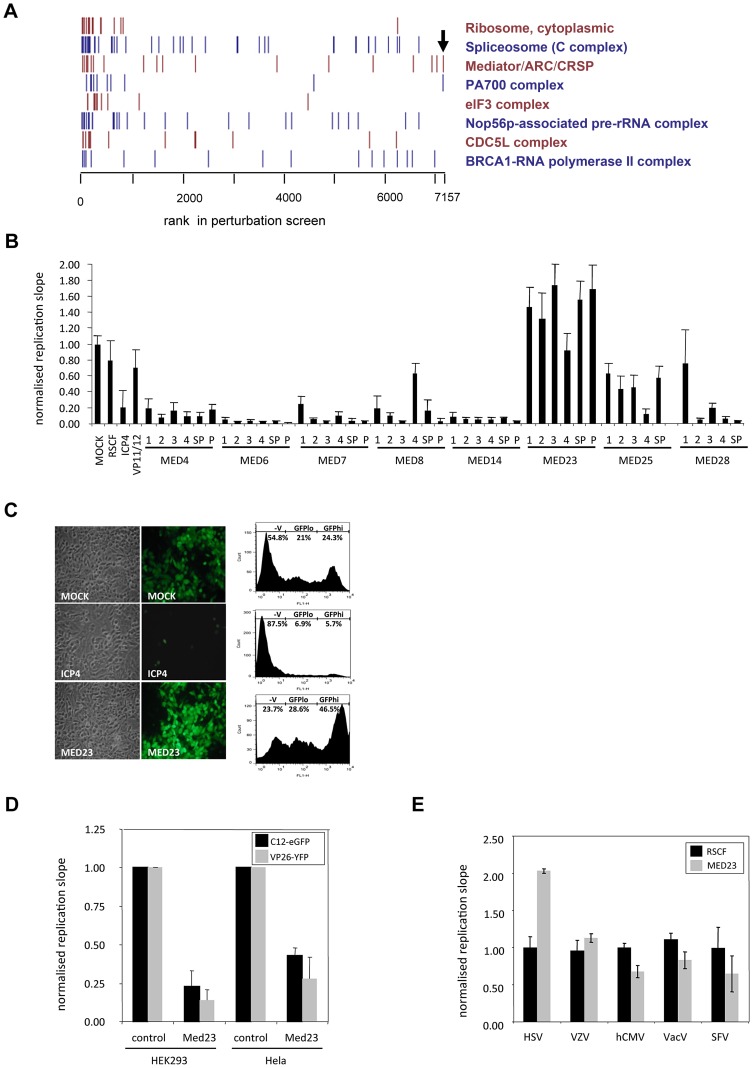
Med23 is a novel anti-viral HF against HSV-1. (a) Multiprotein complexes involved in HSV-1 replication. The distribution of replication slopes of components from eight human protein complexes found to be critical to HSV-1 infection were ranked inhibiting (Rank 1, left) to enhancing (Rank 7157, right). Arrow denotes Med23. (b) Deconvoluted siRNAs confirm role of Mediator subunits in HSV-1 replication. The effect of four individual siRNAs (1–4) and a reconstituted SMARTpool (SP) on a range of subunits of the Mediator complex were compared to the primary screen (P). Med25 was not present in the primary screen so has no comparative ‘P’. Hela cells were transfected and infected with HSV-1-eGFP. Replication slopes were calculated and normalized to controls. Error bars represent the mean of three independent experiments done in duplicate. (c) Fluorescence microscopy and FACS analysis of Hela cells depleted of Med23. Hela cells transfected with either ICP4 or Med23 siRNA SMARTpools were infected with the recombinant HSV-1 GFP reporter virus C12 (MOI 1) and analysed by fluorescence microscopy and FACS analysis. Numbers indicate the percentage of cells in the uninfected, GFP^lo^ or GFP^hi^ populations. (d) Overexpression of Med23 inhibits HSV-1 C12-GFP and VP26-YFP. Hela or HEK cells overexpressing Med23 transiently (Hela) or stably (HEK) were infected with HSV-1 C12-eGFP or HSV-1 VP26-YFP at MOI 0.5. Replication slopes were monitored and normalized to control (pCR3)-transfected cells. Error bars represent the mean of at least three independent experiments. (e) Depletion of Med23 specifically affects HSV-1. Hela cells were depleted for Med23 with a siRNA SMARTPool and infected with HSV-1, Varicella zoster virus (VZV), human cytomegalovirus (hCMV), Vaccinia virus (VacV) or Semliki Forest Virus (SFV). Replication slopes (HSV-1, VZV, hCMV, VacV) or endpoint replication values (SFV) were calculated and normalized to controls. Error bars represent the mean of at least three independent experiments.

Combined bioinformatic analyses of protein interaction and siRNA depletion screens found a significant functional enrichment for proteins involved in transcription, and identified multi-protein complexes enriched for pro-viral HFs which strongly inhibited HSV-1 upon depletion, including the RNA-polymerase II, eIF3 and Mediator complexes (**Figure 3a**). The Mediator complex links the cellular transcription machinery (RNA polymerase II) to specific transcription factors, and the identification of many Mediator subunits as HFs in other viral siRNA depletion screens highlights its significant role in viral genome transcription [7], [9], [11], [40] (**Table S2 in Text S2**). Further, several Mediator subunits (Med25, 29, 17 and 8) are known to interact with the HSV-1 transactivator VP16 (UL48) and other herpesviral proteins [41] (**Figure S6a**). Consistently, the Mediator complex was found to be strongly required for HSV-1 replication, with depletion of the majority of subunits (Med 4, 6, 7, 8, 14, 16, 17, 21, 25, 26, 27 and 28) leading to a severe reduction in virus replication in the primary screen (**Figure S6b**) or in confirmatory deconvolution assays (**Figure 3b**). However, depletion of the Med23 subunit was striking in that it led to a significant enhancement of virus growth (**Figure 3b**). Flow cytometry quantification found that removal of Med23 not only increased the total number of infected cells (combination of GFP^lo^ and GFP^hi^ cells; 75.5% in comparison to 49.6% in mock-transfected cells) but also the copy number of virus genomes (GFP^hi^ cells; 44.8% in comparison to 24.4% in mock-transfected cells) (**Figure 3c**). Gain-of-function experiments found overexpression of Med23 led to a corresponding inhibition of two strains of HSV-1 (**Figure 3d** **; Figure S6c**), confirming Med23 is a natural anti-viral component of the pro-viral Mediator complex. This anti-viral effect of Med23 was specific for HSV-1, as replication of VZV (α-herpesvirus), hCMV (β-herpesvirus), Vaccinia virus (DNA) and SFV (RNA virus) remained unaffected by Med23 depletion (**Figure 3e**).

### Med23 inhibits HSV-1 replication by inducing a type III interferon (IFN-λ) response

**Figure 4:**
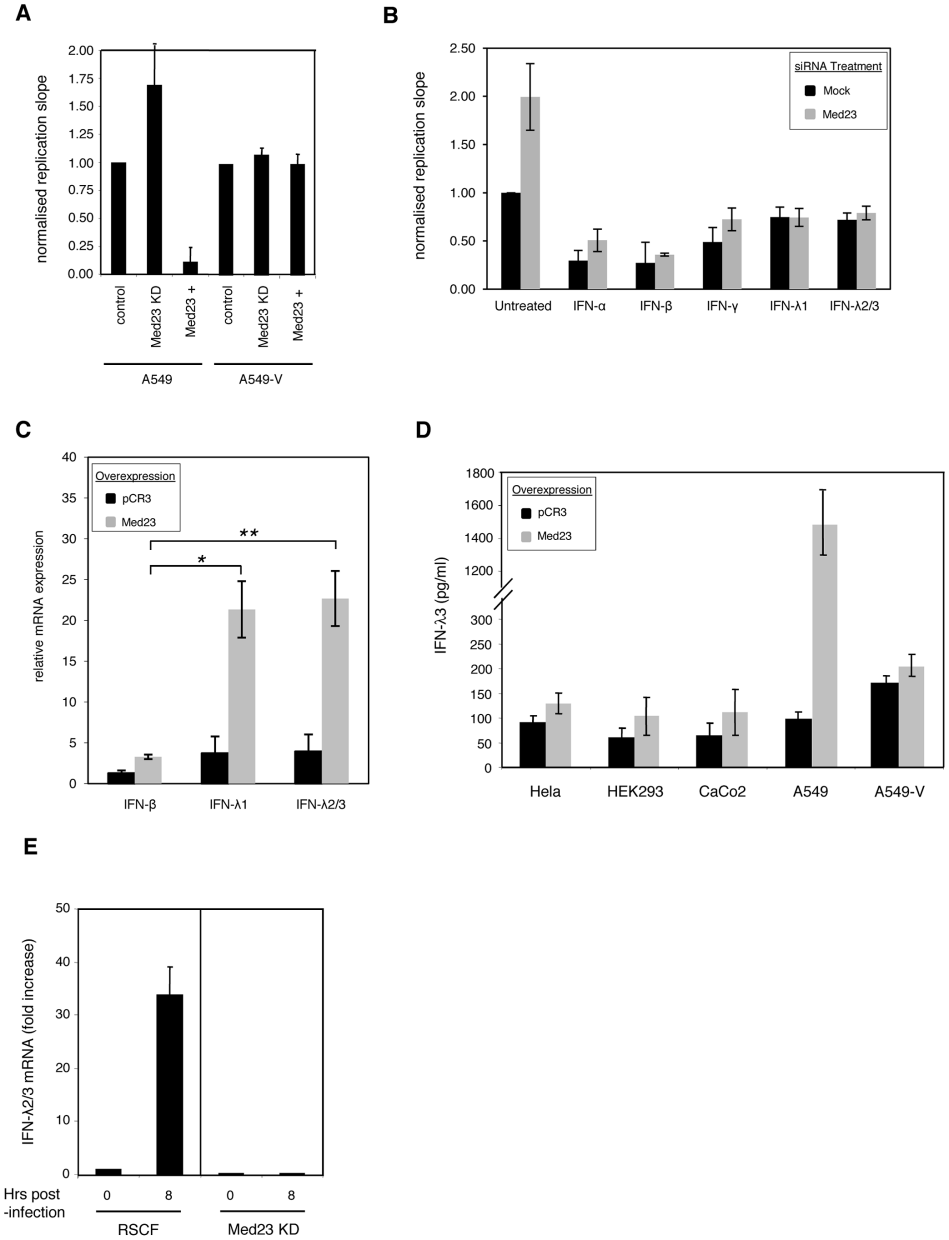
Med23 inhibits HSV-1 by inducing a Type III interferon (IFN-λ) response. (a) Med23 depletion or over-expression has no effect in A549-V cells deficient in Jak/Stat signalling. A549 cells and derivative A549-V cells were transfected with Med23 siRNA SMARTpool (Med23 KD) or a pCR3-Med23 overexpression plasmid (Med23+) 48 h (siRNA) or 24 h (pCR3) before infection with HSV-1-eGFP C12. Replication was monitored, and slopes calculated and normalized to controls (RSCF siRNA or pCR3). Error bars represent the mean of at least three independent experiments. (b) Pre-treatment with Type III interferons prevents Med23-mediated enhancement of HSV-1 replication. A549 cells were mock-transfected or transfected with Med23 siRNA. After 48 h cells were untreated or pre-treated with 50 ng/ml IFN-α, IFN-β, IFN-γ or 100 ng/ml IFN-λ1 or IFN-λ2/3 for 6 h, before infecting with HSV-1-eGFP C12. Replication was monitored and slopes calculated and normalized to unstimulated, mock-transfected cells. (c) Overexpression of Med23 preferentially induces type III interferons. pCR3 or Med23 were overexpressed in A549 cells and induction of type I (IFN-β) and type III (IFN-λ1, IFN-λ2/3) was measured by qRT-PCR. mRNA levels were normalized to HPRT and calibrated to mock transfected cells (control). Error bars represent the mean of technical replicates and is representative of multiple experiments. * = *p*-value 0.003; ** = *p*-value 0.002 (unpaired t-tests for unequal variances). (d) Overexpression of Med23 induces IFN-λ secretion. A range of cell types were transfected with pCR3 or Med23, supernatant harvested 120 h post-transfection and IFN-λ3 levels measured by ELISA. Chart shows the mean and standard deviation of duplicates over two experiments. (e) siRNA depletion of Med23 inhibits the induction of IFN-λ2/3 following HSV-1 infection. A549 cells were transfected with control (RSCF) or Med23-specific siRNA (Med23 KD) before infecting with HSV-1-eGFP C12 (MOI 0.5). RNA was harvested 0 or 8 h post-infection and IFN-λ2/3 mRNA levels measured by qRT-PCR. Expression was normalized as above, and calibrated to RSCF-transfected cells at 0 h post-infection. Error bars represent the mean of technical replicates and is representative of multiple experiments.

Med23 could exert anti-viral effects either by having an inhibitory effect on viral transactivators or by interacting with and having a positive effect on an existing anti-viral factor. We first tested whether Med23 directly affects viral gene expression using luciferase reporters with HSV-1 promoters, however observed no inhibitory effect (data not shown). Since the Mediator complex and Med23 in particular is known to be involved in Jak/Stat-mediated interferon signaling [42], we used the lung epithelial cell line A549 and its Stat-1-deficient derivative A549-V [43] to determine if Med23 influences HSV-1 replication by modulating innate immunity. In the parental A549 cells the phenotype of HSV-1 replication was the same as that observed in Hela cells, where depletion of Med23 enhanced replication and over-expression inhibited virus growth. However, in the Stat1-deficient A549-V cells HSV-1 replication was unaffected by both depletion and over-expression of Med23 (**Fig. 4a**), indicating that Med23 requires an intact Jak/Stat signalling pathway to exert its anti-viral effects.

To determine which interferon may be responsible for the anti-viral effects of Med23, A549 cells were depleted for Med23 and infected with HSV-1 following pre-stimulation with Type I (IFN-α or IFN-β), Type II (IFN-γ) or Type III (the distinct IFN-λ1 or the almost identical IFN-λ2 and -λ3, termed IFN-λ2/3) interferons. Whilst treatment with IFN-α, -β and -γ significantly decreased HSV-1 replication levels, the observed ∼2-fold enhancement of HSV-1 replication following Med23 depletion was still seen. However, pre-treatment with the both IFN-λ1 and IFN-λ2/3 blocked the enhancing effect of Med23 depletion (**Figure 4b**). Investigation into the effect of Med23 on interferon induction by qRT-PCR found that whilst Med23 over-expression induced IFN-β (∼3-fold increase), induction of IFN-λ1 and λ2/3 was considerably and statistically significantly higher (∼26-fold induction; *p* = 0.003 and 0.002, respectively) (**Figure 4c**). This induction was specific, as levels of other cytokines and interferon-regulatory factors (IRFs) were unaffected by Med23 overexpression (**Figure S7a**). Secretion of IFN-λ2/3 protein was also increased in all cell lines tested, but most significantly to ∼11-fold in A549 cells (**Figure 4d**), which is consistent with a recent report showing that type III interferons are the dominant type of IFNs expressed by primary airway epithelial cells [44]. Furthermore, qPCR analysis found depletion of Med23 inhibited the induction of IFN-λ expression following HSV-1 infection of A549 cells in comparison to cells transfected with the RSCF siRNA control (**Figure 4e**). Together, these data suggest that IFN-λ is responsible for the observed inhibitory effect of Med23 on HSV-1 replication.

### IFN-λ is synergistically induced following a direct interaction between Med23 and IRF7

**Figure 5:**
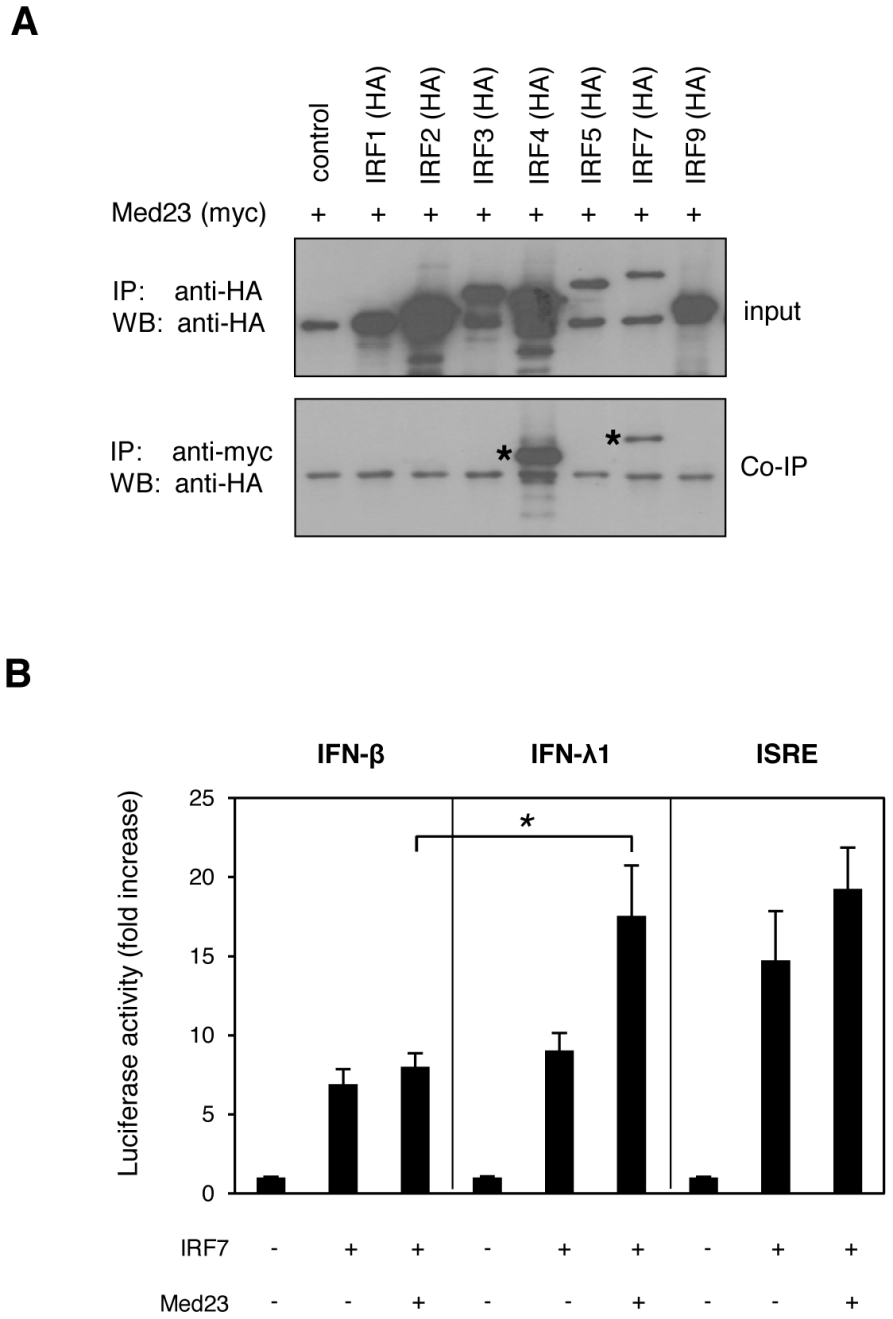
Med23 induces IFN-λ by interacting with the transcription factor IRF7. (a) Med23 directly interacts with IRFs. Med23 was overexpressed in HEK cells as a myc-tagged fusion protein individually with a range of HA-tagged IRFs. Protein amounts were quantified and equal amounts (325 µg) were immunoprecipitated (IP) with anti-HA or anti-myc antibody before western blot analysis and staining with anti-HA (WB) to confirm protein expression (IP; anti-HA IP) and identify interaction partners (Co-IP; anti-myc IP) (b) Med23 synergistically induces IRF7-responsive promoters. A549 cells were transfected with IFN-λ1-, IFN-β- or ISRE-responsive luciferase reporter constructs with IRF7 alone or in addition to Med23. Promoter activity was determined by measurement of Firefly luciferase activity 33 hr post-transfection, and normalized to Renilla luciferase and pCR3-transfected cells. Error bars represent the mean of at least three independent experiments. Statistical significance of the synergistic increase in IFN-λ induction by Med23 with IRF7 over IFN-β induction was determined by unpaired t-tests for unequal variances. * = *p-value* 0.02.

As IFN-λ expression is induced following activation of pathogen recognition receptors (PRRs) by virus infection [45], [46], [47], [48], we tested whether Med23 induced IFN-λ by directly interacting with an interferon-responsive transcription factor (IRF). Y2H and confirmatory co-immunoprecipitation experiments in mammalian cells with a panel of IRFs found that Med23 interacted with IRF4 and IRF7 (**Figure 5a** **; Figure S7b**). We also observed a weak interaction with IRF9, which may explain the previously observed effect of Med23 on Jak/Stat signalling [42]. To determine if this interaction had a functional effect, we looked at whether Med23 influenced IRF-mediated induction of IFN-λ. In a luciferase reporter assay, neither IRF4 nor IRF9 led to a significant induction of the IFN-λ1 promoter, either alone or in conjunction with Med23 (data not shown). IRF7 induced expression from the IFN-β and IFN-λ1 promoters to similar levels (∼7-fold and 9-fold higher than background, respectively), whilst the ISRE, induced by IRF7 and also present in the IRF7 promoter, was induced ∼15-fold (**Figure 5b**). Whilst co-expression of Med23 with IRF7 had no further effect on IFN-β expression, a synergistic induction of the IFN-λ1 promoter and, to a lesser extent, the ISRE, was observed (IFN-λ1 doubled to ∼18-fold, *p = *0.02) (**Figure 5b**). Interestingly, a Med23 mutant unable to induce immediate early gene expression via jun/fos (R617Q, or R611Q in Med23 transcript variant 1 used here) synergistically induced ISRE expression with IRF7, yet was unable to further enhance IRF7-mediated induction of IFN-λ1 (data not shown). A similar synergistic effect of Med23 and IRF7 was seen at the protein level, where co-expression increased supernatant levels of IFN-λ3 more than 2-fold those seen with Med23 or IRF7 alone (**Figure S7c, d**).

### IFN-λ gene expression is associated with the recurrence and severity of recurrent HSV-1 disease

**Figure 6:**
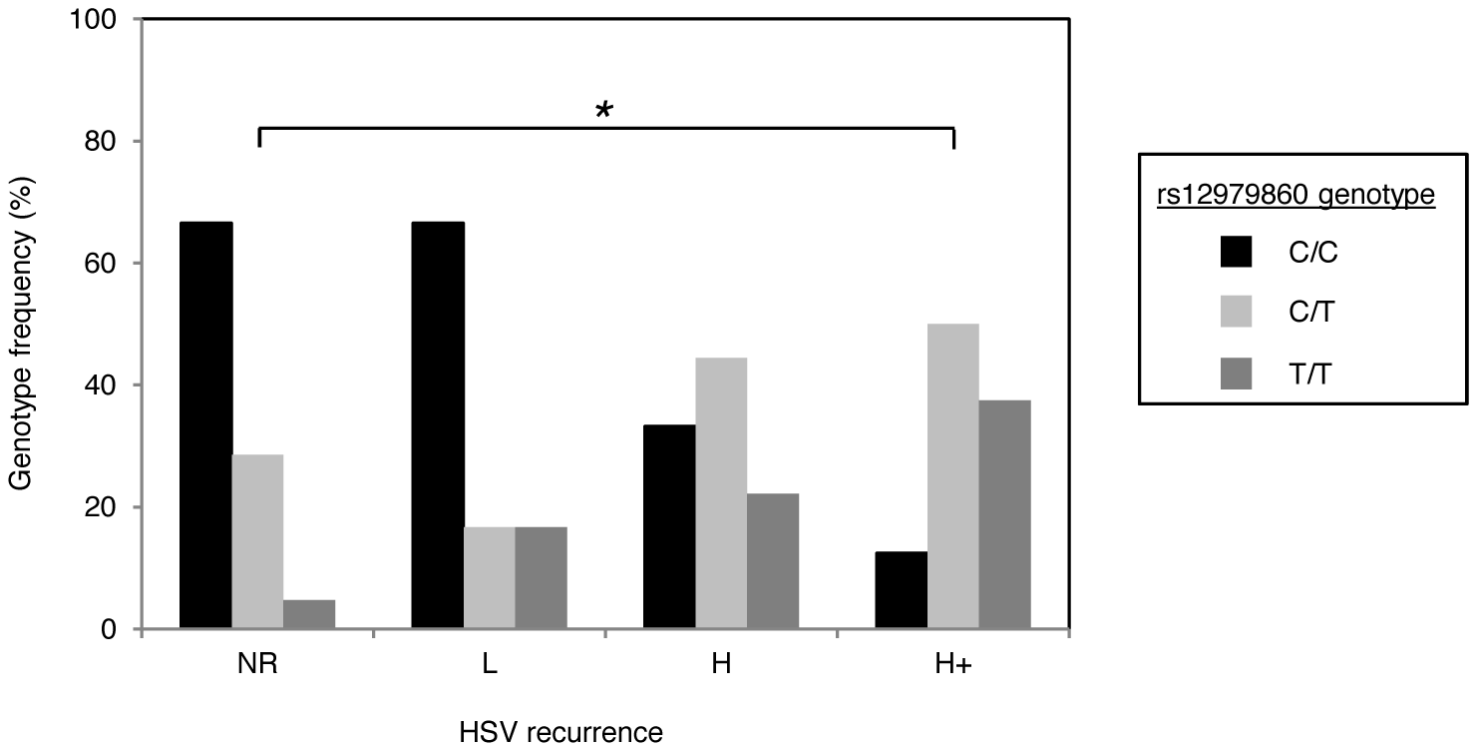
Occurrence and severity of clinical HSV-1 reactivation is associated with IFN-λ expression. The frequency of genotype of the single nucleotide polymorphism rs12979860 in the IFN-λ3 promoter region in HSV-1 individuals with a range of frequency and severity oral herpes labialis was determined by PCR. NR, non-recurrent; L, low recurrence; H, high recurrence; H+ high and clinically severe recurrence. Significance of genotype association was determined by Fisher's exact test, comparing the frequency of the CC, CT or TT genotype in the NR vs the L, H or H+ clinical groups. * = *p*-value 0.014. See also Table 1 and Methods for further details.

Successful disease and treatment outcome in Hepatitis C virus infection (demonstration of a sustained virologic response) is strongly associated with a single nucleotide polymorphism (SNP) in the IFN-λ3 promoter (rs12979860; CC genotype over CT or TT) and higher plasma levels of IFN-λ3 [49],[50]. Furthermore, IFN-λ expression is impaired in a cohort of ethnically Italian individuals suffering recurrent HSV-1-related herpes labialis reactivation [51]. To determine if the clinical severity of HSV-1 disease is due to the observed deficiency in IFN-λ expression, we screened a subset of the recurrent herpes labialis (HL) cohort and additional subjects for the IFN-λ3 promoter polymorphism. Genotypic analysis found the presence of a T (CT or TT genotype) had a dose-dependent association with clinical severity, with the homozygous TT genotype being more prevalent as disease severity increases (**Figure 6**). In spite of the relatively small sample numbers in some clinical categories (**Table 1**), the association of a CT or TT genotype with the most severe recurrence of herpes labialis (H+) was statistically significant (*p* = 0.014; Fishers's exact t-test). As the CC genotype is directly associated with increased IFN-λ3 levels [51], these data highlight a previously unknown association between the frequency/severity of recurrence of herpes labialis, the CT/TT genotype and subsequent reduction in secretion of IFN-λ3. It is of importance to investigate this genotype association with a larger cohort of HL patients, as well as those suffering with other HSV-1-related disease, in order to determine the role of IFN-λ in the full spectrum of HSV-1 pathogenesis.

**Table 1:** Association of the IL28B genetic polymorphism rs12979860 with recurrence and severity of oral HSV-1 labialis.

						Genotype		
Clinical status^a^	*n* b	Gender^c^	*n* b	Age range	Median age	CC	CT	TT
NR	21	M	5	26–49	39	4	1	0
		F	16	27–52	36	10	5	1
L	18	M	6	22–61	43	4	1	1
		F	12	23–60	37	8	2	2
H	9	M	2	37–52	44.5	2	0	0
		F	7	31–54	42	1	4	2
H+	9	M	3	35–46	35	1	1	1
		F	5	25–55	38	0	3	2

a SN, HSV-1 IgG seronegative; NR, non-recurrent; L, low recurrence; H, high recurrence; H+, high recurrence and clinical severity. See Methods for details.

b n, number of patients.

c M, male; F, female.

Taken together, these data identify Med23 as a novel anti-viral factor which acts as a key regulator of IFN-λ expression by interacting with and enhancing the activity of IRF7, a major transcription factor involved in innate immunity. Our observation of a link between the clinical severity of HSV-1 disease and, a CT/TT genotype at a SNP known to regulate IFN-λ3 secretion demonstrates the significance of IFN-λ in the control of HSV-1 replication *in vivo*. Whilst this study provides no direct link between the IFN-λ3 promoter polymorphism and Med23, these associations of IFN-λ with HSV-1 disease, combined with our observations that Med23 is required for the induction of IFN-λ following HSV-1 infection, identifies for the first time a link between Med23 and IFN-λ, provides a clinical context for Med23 regulation of IFN-λ expression and underscores the potential biological significance of these data.

The use of HSV-1 in a combined genome-scale screening approach has led to the identification of a regulatory axis in anti-viral innate immunity, and this important finding not only highlights the power of such combined genome-scale screening approaches to identify novel host candidates for anti-herpesvirus drug discovery, but provides an invaluable dataset to the herpesvirus and scientific community at large.

## Discussion

By nature of their scale, high-throughput screening technologies have limitations. RNAi technology is limited by technical issues such as off-target effects, where an alternative gene to the intended target is degraded, and insufficient gene knockdown. Similarly, Y2H protein interaction screens can generate both false-positive interactions, due to ‘sticky’ proteins and auto-activation of the reporter gene used, and false-negative interactions. Whereas the number of false positives can be considerably reduced by stringent screening and selection criteria, the low sensitivity of the Y2H assay, which detects 20–30% of known interactions, is inherent to the system and can only be marginally improved. This poor sensitivity is caused by factors such as structural restraints of the Y2H bait and prey fusion proteins, a lack of or existence of distinct protein modification in yeast cells, and cellular localization signals in bait and prey proteins preventing nuclear import [52]. However, as all other high-throughput methods for measuring binary protein interactions possess a similarly low sensitivity, but are considerably more laborious and expensive, the Y2H system is still the most commonly used technology [53].

In this study we have exploited a combined genome-wide screening approach to investigate HSV-1 replication and interaction with its host. This identified 358 functional HFs modulating HSV-1 replication, and 63 cellular interaction partners. In validation experiments, 57.8% of the interactions were confirmed by co-immunoprecipitation assays in mammalian cells, and of the 358 functional HFs identified in the siRNA screen, the phenotype of 83.3% was confirmed in deconvoluted siRNA experiments. This, combined with qPCR data demonstrating a minimum gene depletion of 69%, suggests that the functional phenotypes on virus replication are genuine, and not due to ‘off-target’ effects. The confirmation of such a high proportion of selected validation candidates, in spite of the potential technical drawbacks, highlights the reliability of our primary screen datasets, and thus provides an invaluable resource for the herpesvirology research community.

One interesting outcome of this study was the surprisingly low overlap between hits identified using these different technologies. Of the 215 genes in common between the siRNA and cDNA libraries, only 10 (4.7%) were classified as a hit by both methods. This, however, is not unexpected, as even the overlap between studies using the same technology has been reported to be low. For example, the overlap between the three previously published HIV screens was only 7% [19]. Furthermore, the degree of functional redundancy within the siRNA library, and cellular pathways in general, the potential situation-specificity of virus-host interactions, and the possibility of indirect interactions between viral and host proteins, suggest that these methodologies detecting functional outcomes or physical interactions are linked, but complementary rather than confirmatory.

The identified HFs were enriched for a range of cellular processes, such as transcription, gene expression, protein transport and cell cycle (**Figure S2 and S3**), and involved at different stages of viral infection. We investigated HFs involved in capsid transport and ubiquitination of antiviral intrinsic host defence factors in more detail. Incoming HSV-1 capsids are transported to nuclear pores via the microtubule-organizing centre (MTOC), mediated by capsid proteins VP26 (UL35) and UL46 binding to the dynein light chains DYNLT1 (Tctex1) and DYNLT3 (rp3) [54], [55]. Our Y2H screen confirmed the known interaction between the capsid protein VP26 and the dynein light chain DYNLT3 (**Text S1 and Figure S4**). Combined with the siRNA screen data, which found depletion of multiple light chain subunits had moderate anti-viral effects on HSV-1, these data confirm propositions of redundancy in the capsid transport process which ensures successful infection in the event of viral mutations [38], [39], and provide further evidence that HSV-1 has evolved to be highly promiscuous in its exploitation of cellular pathways to its advantage.

To overcome the intrinsic host defence, HSV-1 induces a proteasome-dependent degradation of anti-viral promyelocytic leukemia (PML) nuclear bodies (ND10 domains) by the RING-finger ubiquitin ligase ICP0 expressed during early infection [56]. *In vitro*, ICP0 is a biochemically active E3 ubiquitin ligase in the presence of E2 ubiquitin conjugating enzymes (E2s) [UBE2D1 (UbcH5a) and UBE2E1 (UbcH6)] [57], but which E2s are used during infection has remained unclear. We identified 20 cellular E2s that are able to influence HSV-1 replication (**Text S1 and** **Figure S5**). Depletion of UBE2D1-4, UBE2E1-3 and UBE2N significantly increased the number of PML-positive cells post-infection, in an ICP0-dependent manner, indicating that ICP0 can use multiple E2s to degrade PML [57], [58].

One of the multi-protein complexes affecting HSV-1 replication was the Mediator complex, a large (>30 subunits) complex which links specific transcription factors to the RNA polymerase II transcription machinery [40]. As the requirement of Mediator subunits in the replication of herpes and other viruses is already well-known [7], [9], [11], [41], it was striking that depletion of the Med23 subunit exerted the opposite phenotype and led to a strong increase in virus growth. The Mediator is composed of four distinct modules termed the head, middle, tail and kinase domains, which provide the Mediator with some degree of active control over transcription [59]. As individual subunits of this large complex interact with and exert functional effects via specific transcription factors, it is not unexpected that the observed anti-viral effects were specific to Med23 [60]. Within the Mediator, Med23 forms a tight sub-complex with Med24 and Med16 [60], [61]. The increase in virus replication observed upon depletion of Med24 may be caused by the destabilisation of the structure of this sub-complex (**Figure S6b**).

Investigations into the mechanism of action revealed Med23 inhibits HSV-1 replication by preferentially inducing a type III interferon response (IFN-λ) at the mRNA and protein level. This induction was mediated via a direct interaction with the transcription factor IRF7, which resulted in a synergistic increase in IFN-λ expression. Med23 was unable, however, to further enhance IRF7-induced levels of IFN-β, suggesting an additional level of complexity to the regulation of interferon signalling. Interestingly, the inhibitory effect of Med23 was specific to HSV-1, with replication of a range of other viruses including Vaccinia virus and Semliki Forest Virus being unaffected by Med23 depletion. As Vaccinia virus is resistant to IFN-λ anti-viral activity [62], this observation further highlights the importance of IFN-λ, as opposed to IFN-β, in the anti-viral effect of Med23. The R617Q mutation in Med23 (R611Q in Med23 transcript variant 1, used here) was unable to enhance IRF7-induced IFN-λ expression. This mutation causes hereditary dementia [63], and the failure to induce IFN-λ and thereby control HSV-1 in the brain may be a potential cofactor for the development of dementia, similar to Alzheimer's disease [64].

There is mounting evidence for a role of the IFN-λ family in the regulation of virus pathogenesis [45], particularly in the case of Hepatitis C infection where a polymorphism in the promoter region of IFN-λ3 (IL-28B; polymorphism rs12979860), which correlates with plasma levels of IFN-λ3 [50], is associated with disease and treatment outcome [49]. Individuals with recurrent HSV-1 reactivation have been shown to be deficient in IFN-λ expression [51], and here we found the similar association between the IFN-λ3 promoter polymorphism and ethnically Italian patients suffering recurrent and severe reactivations of HSV-1-related oral herpes outbreaks, albeit with a small sample group (*n* = 58). Furthermore, sporadic mutations and genetic polymorphisms in innate immune receptor and signalling molecules that lead to the induction of type I and III IFNs have also been shown to be associated with Herpes Encephalitis [65], as well as oral and genital Herpes [66], [67].

HSV infection controlled by a complex, interconnected and highly regulated network of cytokines expressed by innate immune cells. Type I IFNs mainly produced by HSV-infected keratinocytes [68] and pDCs [69] inhibit the spread from neurons to epithelial cells and between epithelial cells [70], similar to IFN-γ. Type III IFNs are also able to directly inhibit HSV-1 infection in primary neurons, astrocytes, macrophages and dendritic cells [71], [72]. IFN-γ levels produced by peripheral blood CD4+ T-cells correlate with the frequency of HSV-1 reactivation [73]. IFN-λ is able to induce expression of both itself and the type I IFNs, and a similar effect has also been observed for type I IFNs which induce both type I and III IFNs [71], [72]. Type III IFNs are mainly expressed by myeloid dendritic cells (mDC) and monocyte-derived macrophages [74], and signal through the heterodimeric IL10RB/IL28RA receptor complex whose expression is largely restricted to cells of epithelial origin and plasmacytoid dendritic cells (pDC), in contrast to the broadly expressed type I IFN receptor (IFN-αR1/2) [75], [76]. Since primary HSV-1 infection and reactivation affects skin and mucosa in the majority of cases, IFN-λ may play a much greater role in the control of HSV-1 pathogenesis, likely in a complex network of coregulated type I and II IFNS, than previously thought. We hypothesize that HSV-infected DCs at the site of the lesion (such as skin Langerhans DCs whose role in IFN-λ production is currently unknown, or intruding myeloid DCs) in individuals with the rs12979860 T/T or C/T haplotype express reduced levels of type III IFNs, and, in consequence, of type I IFNs, which leads to a reduced inhibition of local HSV-1 replication and the occurrence of fresh skin lesions. However, the relative contribution of IFN-λ1 and λ2/3 to the interferon-mediated control of HSV-1 replication *in vivo*, and indeed the role of Med23 in this, remains to be seen.

In summary, this study provides a comprehensive and robust analysis of HFs that influence HSV-1 replication *in vitro*, which will benefit many future studies on HSV-1. The identification of Med23 as a crucial cellular component for IFN-λ expression, and evidence for the significant role of type III IFN in the innate immune control of HSV-1 *in vitro* and *in vivo*, demonstrates the power of combined, genome-scale studies to identify physiologically important HFs for virus pathogenesis. Future studies will clarify the role of genetic variations in both Med23 and IFN-λ in HSV-1-related diseases, such as meningitis, keratitis and orolabial/genital reactivations.

## Materials and Methods

### siRNA screen

siRNA SMARTpools (4 siRNAs per gene) at 0.3 µM were dispensed in 10 µl volumes using a Rapidplate384 liquid handler (Qiagen) into triplicate black 384-well plates (Corning), sealed with adhesive seals (ThermoFisher) and plastic lids. Plates were stored at −80°C until needed (minimum 24 h, maximum 48 h). On the day of transfection, assay plates were thawed at room temperature and 10 µl transfection reagent (Dharmafect 1, Dharmacon), diluted in Hank's buffered saline solution (HBSS, ThermoFisher) to give a final concentration of 0.1%, was added using a Multidrop 384 (ThermoFisher). Plates were incubated for 20 min at room temperature to allow formation of transfection complexes. During complex formation, low-passage (p20–22) Hela cells (ECACC) from ∼50% confluent flasks were washed in PBS and trypsinised in Trypsin-EDTA (Lonza) before diluting in phenol red-free, antibiotic-free transfection medium (DMEM/F-12 1∶1/5% FCS with 15 mM Hepes and L-glu; Gibco). Cells were counted and 3×10^3^ cells in 40 µl were added to each well using the Multidrop 384. Plates were incubated for 48 h at 37°C in a humidified incubator with 5% CO_2_. To infect, media was removed from plates by inversion, and 10 µl media (as for transfection, but containing penicillin-streptomycin; Lonza) or virus (HSV-1-eGFP strain C12, diluted to MOI 0.5 in infection media) [34] was added using the Multidrop 384. Plates were incubated at 37°C for 1 h before 50 µl infection media was added and plates returned to the incubator. Replication was monitored as a function of eGFP fluorescence from 24 h to 80 h post-infection using the POLARstar OPTIMA plate reader (BMG Labtech). Virus replication slopes over the linear phase were calculated and normalized to mock transfected wells on individual assay plates, and the mean replication slope from six replicates used for subsequent data analyses.

### Cell viability assay

Cells were transfected as described above, and the cytotoxicity of siRNAs was determined using the CellTiter Blue (CTB, Promega) reagent, which gives a fluorescent or absorbance signal relative to the number of live cells. Briefly, 5 µl CTB was added per well using the Multidrop 384. Plates were incubated at 37°C in a humidified incubator with 5% CO_2_ for 2 h before measuring fluorescence (POLARstar OPTIMA plate reader). Readings were normalized to viability of mock-transfected cells, per plate, and mean cell viability over three replicates was calculated. Distribution analysis of cell viability values identified median viability as 60%, and values <60% were considered cytotoxic.

### Yeast two-hybrid protein interaction screen

The HSV-1 clone collection was cloned by recombinatorial (GATEWAY™, Invitrogen) and conventional cloning into the bait vector pGBKT7, and screened against a library pooled from 12,381 MGC clones [35] in the pGADT7 prey vector using a semi-automated Y2H assay [77]. Interacting prey cDNAs were identified by sequential blasting of RefSeq, ENSEMBL and Unigene databases. BLAST hits with identical parameters (score, expectation value, length of alignment) were considered indistinguishable and counted separately. A high-confidence dataset was generated from interaction pairs isolated at least twice, or where the bait interacted with two highly related, non-promiscuous preys.

### Bioinformatic analyses

Interactions between HSV-1 and human proteins were connected to a network of human protein-protein interactions (a total of 62,310) taken from the databases HPRD [78](Release 9), BioGRID [79], DIP [80], MINT [30] and IntAct (downloaded May 18^th^ 2010). A high-confidence interaction set (9,829 interactions) was compiled from interactions identified in at least two studies. Betweenness centrality (*g(v)* of a protein v was calculated as *g(v)* = ∑*s_‡v‡t_(σ_st_(v)/σ_st_)*, where *σ_st_* is the total number of shortest paths from protein *s* to protein *t*, and *σ_st_(v)* is the number of those shortest paths that contain *v*. Betweenness centrality was normalized by dividing by the total number of protein pairs in the network. Enrichment for functional annotations from gene ontology (GO) [81], KEGG [82], [83], REACTOME [84], [85], and BIOCARTA was performed using DAVID [86]. Data on known human protein complexes was retrieved from the CORUM database, and complexes with subunits showing consistently stronger effects (inhibiting or enhancing) than expected by chance were detected using Wilcoxon's rank-sum test. Genes included in the RNAi screen were ranked by their distance from the median knockdown, with the most inhibiting and enhancing genes being ranked highest. FDR was used for multiple testing correction.

### siRNA SMARTpool deconvolution for HSV-1 replication assay validation

The HSV-1 replication phenotype observed in the primary screen was validated for a subset of candidates by deconvoluting the assay SMARTpools. The four individual siRNAs targeting different regions of each gene, as well as a reconstituted SMARTpool, were diluted to 0.3 µM in 1× siRNA buffer and dispensed to black 384-well plates. Transfection and infection was carried out as described above. Replication slopes were calculated and normalized as described, and a phenotype was considered validated if two or more of the four siRNAs resulted in the same, or better, phenotype.

### Determination of virus specificity

For inter-viral comparison, siRNAs were considered inhibitory or enhancing if normalized replication was ±2×STDEV of the controls

#### a) HSV-1 replication assays

Selected siRNA SMARTpools were diluted to 0.3 µM in 1× siRNA buffer and dispensed in black 96-well plates (Corning). To this 10 µl Dharmafect 1, diluted in HBSS to a final concentration of 0.15%, was added using the Multidrop 384. Following a 20 min incubation to enable complex formation, 1×10^4^ Hela cells in 80 µl transfection media were seeded on to the complexes bringing the final volume of transfection to 100 µl in each well. Plates were incubated for 48 h at 37°C in a humidified incubator with 5% CO_2_ before infection. To infect, media was removed from plates by inversion, and 25 µl media (as for transfection, but containing penicillin-streptomycin; Lonza) or virus (strain C12, diluted to MOI 0.5 in infection media) was added using the Multidrop 384. Plates were incubated at 37°C for 1 h before virus was removed by plate inversion and 100 µl infection media was added. Plates were returned to the incubator before replication was monitored as a function of eGFP fluorescence from 24 h to 80 h post-infection. Virus replication slopes over the linear phase were calculated and normalized to mock transfected wells on individual assay plates, and the mean replication slope from six replicates used for subsequent data analyses.

#### b) VZV replication assays

Selected siRNA SMARTpools were diluted to 0.3 µM in 1× siRNA buffer and dispensed in black 384-well plates (Corning). To this, 10 µl Dharmafect 1 diluted in HBSS to a final concentration of 0.07% was added using the Multidrop 384. Following a 20 min incubation to enable complex formation, 2.5×10^3^ MeWo cells (ATCC, HTB-65™) in 40 µl media (EMEM/10% FCS/1% non-essential amino acids) were seeded on to the complexes bringing the final volume of transfection to 60 µl in each well. Plates were incubated for 48 h at 37°C in a humidified incubator with 5% CO_2_ before infection. To infect, media was removed from plates by inversion and ∼25 colony forming units of VZV-eGFP-infected MeWo cells (vaccine strain Oka) [87] diluted in MeWo growth media were seeded on to the complexes using the Multidrop 384. Virus growth was measured in 3 h intervals as a function of eGFP fluorescence from 44 to 72 h post-infection. Virus replication slopes over the linear phase were calculated and normalized to mock transfected wells on individual assay plates, and the mean replication slope from six replicates used for subsequent data analyses.

#### c) hCMV replication assays

Selected siRNA SMARTpools were diluted to 0.5 µM in 1× siRNA buffer and dispensed in 10 µl volumes in black 384-well plates (Corning). To this, 10 µl Dharmafect, 1 diluted in HBSS to a final concentration of 0.2%, was added using the Multidrop 384. Following a 20 min incubation to enable complex formation, 3×10^3^ MRC-5 (ATCC, CCL-171™) in 40 µl growth medium (phenol red-free DMEM/10%FBS/L-glutamine/1% non-essential amino acids were seeded on to the complexes bringing the final volume of transfection to 60 µl in each well. Plates were incubated for 48 h at 37°C in a humidified incubator with 5% CO_2_ before infection. To infect, media was removed from plates by inversion, and 10 µl media (as for transfection, but containing penicillin-streptomycin) or virus hCMV-GFP (strain AD169) [88], diluted to MOI 0.5 in infection media, was added using the Multidrop 384. Plates were incubated at 37°C for 1 h before 50 µl infection media was added and plates returned to the incubator prior to monitoring virus replication. Replication was monitored as a function of eGFP fluorescence from 24 h to 80 h post-infection using the POLARstar OPTIMA plate reader (BMG Labtech). Virus replication slopes over the linear phase were calculated and normalized to mock transfected wells on individual assay plates, and the mean replication slope from six replicates used for subsequent data analyses.

#### d) SFV replication assays

Selected siRNA SMARTpools were diluted to 0.5 µM in 1× siRNA buffer and dispensed in 10 µl volumes in black 96-well plates (Corning). To this, 10 µl Dharmafect 1 diluted in HBSS to a final concentration of 0.15% was manually added. Following a 20 min incubation to enable complex formation, 4×10^4^ Hela cells in 80 µl transfection media (DMEM/5% FCS/L-glu) were seeded on to the complexes bringing the final volume of transfection to 100 µl in each well. Plates were incubated for 48 h at 37°C in a humidified incubator with 5% CO_2_ before infection. To infect, media was removed from plates by inversion, and 25 µl media (as for transfection, but containing penicillin-streptomycin; Lonza) or virus (SFV4(3H)-Rluc [89] diluted in phosphate buffered saline (PBS)/0.75% bovine serum albumin to an MOI 0.01) was manually added. Plates were incubated at 37°C for 1 h before media (as for transfection media) was added manually to increase the volume to 100 µl per well. Plates were incubated at 37°C for 24 h before cells were lysed using a passive lysis buffer and *Renilla* luciferase levels measured with a microplate reader (Promega) using a dual luciferase reporter assay kit (Promega). Luciferase activity, which is representative of virus genome replication, was normalized to mock-transfected cells and mean luciferase activity from six replicates used for subsequent data analyses.

#### e) Vaccinia virus replication assays

Hela cells were transfected as described in primary siRNA screen. Plates were incubated for 48 h at 37°C in a humidified incubator with 5% CO_2_ before infection. To infect, media was removed from plates by inversion, and 15 µl media (as for transfection, but containing penicillin-streptomycin) or 15 µl media containing Vaccinia virus strain WR with eGFP-tagged A5 protein [90], diluted to MOI 0.05, was added using the Multidrop 384. Plates were incubated at 37°C for 1 h before 50 µl of media was added to each well, the plates inverted to remove the media and virus, and a final volume of 50 µl of media added to the plates before they were returned to the incubator. Replication was calculated as a function of eGFP fluorescence at 48 h post-infection using the POLARstar OPTIMA plate reader (BMG Labtech). Virus replication was normalized to mock transfected wells on individual assay plates, and the mean replication from eight replicates used for subsequent data analyses.

### qPCR for siRNA knockdown

Hela cells were transfected with selected SMARTpool siRNAs in 96-well plates, in triplicate, as described. After 48 h transfection, medium was removed, cells rinsed in PBS and lysed in 100 µl TRIZOL (Invitrogen). Triplicate wells were combined, and RNA extracted by standard phenol:chloroform extraction methods. mRNA levels were determined by TaqMan qPCR, using the one-step RT-qPCR kit (Thermofisher), with gene-specific primers (**Table S9 in Text S2**), and probes from the Universal Probe Library (Roche). Expression levels normalized to the housekeeping cellular gene hypoxanthine phosphoribosyltransferase 1 (HPRT) and calibrated to mock-transfected cells. qPCR was carried out in duplicate for each sample, and the mean of normalized expression levels calculated.

### LUMIER pull-down assay for validating Y2H protein interactions

Proteins were transiently expressed in HEK293 cells as hybrid proteins with the *Staphylococcus aureus* protein A tag or *Renilla reniformis* luciferase fused to their amino termini. 20 ng of each expression construct were transfected into 1×10^4^ HEK293 cells using 0.05 µl of lipofectamine 2000 (Invitrogen) in 96-well plates. After 40 h, medium was removed and cells were lysed on ice in 10 µl of ice-cold lysis buffer (20 mM Tris pH 7.5, 250 mM NaCl, 1% TritonX-100, 10 mM EDTA, 10 mM DTT, Protease Inhibitor Cocktail (Roche), Phosphatase Inhibitor Cocktail (Roche), Benzonase (Novagen) 25 units per µl final concentration) containing sheep-anti-rabbit IgG-coated magnetic beads (Invitrogen, Dynabeads M280, 2 mg/ml final concentration). Lysates were incubated on ice for 15 minutes. 100 µl of wash buffer (PBS, 1 mM DTT) were added per well, and 10% of the diluted lysate was removed to determine the luciferase activity present in each sample before washing. The remaining sample was washed 6 times in wash buffer in a Tecan Hydroflex plate washer. Luciferase activity was measured in the lysate as well as in washed beads. Negative controls were wells transfected with the plasmid expressing the luciferase fusion protein and a vector expressing two copies of protein A. For each sample, four values were measured: the luciferase present in 10% of the sample before washing (“input”), the luciferase activity present on the beads after washing (“bound”), and the same values for the negative controls (“input nc”, and “bound nc”). Normalized interaction signals were calculated as follows: Log(bound)/log(input) – log(bound nc)/log(input nc). Normalized interaction signals were z-transformed by subtracting the mean and dividing by the standard deviation. The mean and standard deviation were calculated from large datasets of protein pairs which were not expected to interact, i.e. from negative reference sets.

### HSV-1 microscopy

Selected siRNAs SMARTpools were diluted to 500 nM in HBSS and 40 µl was incubated with 40 µl Dharmafect 1 diluted in HBSS to a final concentration of 0.15%. After 20 min incubation, 3×10^4^ Hela cells in 320 µl transfection medium were added, mixed with the transfection complexes and transferred to 8-well glass bottomed chamber slides (Becton Dickinson). Plates were incubated for 48 h at 37°C in a humidified incubator with 5% CO_2_ before infection by removing medium and adding 100 µl HSV-1-eGFP at a MOI of 1. After incubation for 1 h at 37°C, virus was removed and replaced with 500 µl growth medium. Images were acquired 48 h post-infection.

### Flow cytometry of HSV-1-eGFP infected cells

Select siRNAs SMARTpools were diluted to 500 nM in HBSS and 100 µl was incubated with 100 µl Dharmafect 1 diluted in HBSS to a final concentration of 0.15% in individual wells of a 12-well plate. After 20 min incubation, 2×10^5^ Hela cells in 800 µl transfection medium were added. Plates were incubated for 48 h at 37°C in a humidified incubator with 5% CO_2_ before infection by removing medium and adding 500 µl HSV-1-eGFP at a MOI of 1. After incubation for 1 h at 37°C, virus was removed and replaced with 2 ml growth medium. After 48 h, medium was removed, cells rinsed in PBS and dislodged by trypsinisation. Cells were washed in PBS and pelleted by centrifugation for 10 min at 199 *g*. Supernatant was removed and cells fixed in 4% paraformaldehyde before analysing for eGFP expression by flow cytometry (FACS DiVa, BD Biosciences) using the CellQuest software package.

### Transient and stable over-expression of Med23

For transient over-expression, 1.5×10^4^ HEK293 cells were seeded in black 96-well plates. The following day, cells were transfected with 100 ng pCR3-Med23 using Lipofectamine™ LTX (Invitrogen) and incubated for 48 h before infection with the recombinant HSV-1 reporter viruses C12 and VP26-YFP at MOI 0.5. Replication growth curves were monitored, and endpoint replication (as determined by fluorescence) was normalized to untransfected cells. For stable expression, Hela cells were transduced with pLenti-Med23, generated using the ViraPower™ Lentiviral Expression System (Invitrogen), as per manufacturers' instructions. Stable cells were infected, and replication monitored, as above. For confirmation of overexpression, RNA was extracted (QIAGEN RNA-easy kit) and expression quantified by one-step RT-qPCR (Thermofisher), with gene-specific primers (Table S9 in Text S2), and probes from the Universal Probe Library (Roche). Expression levels were normalized to the housekeeping cellular gene hypoxanthine phosphoribosyltransferase 1 (HPRT) and calibrated to mock-transfected cells. qPCR was carried out in duplicate for each sample, and normalized expression levels averaged. The VP26-YFP reporter virus was generated from a KOS BAC kindly provided by David Leib using the Red-mediated recombination system, where the first 4 amino acids of VP26 were replaced with YFP [91], [92], [93].

### Effect of Med23 in Stat1-deficient A549-V cells

The effect of Med23 over-expression and depletion was investigated in interferon-deficient cells. The human alveolar epithelial cell line A549, and A549-V, a Stat1-deficient derivative cell line stably expressing the V protein from Simian virus 5 [43], were seeded at 2×10^4^ cells per well in a 96-well plate, and transfected with Med23 siRNA or pCR3-Med23 and infected as described for Hela cells.

### qRT-PCR analysis of interferon and cytokine induction

A549 cells were transfected with 100 ng pCR3 or Med23 overexpression plasmids, or RSCF or Med23 SMARTpool siRNA (50 nM) in duplicates, in 96-well plates as described. RNA was harvested 20 h post-transfection, and mRNA expression levels quantified by qRT-PCR, as described.

### IFN-λ ELISA

Induction of IFN-λ by Med23 was determined in a range of cell types by seeding cells in 96-well plates to be ∼80% confluent the next day. Cells were transfected in duplicates with 100 ng pCR3 or pCR3-Med23 using Lipofectamine LTX with Plus reagent (Invitrogen), in antibiotic-free medium. IFN-λ levels quantified 96–120 h post-transfection. The synergistic effect of Med23 and IRFs on IFN-λ induction was determined by co-transfection of pCR3 or pCR3-Med23 (50 ng) with pCR3-IRF7 (50 ng) in A549 cells, with Lipofectamine LTX with Plus reagent. The effect of Med23 depletion on IFN-λ induction was determined by transfection of A549 cells in 96-well plates with 50 nM RSCF or Med23 siRNA. IFN-λ was quantified 120 h post-transfection. IFN-λ protein expression was quantified in supernatants using a commercial IFN-λ DuoSet ELISA kit (R and D Systems).

### IFN-λ1 luciferase reporter assay

A point mutation (R611Q) was introduced into Med23 (Transcript Variant 1) by PCR with specific primers (see Table S9 in Text S2) and the clone verified by sequence analysis. A549 cells were co-transfected with 60 ng of pCR3, pCR3-Med23 or pCR3-R611Q, 60 ng of pCR3-IRF7, 20 ng of IFN-β-, IFN-λ1- or ISRE-responsive luciferase reporter constructs and 10 ng pRL-TK, a Renilla Luciferase transfection control, in antibiotic-free low serum (1%) medium. After 33 h cells were lysed, and firefly (promoter reporter construct) and Renilla (transfection control) luciferase activity was measured (Dual-Luciferase Reporter Kit, Promega). Relative luminescence activity was normalized to Renilla (as a transfection control).

### Interferon stimulation assays

A549 cells were transfected in triplicate in black 96-well plates, as described. After incubation at 37°C for 24 h, cells were serum-starved for 24 h by growing in low serum (1%) medium, before cells were left untreated or stimulated with 50 ng/ml IFN-α, IFN-β or IFN-γ, or 100 ng/ml IFN-λ1 or IFN-λ2/3 in serum-reduced medium. After 6 h cells were infected with HSV-1 C12 diluted to MOI 0.5 in IFN-containing serum-reduced medium. After 1 h incubation, virus was removed and media replaced with 100 µl serum-reduced growth medium containing no interferon, IFN-α/-β/-γ at 50 ng/ml, or IFN-λ1/-λ2/3 at 100 ng/ml. Replication was monitored as described and normalized to replication in mock-transfected, unstimulated cells.

### Med23 interaction with IRF7

Potential interactions between Med23 and the IRFs were determined by yeast two-hybrid analysis and confirmed by co-immunoprecipitation (Co-IP) in mammalian cells.

#### a) Co-immunoprecipitation

Co-immunoprecipitation was performed using the epitope-tagged plasmids pGBKT7 (Myc epitope) and pGADT7 (HA epitope) containing the T7 promoter, and recombinant vaccinia virus vTF-7 expressing the T7 RNA polymerase (NIH AIDS repository). 5×10^6^ HEK293 cells were seeded in 10 cm dishes and the following day infected with vTF-7 (MOI 10) for 1 h before transfecting 10 µg each of empty bait (pGBKT7) or Med23-Bait, and IRF-Prey (pGADT7) vectors with Lipofectamine (Invitrogen). After 48 h cells were lysed on ice for 30 min in NP40 buffer, containing protease and phosphatase inhibitors. Debris was removed by centrifugation, and protein quantified with a BCA protein assay kit (Thermo scientific, UK) as per manufacturers' instructions. Equal protein quantities (650 µg per sample) were pre-cleared by continuous mixing with 50 µl pre-equilibrated Protein G Sepharose beads for 1 h at 4°C. Samples were centrifuged and supernatants halved before overnight mixing incubation with beads pre-coated with 1 µg α-HA (Roche, UK) or α-c-Myc (Santa Cruz, UK) at 4°C. Beads with precipitated proteins were washed three times with ice-cold NP40 buffer, resuspended in 2× SDS protein sample buffer and boiled for 10 min before SDS-PAGE separation on two 8% polyacrylamide gels. Proteins were transferred onto nitrocellulose membranes overnight at 4°C before blocking with 5% milk in TBS-Tween then incubating with either α-HA or α-myc antibody (diluted 1∶1000 in 5% milk/TBS-Tween). Membranes were washed in TBS-Tween before incubation with HRP-conjugated secondary antibody (diluted 1∶3000 in 5% milk/TBS-Tween). After further washes, proteins were detected by incubation with ECL Western blotting detection system, exposing to X-ray film and developing films in an OPTIMAX X-Ray film processor.

#### b) Yeast two-hybrid assays

Haploid yeast strains AH109 and Y187 were transformed with 1 µg prey (pGADT7 or pGADT7-IRF1, -IRF2, -IRF3, -IRF4, -IRF5, -IRF7 or -IRF9) or bait (pGBKT7 or pGBKT7-Med23) plasmid DNA, respectively, and grown overnight in synthetic defined (SD) medium lacking either leucine (-L; Prey) or tryptophan (-W; Bait) before prey- and bait-expressing haploid yeast cells were mated overnight in SD-LW/5% YPDA medium. Haploids were selected in SD-LW for 48 h before transferring to triple-knockout, histidine-deficient SD-LWH liquid medium containing 3-aminotriazol (3-AT) and 4-methylumbelliferyl-β-D-galactopyranoside (4-MuX) for 5–7 days. Interactions were tested in quadruplicates, detected by growth of colonies on SD-LWH agar with 3-AT, and quantified by measurement of fluorescence released from α-galactosidase cleavage of 4-MuX upon protein interaction. Relative fluorescence (RFU) was normalized to negative control interaction (empty pGADT7 mated with empty pGBKT7).

### Genotypic analysis of the IFN-λ3 promoter

Ethnically Italian subjects with or without a history of recurrent Herpes labialis (HL) gave written voluntary informed consent and were enrolled in this study at the University of Rome Tor Vergata with the approval of the Ethical Committee at the University of Rome Tor Vergata. All subjects were interviewed by medically trained investigators using an appropriate questionnaire and agreed to provide saliva and/or blood samples. Data and blood sample collection was carried out as previously described [51]. A total of 58 healthy immunocompetent individuals (17 men and 41 women) age 22–61 years (overall median age 38.5 yr; male median age 40 yr, range 22–61; female median age 38, range 23–60) participated in the study. None of the patients presented with an active lesion at the time of or in the 3 weeks preceding saliva sample collection. For the purpose of this study, patients were characterized into no recurrence of HL (NR), low recurrence (L; 1–3 HL episodes/yr, with a maximum extension of 1 cm, mild symptoms and healing time <7 days), high recurrence (H; 4 or more HL episodes/yr, extension of lesions more than 1 cm), very high recurrence (H+; more than 4 HL episodes/yr, extension of lesions >3 cm and/or involving nose or cheek beyond the lip, more severe and long lasting associated symptoms including itch, burning, paresthesias and/or neuralgia, with healing times >7 days and who required antiviral therapy). The NR group consisted of 21 individuals (16 women [median age (range) 36 (27–520] and 5 men [median age (range) 39 (26–49)]. The L consisted of 18 individuals (12 women [median age (range) 37 years (23–60)] and 6 men [median age (range) 43 years (22–61)]). The H group consisted of 9 individuals (7 women [median age (range) 42 years (31–54)] and 2 men [median age (range) 44.5 years (37–52)]). The H+ group consisted of 8 individuals (5 women [median age (range) 38 years (28–55)] and 3 men [median age (range) 35 years (35–46)]). Saliva samples (∼3 ml) were obtained from each subject after an overnight fast and after rinsing the mouth twice with water, split into two aliquots and frozen at −20°C. Samples were anonymised and stored with dual code labels before shipping on dry ice to the University of Edinburgh for DNA extraction. Saliva and PBMC samples were thawed and DNA extracted using a QIAamp DNA Blood Mini Kit (Qiagen) as per manufacturer's instructions, quantified using a NanoDrop and IL28B genotype determined by melt-curve analysis PCR on a LightCycler480 (Roche) using the LightMix® Kit IL28B (TIB Molbiol) as per manufacturer's instructions. Significance of genotype association was determined by Fisher's exact test, comparing the frequency of the CC, CT or TT genotype in the NR group versus the L (p-value = 1), H (p-value = 0.12) or H+ (p-value = 0.015) clinical groups.

Below lists the GeneID numbers for genes and proteins mentioned within the text of this manuscript: IFITM1 (8519); IFN-α (cluster; 3438); IFN-β (3456); IFN-γ (3458); IFN-λ1 (282618); IFN-λ2 (282616); IFN-λ3(282617); IL10RB (3588); IL28RA (163702); IRF4 (3662); IRF7 (3665); IRF9 (10379); MED4 (29079); MED6 (10001); MED7 (9443); MED8 (112950); MED14 (9282); MED16 (10025); MED17 (9440); MED21 (9412); MED23 (9439); MED25 (81857); MED26 (9441); MED27 (9442); MED28 (80306); MED29 (55588); NR3C2 (4306).

## Supporting Information

Text S1 **Text for supporting figures.** Descriptive text for Figures S4 and S5, additional methods for Figures S4 and S5, and appropriate references.
(DOCX)

Text S2 **Supporting tables.** Table S1 – 358 Top 2.5% inhibitory and enhancing genes. Table S2 – Overlap of primary hit list with RNAi screens in other viruses. Table S3 – HFs identified by Y2H. Table S4 – Known interactors of HSV-1. Table S5 – Overlap between HSV-1 siRNA and Y2H screens. Table S6 –Validation of primary screen phenotypes by siRNA SMARTpool deconvolution and quantitative RT-PCR. Table S7 – Specificity of identified HFs to HSV-1 replication. Table S8 – Functional and pathway analysis of siRNA HFs. Table S9 – Primers and probes for qPCR assays.
(DOCX)

Figure S1 **Pathogen-host interactome analysis of RNAi and Y2H HFs.** (a) Combined HSV-1-human interactomes with intraviral, virus-host and high-confidence host-host interactions. Green, viral proteins; Pink, direct (level 1) interactors (Y2H screen); Blue, level 1 interactors (Y2H/RNAi screens); Cyan, host protein-protein (level 2) interactors (Y2H screen); Yellow, Level 2 interactors (Y2H/RNAi screens). Due to the large size of the human network, and in order to discern viral proteins, only the first two levels were plotted (inset: all levels). Combined HSV-1-human interactome. Inset, all levels. (b) Intraviral and virus-host degree (# interactions) distribution of HSV-1 proteins. (c) Correlation between intraviral and virus-host degrees in HSV-1. (d) Degree comparison of cellular interactors versus all proteins in human networks, where the degree indicates the number of interactions a particular protein has. (e) Betweenness centrality comparison of cellular interactors versus all proteins in human networks, where betweenness indicates the number of shortest paths between a protein pair, passing through the protein of interest. Statistically significant differences between viral targets and remaining proteins are denoted by *. (f) Distribution of HFs in the virus-host interactomes. Level 1 proteins are direct interactors with HSV-1 proteins, level 2 their interactors and so on. (g) Validation of HSV-1-host Y2H interactors. A subset of protein interactions identified in the HSV-1-host Y2H screen were validated using the LUMIER pull-down assay in a mammalian cell system. Strength of interaction was determined by Z-score, where a score 1 to 2 represents a weak interaction and score >2 represents a strong interaction. (h) Distribution of direct HSV-1 targets in the RNAi screen. The proteins directly targeted by HSV-1 were taken from the Y2H data set and from the literature curation. An enrichment of literature-derived targets could be observed in the top 5% most inhibiting knockdowns (2.4-fold enrichment; *p* = 0.008), but the same could not be observed for the Y2H-detected direct targets.
(TIF)

Figure S2 **Validation of HF identified by RNAi.** HFs identified in the HSV-1 perturbation screen were validated with deconvoluted siRNAs and qPCR: (a) chromoboxes, (b) homeoboxes, (c) general transcription factors, (d) nuclear receptors, (e) proteasome family members, (f) topoisomerases, (g) mediator complex subunits, (h) proteins involved in vesicle transport, (i) integrins, (j) Y2H interactors, (k) ubiquitin E2 ligases, (l) vesicle transport – further candidates, (m) interferon-stimulated membrane proteins and (n) others. HSV replication is presented as normalized replication slope, and is the mean of six individual assay points. Error bars represent standard deviation of the six data points. Deconvoluted siRNAs which had a sequence different to that in the original screen are highlighted in red. Genes for which the primary screen phenotype was not confirmed in the deconvolution assay are shown in bold text.
(TIF)

Figure S3 **HSV-1 HFs are involved in diverse cellular pathways and at multiple stages of the HSV life cycle.** Enrichment of protein functions among the HSV-1 HFs. The enrichment for gene ontology (GO) terms and KEGG, BIOCARTA or REACTOME pathway annotations among the HSV-1 HFs identified by (a) RNAi and (b) Y2H assay was performed using DAVID bioinformatics software. (c) Direct interactions between human and HSV-1 proteins. The protein-protein interactions (PPIs) depicted are from the high confidence Y2H data set and from literature curation. Circles and diamonds correspond to human and HSV-1 proteins, respectively. Human proteins detected in the Y2H screen are drawn with red borders. Human genes that showed the strongest effects in the RNAi screen are colored yellow (extreme 10%) and orange (extreme 5%). (d) Highly interconnected regions in the human interaction network composed of HFs. Highly interconnected regions in the human interaction network composed of HFs. We assembled a human interaction network using data from the major PPI databases. A subnetwork consisting of HFs was then defined by limiting the network to the HFs detected in the Y2H screen, the RNAi (extreme 5%) screen, and the literature curation. Highly interconnected regions in the subnetwork were sought out using the MCODE algorithm. The top six scoring regions are shown. Proteins displayed in red correspond to HFs that are known only from the literature, and those in green are those that were detected in either of the screens performed in this study. The three boolean values beside each gene symbol represent whether the HF is present in the Y2H screen, the RNAi screen (extreme 5%), or the literature curated set, in that order. Dominant functional categories could be observed in each of the regions, including transcription (e.g., Mediator complex, RNA polymerase II and associated genes), translation initiation, splicing, and intracellular transport.
(TIF)

Figure S4 **HFs involved in viral entry and capsid transport.** (a) Diagrammatic summary of the role of dynein chains in HSV-1 infection. (b) Microtubule transport is required for HSV-1 infection. The role of dynein microtubule transport components in HSV-1 replication was analysed by comparing the replication slope of HSV-1-eGFP (C12)-infected cells depleted for a range of dynein chains from the primary siRNA perturbation screen. Error bars represent the mean of three independent experiments done in duplicate. (c) Depletion of dyneins inhibits virus particle release. The effect of dynein chain depletion on HSV-1 particle release was determined by quantifying virus titer in supernatants of Hela cells depleted of DYNC1H1 (heavy chain), DYNC1I2 (intermediate chain) and DYNC2LI1 (light intermediate chains) in a high multiplicity (MOI 5; HSV-1 KOS) growth assay. Titers were compared to control transfected cells (NT, non-targeting siRNA). (d) Depletion of dynein chains prevents immediate-early gene expression. Immediate-early (ICP0) and late (VP16) viral protein expression in cells depleted of DYNC1H1, DYNC1I2 or DYNC2LI1 was analysed and quantified by Western blot. Levels were compared to control transfected cells (NT, non-targeting siRNA). (e) Quantification of ICP0 and VP16 protein expression. Protein levels of ICP0 and VP16 in cells depleted of DYNC1H1, DYNC1I2 or DYNC2LI1 were quantified with an Odyssey Imager and normalized to protein levels in control transfected cells (Non-targeting siRNA).
(TIF)

Figure S5 **E2 ubiquitin conjugating enzymes in HSV-1 immune evasion.** (a) Depletion of E2 ubiquitin ligases inhibits PML degradation following HSV-1 infection. Hela cells mounted on coverslips were depleted for a range of E2 ubiquitin ligases for 24 h before infecting with HSV-1 17+. Cells were fixed and stained for ICP0 (green) and PML (red), analysed by confocal microscopy and PML-positive cells were counted (5 fields of view per coverslip) and expressed as a mean percentage of PML-positive cells remaining (3 independent experiments). Error bars represent the standard deviation over 3 independent experiments. (b) Inhibition of PML degradation by E2s is ICP0-dependent. Hela cells were seeded on coverslips, transfected as above and infected with an ICP0 RING-finger deletion mutant (FXE). Remaining PML-positive cells were quantified as above. (c) Immunofluorescence staining for PML bodies in cells transfected with control siRNA not incorporated into the RISC complex (RSCF) or cells depleted of the E2 ubiquitin conjugating enzymes E2D1 or E2L3. The arrows highlight cells that have wt PML levels remaining in them whilst containing wt ICP0.
(TIF)

Figure S6 **Med23 is an anti-viral component of the largely pro-viral multi-protein Mediator complex.** (a) Diagrammatic summary of the role of Mediator complex subunits in virus replication. Subunits are coloured according to whether HSV-1 replication was unchanged (grey), inhibited (top 5%, red; top 10%, orange) or enhanced upon gene knockdown (top 5%, light green; top 10%, dark green). Subunits not included are white; herpesvirus proteins reported to target Mediator subunits are violet; Mediator subunits detected in other viral RNAi screens are highlighted by coloured diamonds. (b) Mediator complex subunits influence HSV-1 replication. Individual subunits of the Mediator complex and associated proteins were depleted by siRNA knockdown and infected with HSV-1 C12 (MOI 0.5). Replication was monitored over multiple rounds and the slope of replication over the linear phase was calculated and normalized to controls (mock-transfected cells). Grey, no significant effect; red, strongly pro-viral; green, strongly anti-viral. Error bars represent the mean of six replicates. (c) Stable or transient overexpression of Med23. Med23 was stably (HEK) or transiently (Hela) overexpressed and Med23 mRNA levels quantified by RT-PCR. Expression was normalized to HPRT and calibrated to parental cells. Error bars represent the standard deviation of technical replicates.
(TIF)

Figure S7 **Med23 inhibits HSV-1 by directly interacting with IRF7 to induce a Type III, IFN-λ interferon response.** (a) Med23 specifically induces interferons. A549 cells were transfected with pCR3 or Med23 and induction of a panel of cytokines and interferons determined by qRT-PCR. Expression was normalized to HPRT and calibrated to pCR3-transfected cells. Error bars represent the standard deviation of technical replicates. (b) Med23 directly interacts with IRF7. Haploid yeast expressing Med23-bait or IRF-prey constructs were mated in nutrition-deficient Media containing 4-MuX. Interactions were detected by measurement of fluorescence released by α-galactosidase cleavage of 4-MuX upon protein interaction. Relative fluorescence (RFU) was normalized to the negative control (−, empty bait mated with empty prey constructs). Error bars represent the standard deviation of technical quadruplicates. +, known interactors Myc and Max. (c) Med23 and IRF7 synergistically induce IFN-λ secretion. A549 cells were transfected with pCR3, Med23, IRF7 or Med23 with IRF7 and supernatant was harvested after 96 h to measure IFN-λ3 levels (pg/ml) by ELISA. Error bars represent the standard deviation of biological replicates, and the chart is representative of multiple experiments. (d) Quantification of Med23 and IRF7 following overexpression. A549 cells were transfected with pCR3, Med23, IRF7 or Med23 with IRF7 and mRNA expression levels of Med23 or IRF7 quantified by qRT-PCR 96 h post-transfection. Error bars represent the standard deviation of biological replicates, and the chart is representative of multiple experiments.
(TIF)
