# Supplementary material for: A Systematic Analysis of Host Factors Reveals a Med23-Interferon-λ Regulatory Axis against Herpes Simplex Virus Type 1 Replication
Source: PLoS Pathog. 2013 Aug 8;9(8):e1003514. doi: 10.1371/journal.ppat.1003514 (PMC3738494; doi:10.1371/journal.ppat.1003514)
Supplement: Text S1 — Text for supporting figures. Descriptive text for Figures S4 and S5, additional methods for Figures S4 and S5, and appropriate references. (DOCX) [file ppat.1003514.s001.docx]

**Text S1**

***Combined screening approach identifies functional redundancy in HSV-1 capsid transport via the microtubule network***

After fusion of viral and cellular membranes, incoming viral capsids are known to be transported to nuclear pores via the microtubule organizing centre (MTOC). Transport is mediated by the capsid proteins VP26 (UL35) and UL46 binding to the dynein light chains DYNLT1 (Tctex1) and DYNLT3 (rp3) [[1](#_ENREF_1),[2](#_ENREF_2)], however studies showing microtubule transport of incoming viral capsids in the absence of VP26 suggests additional interactions may occur [[3](#_ENREF_3)]. Indeed, our Y2H screen confirmed the known UL35:DYNLT3 interaction and identified additional interactions between the ribonucleotide reductase subunit UL39 and the light chains DYNLL1 and DYNLL2, validated by co-immunoprecipitation (**Fig. S5a; Fig. S2f**). The essential role of the intermediate and heavy dynein chains (DYNC1I2, DYNC2LI1 and DYNC1H1) in HSV-1 replication was shown by a strong inhibition of virus growth (>90%) following siRNA-depletion (**Fig. S5b**). Removal of these dyneins not only significantly reduced production of infectious virus particles in single-cycle growth assays (**Fig. S5c**) but also reduced immediate-early viral gene expression (**Fig. S5d, e**), indicating a failure of capsids to reach the nucleus in the absence of the dynein motor (DYNC1H1) and cargo-binding intermediate chains (DYNC1I2, DYNC2LI1). Depletion of the individual light dynein chains (DYNLL1, DLC2, DYNLRB1, DYNLRB2 and DYNLT1), however, had only a moderate effect on virus replication (53 to 67% inhibition; **Fig. S5b**), suggesting several viral proteins are able to bind to several light chains, consistent with a model of functional redundancy in capsid:dynein complex interactions [[3](#_ENREF_3),[4](#_ENREF_4),[5](#_ENREF_5)].

***HSV-1 overcomes intrinsic anti-viral host defense mechanisms by exploiting multiple cellular E2 ubiquitin ligases***

The HSV-1 RING-finger ubiquitin ligase ICP0 is one of the first viral proteins expressed during the initial stages of infection. ICP0 localises to promyelocytic leukemia (PML) nuclear bodies (ND10 domains) where it induces a proteasome-dependent degradation of core components (including PML and its SUMO-modified isoforms) to block intrinsic anti-viral host defense [[6](#_ENREF_6),[7](#_ENREF_7)]. *In vitro* ICP0 is biochemically active as an E3 ubiquitin ligase in the presence of E2 ubiquitin-conjugating enzymes (E2s) UBE2D1 (UbcH5a) and UBE2E1 (UbcH6) [[8](#_ENREF_8)], but which E2s are used during infection has remained unclear. In this study, we found 20 cellular E2s to be essential for viral replication. Depletion of specific E2s, including UBE2D1-D4, UBE2E1-E3 and UBE2N, significantly affected ICP0s ability to degrade PML during infection (**Fig. S6, c**). This was ICP0-dependent, as the number of PML-positive cells following E2 depletion and subsequent infection with an ICP0 RING-finger deletion mutant was unaffected (**Fig. S6b, c**). Taken with other studies, these data suggest that HSV-1 ICP0 is promiscuous in its exploitation of E2s to Mediate PML degradation and ensure successful infection [[8](#_ENREF_8),[9](#_ENREF_9)].

**Methods for Supporting Figures**

***HSV-1 capsid transport***

*a) High multiplicity single cycle growth assay.*

Hela cells seeded in 6- or 24-well dishes were transfected with 100 nM siRNA using Dharmafect 1 following the manufacturer’s instructions (Dharmacon). Control siRNA was a non-targeting duplex (sequence AUUCUAUCACUAGCGUGAC; Dharmacon). At 48-60h post-transfection, cells were infected with HSV-1 strain KOS at a MOI of 5. The inoculum was removed after 1h and unabsorbed virus was inactivated by treatment with acid wash (40 mM citric acid, 135mM NaCl, 10 mM KCl, pH 3.0) for 1min at room temperature. Following 3 washes with PBS, cells were incubated in growth Media at 37˚C. After 18h, cells were collected by scraping into growth Media, sonicated, frozen at -80˚C and thawed to liberate infectious virus. Virus titers were determined by assaying ten-fold serial dilutions on Vero cells. Confluent monolayers of Vero cells were incubated with virus dilutions for 1h at 37°C, overlaid with 0.6% [vol/vol] carboxymethylcellulose in DMEM supplemented with 2% fetal calf serum, 100U/ml penicillin G, 0.1mg/ml streptomycin and 2mM glutamine, and incubated at 37°C for 2 days. Prior to plaque counting, cells were fixed with 3.7% [vol/vol] formaldehyde in PBS for 15-20min and stained with 0.1% [wt/vol] Toluidine blue for 15-20min.

*b) Protein expression analysis.*

For protein expression analysis, cells were transfected and infected as above, before cells were collected by scraping into growth Media at 3h (ICP0) or 6h (VP16) post-infection. Cells were pelleted by centrifugation at 4000 rpm for 5min in a benchtop microfuge. Cell pellets were resuspended in lysis buffer (50 mM Tris [pH 7.9], 150 mM NaCl, 1% NP-40, 1% sodium deoxycholate) supplemented with protease inhibitor cocktail (Roche) and incubated on ice for 20min. Lysates were clarified by centrifugation at 17,000 x g for 15min at 4°C in a benchtop microfuge. Protein samples were separated by SDS-PAGE, and transferred to nitrocellulose membranes by western blot. Membranes were probed with primary antibodies specific for ICP0 (1:1000, ab6513; Abcam), anti-alpha tubulin (1:2000, MCA77G; AbD Serotec) or anti-VP16 (1:10, LP1; from A. Minson, University of Cambridge) followed by anti-mouse-IRDye 800CW (Li-Cor Biosciences) and anti-rat-Alexa 680 (Invitrogen). Membranes were scanned using an Odyssey Imager and data quantified with the Odyssey Application software (Li-Cor Biosciences).

***Viral ubiquitination***

3 x 10^4^ HeLa cells mounted on 13mm coverslips were transfected with 60 nM SMART pool siRNAs using DharmaFECT-1 at a final concentration of 1.25% for 24h prior to infection with either wt HSV-1 (strain 17+) or ICP0 RING-finger deletion mutant (FXE) [[10](#_ENREF_10)] for 3h. The cells were subsequently fixed and permeabilised in buffer A (10mM Hepes pH 7.0, 100mM NaCl, 300mM sucrose, 3mM MgCl2, 5mM EGTA, 0.5% Triton-X-100) and stained for PML (Bethyl A301-167A) and ICP0 (11060) [[11](#_ENREF_11)] in buffer A containing 2% human pooled serum (CELLect MP) prior to mounting. Coverslips were examined using a Zeiss Axioplan LSM 510 confocal microscope with laser excitations at 488 and 633nm for ICP0 and PML respectively. The data from each channel was collected using independent multi-track laser excitation with the appropriate band pass filters to eliminate optical emission overlap. Data was collected with two-fold averaging at a resolution of 1,024 x 1,024 pixels using an optical slice of 1μm utilising a 63x objective oil immersion lens. Data sets were processed using the LSM 510 software and exported as TIF files for preparation in Photoshop. A minimum of five independent fields of view per coverslip were counted (typically 20-25 cells/field) for the number of PML positive cells and expressed as a percentage of PML positive cells remaining in the total cell population counted. Histograms represent the percentage mean average of PML positive cells remaining from three independent experiments; error bars represent the standard deviation in the total number of PML positive cells over three independent experiments.

**References**

1. Gennerich A, Vale RD (2009) Walking the walk: how kinesin and dynein coordinate their steps. Curr Opin Cell Biol 21: 59-67.

2. Douglas MW, Diefenbach RJ, Homa FL, Miranda-Saksena M, Rixon FJ, et al. (2004) Herpes simplex virus type 1 capsid protein VP26 interacts with dynein light chains RP3 and Tctex1 and plays a role in retrograde cellular transport. J Biol Chem 279: 28522-28530.

3. Dohner K, Radtke K, Schmidt S, Sodeik B (2006) Eclipse phase of herpes simplex virus type 1 infection: Efficient dynein-mediated capsid transport without the small capsid protein VP26. J Virol 80: 8211-8224.

4. Radtke K, Kieneke D, Wolfstein A, Michael K, Steffen W, et al. (2010) Plus- and minus-end directed microtubule motors bind simultaneously to herpes simplex virus capsids using different inner tegument structures. PLoS Pathog 6: e1000991.

5. Zaichick SV, Bohannon KP, Hughes A, Sollars PJ, Pickard GE, et al. (2013) The herpesvirus VP1/2 protein is an effector of dynein-mediated capsid transport and neuroinvasion. Cell Host Microbe 13: 193-203.

6. Everett RD, Freemont P, Saitoh H, Dasso M, Orr A, et al. (1998) The disruption of ND10 during herpes simplex virus infection correlates with the Vmw110- and proteasome-dependent loss of several PML isoforms. J Virol 72: 6581-6591.

7. Everett RD, Parada C, Gripon P, Sirma H, Orr A (2008) Replication of ICP0-null mutant herpes simplex virus type 1 is restricted by both PML and Sp100. J Virol 82: 2661-2672.

8. Boutell C, Sadis S, Everett RD (2002) Herpes simplex virus type 1 immediate-early protein ICP0 and is isolated RING finger domain act as ubiquitin E3 ligases in vitro. J Virol 76: 841-850.

9. Gu H, Roizman B (2003) The degradation of promyelocytic leukemia and Sp100 proteins by herpes simplex virus 1 is mediated by the ubiquitin-conjugating enzyme UbcH5a. Proc Natl Acad Sci U S A 100: 8963-8968.

10. Everett RD (1989) Construction and characterization of herpes simplex virus type 1 mutants with defined lesions in immediate early gene 1. J Gen Virol 70 ( Pt 5): 1185-1202.

11. Everett RD, Cross A, Orr A (1993) A truncated form of herpes simplex virus type 1 immediate-early protein Vmw110 is expressed in a cell type dependent manner. Virology 197: 751-756.
