# Supplementary material for: A Systematic Analysis of Host Factors Reveals a Med23-Interferon-λ Regulatory Axis against Herpes Simplex Virus Type 1 Replication
Source: PLoS Pathog. 2013 Aug 8;9(8):e1003514. doi: 10.1371/journal.ppat.1003514 (PMC3738494; doi:10.1371/journal.ppat.1003514)
Supplement: Text S2 — Supporting tables. Table S1 – 358 Top 2.5% inhibitory and enhancing genes. Table S2 – Overlap of primary hit list with RNAi screens in other viruses. Table S3 – HFs identified by Y2H. Table S4 – Known interactors of HSV-1. Table S5 – Overlap between HSV-1 siRNA and Y2H screens. Table S6 –Validation of primary screen phenotypes by siRNA SMARTpool deconvolution and quantitative RT-PCR. Table S7 – Specificity of identified HFs to HSV-1 replication. Table S8 – Functional and pathway analysis of siRNA HFs. Table S9 – Primers and probes for qPCR assays. (DOCX) [file ppat.1003514.s002.docx]

**Text S2**

**Table S1:** Top 2.5% siRNA knockdown hits yielding strong inhibitory and enhancing phenotypes

| **Gene Symbol** | **Description** | **Mean*^a^*** | **STDEV*^b^*** |
| --- | --- | --- | --- |
| PRPF8 | PRP8 pre-mRNA processing factor 8 homolog (S. cerevisiae) | **0.00** | 0.06 |
| FUBP1 | far upstream element (FUSE) binding protein 1 | **0.00** | 0.06 |
| SYMPK | symplekin | **0.00** | 0.04 |
| NHP2L1 | NHP2 non-histone chromosome protein 2-like 1 (S. cerevisiae) | **0.00** | 0.04 |
| MYL4 | myosin, light chain 4, alkali; atrial, embryonic | **0.00** | 0.04 |
| SAA1 | serum amyloid A1 | **0.00** | 0.06 |
| RPS5 | ribosomal protein S5 | **0.00** | 0.04 |
| CLTB | clathrin, light chain (Lcb) | **0.00** | 0.05 |
| CCL1 | chemokine (C-C motif) ligand 1 | **0.00** | 0.03 |
| RPS2 | ribosomal protein S2 | **0.00** | 0.05 |
| LYZ | lysozyme (renal amyloidosis) | **0.00** | 0.02 |
| NF2 | neurofibromin 2 (merlin) | **0.00** | 0.05 |
| MSH4 | mutS homolog 4 (E. coli) | **0.00** | 0.06 |
| POLR2G | polymerase (RNA) II (DNA directed) polypeptide G | **0.00** | 0.03 |
| PRSS11 | HtrA serine peptidase 1 | **0.00** | 0.02 |
| MED6 | mediator complex subunit 6 | **0.00** | 0.01 |
| POLR2A | polymerase (RNA) II (DNA directed) polypeptide A, 220kDa | **0.00** | 0.03 |
| NUMA1 | nuclear mitotic apparatus protein 1 | **0.00** | 0.04 |
| SART1 | squamous cell carcinoma antigen recognized by T cells | **0.00** | 0.05 |
| ERH | enhancer of rudimentary homolog (Drosophila) | **0.00** | 0.02 |
| SFRS2 | splicing factor, arginine/serine-rich 2 | **0.00** | 0.03 |
| KIAA1622 | protein phosphatase 4, regulatory subunit 4 | **0.00** | 0.02 |
| DDX26 | integrator complex subunit 6 | **0.00** | 0.03 |
| RPS21 | ribosomal protein S21 | **0.00** | 0.05 |
| SFPQ | splicing factor proline/glutamine-rich (polypyrimidine tract binding protein associated) | **0.00** | 0.03 |
| IK | IK cytokine, down-regulator of HLA II | **0.00** | 0.02 |
| SON | SON DNA binding protein | **0.00** | 0.06 |
| LTBR | lymphotoxin beta receptor (TNFR superfamily, member 3) | **0.00** | 0.05 |
| SUPT5H | suppressor of Ty 5 homolog (S. cerevisiae) | **0.01** | 0.04 |
| DVL1 | dishevelled, dsh homolog 1 (Drosophila) | **0.01** | 0.03 |
| DUX3 | double homeobox, 3 | **0.01** | 0.03 |
| ING2 | inhibitor of growth family, X-linked, pseudogene | **0.01** | 0.03 |
| SLBP | stem-loop binding protein | **0.01** | 0.08 |
| SF3A3 | splicing factor 3a, subunit 3, 60kDa | **0.01** | 0.02 |
| XPO1 | exportin 1 (CRM1 homolog, yeast) | **0.01** | 0.06 |
| NOLC1 | nucleolar and coiled-body phosphoprotein 1 | **0.01** | 0.04 |
| CEACAM7 | carcinoembryonic antigen-related cell adhesion molecule 7 | **0.01** | 0.03 |
| SPTA1 | spectrin, alpha, erythrocytic 1 (elliptocytosis 2) | **0.01** | 0.06 |
| BIG1 | ADP-ribosylation factor guanine nucleotide-exchange factor 1(brefeldin A-inhibited) | **0.01** | 0.01 |
| PP | pyrophosphatase (inorganic) 1 | **0.01** | 0.01 |
| CCT7 | chaperonin containing TCP1, subunit 7 (eta) | **0.01** | 0.05 |
| HRK | harakiri, BCL2 interacting protein (contains only BH3 domain) | **0.01** | 0.01 |
| NTF3 | neurotrophin 3 | **0.01** | 0.01 |
| LISCH7 | lipolysis stimulated lipoprotein receptor | **0.01** | 0.04 |
| CRSP7 | mediator complex subunit 26 | **0.01** | 0.01 |
| KRT10 | keratin 10 | **0.01** | 0.05 |
| CYR61 | cysteine-rich, angiogenic inducer, 61 | **0.01** | 0.06 |
| SET8 | SET domain containing (lysine methyltransferase) 8 | **0.01** | 0.01 |
| CLCA2 | chloride channel accessory 2 | **0.01** | 0.04 |
| POLR2B | polymerase (RNA) II (DNA directed) polypeptide B, 140kDa | **0.01** | 0.02 |
| RPL5 | ribosomal protein L5 | **0.01** | 0.04 |
| SVIL | supervillin | **0.01** | 0.05 |
| CGA | glycoprotein hormones, alpha polypeptide | **0.01** | 0.03 |
| ALB | albumin | **0.01** | 0.02 |
| GPR147 |  | **0.01** | 0.07 |
| TRO | trophinin | **0.01** | 0.03 |
| CACNA1A | calcium channel, voltage-dependent, P/Q type, alpha 1A subunit | **0.01** | 0.01 |
| TRAF1 | TNF receptor-associated factor 1 | **0.01** | 0.01 |
| TBCA | tubulin folding cofactor A | **0.01** | 0.00 |
| SF3A1 | splicing factor 3a, subunit 1, 120kDa | **0.01** | 0.03 |
| CANX | calnexin | **0.01** | 0.06 |
| PTS | 6-pyruvoyltetrahydropterin synthase | **0.02** | 0.05 |
| RAN | RAN, member RAS oncogene family | **0.02** | 0.01 |
| DNC1I2 | dynein, cytoplasmic 1, intermediate chain 2 | **0.02** | 0.01 |
| PAFAH1B1 | platelet-activating factor acetylhydrolase, isoform Ib, alpha subunit 45kDa | **0.02** | 0.01 |
| RAD50 | RAD50 homolog (S. cerevisiae) | **0.02** | 0.03 |
| BAP1 | BRCA1 associated protein-1 (ubiquitin carboxy-terminal hydrolase) | **0.02** | 0.01 |
| ZFP64 | zinc finger protein 64 homolog (mouse) | **0.02** | 0.03 |
| RB1CC1 | RB1-inducible coiled-coil 1 | **0.02** | 0.05 |
| CFL1 | cofilin 1 (non-muscle) | **0.02** | 0.05 |
| ALDRL6 | myo-inositol oxygenase | **0.02** | 0.02 |
| CDC5L | CDC5 cell division cycle 5-like (S. pombe) | **0.02** | 0.06 |
| M96 | metal response element binding transcription factor 2 | **0.02** | 0.03 |
| CRYBB1 | crystallin, beta B1 | **0.02** | 0.02 |
| GPS1 | G protein pathway suppressor 1 | **0.02** | 0.04 |
| D1S155E | cold shock domain containing E1, RNA-binding | **0.02** | 0.04 |
| POLR2D | polymerase (RNA) II (DNA directed) polypeptide D | **0.02** | 0.01 |
| STK17A |  | **0.02** | 0.06 |
| HMGN1 | high-mobility group nucleosome binding domain 1 | **0.02** | 0.02 |
| CEBPB | CCAAT/enhancer binding protein (C/EBP), beta | **0.02** | 0.02 |
| GABRB1 | gamma-aminobutyric acid (GABA) A receptor, beta 1 | **0.02** | 0.01 |
| PDE4C | phosphodiesterase 4C, cAMP-specific (phosphodiesterase E1 dunce homolog, Drosophila) | **0.02** | 0.02 |
| SLC6A4 | solute carrier family 6 (neurotransmitter transporter, serotonin), member 4 | **0.02** | 0.03 |
| AXUD1 | AXIN1 up-regulated 1 | **0.02** | 0.03 |
| DBP | D site of albumin promoter (albumin D-box) binding protein | **0.02** | 0.02 |
| PVRL1 | poliovirus receptor-related 1 (herpesvirus entry mediator C) | **0.02** | 0.02 |
| DYSF | dysferlin, limb girdle muscular dystrophy 2B (autosomal recessive) | **0.02** | 0.02 |
| RPL10 | ribosomal protein L10 | **0.02** | 0.02 |
| SULF2 | sulfatase 2 | **0.02** | 0.01 |
| GRK4 |  | **0.02** | 0.02 |
| MED8 | mediator complex subunit 8 | **0.02** | 0.04 |
| LTBP3 | latent transforming growth factor beta binding protein 3 | **0.02** | 0.04 |
| CNTF | ciliary neurotrophic factor | **0.02** | 0.04 |
| PCDH8 | protocadherin 8 | **0.02** | 0.04 |
| CACNA2D1 | calcium channel, voltage-dependent, alpha 2/delta subunit 1 | **0.02** | 0.03 |
| HCFC1 | host cell factor C1 (VP16-accessory protein) | **0.02** | 0.03 |
| CRSP2 | mediator complex subunit 14 | **0.02** | 0.01 |
| SERPINF2 | serpin peptidase inhibitor, clade F (alpha-2 antiplasmin, pigment epithelium derived factor), member 2 | **0.02** | 0.03 |
| PON3 | paraoxonase 3 | **0.02** | 0.01 |
| PRPF31 | PRP31 pre-mRNA processing factor 31 homolog (S. cerevisiae) | **0.02** | 0.02 |
| RUNX2 | runt-related transcription factor 2 | **0.02** | 0.00 |
| MST1R |  | **0.02** | 0.02 |
| FUCA2 | fucosidase, alpha-L- 2, plasma | **0.02** | 0.03 |
| ARL5 | ADP-ribosylation factor-like 5A | **0.02** | 0.02 |
| ALPL | alkaline phosphatase, liver/bone/kidney | **0.02** | 0.01 |
| EVI2B | ecotropic viral integration site 2B | **0.02** | 0.02 |
| DCLRE1A | DNA cross-link repair 1A (PSO2 homolog, S. cerevisiae) | **0.02** | 0.02 |
| NUP98 | nucleoporin 98kDa | **0.02** | 0.04 |
| FLJ21924 | glutamine and serine rich 1 | **0.02** | 0.04 |
| DNAJA4 | DnaJ (Hsp40) homolog, subfamily A, member 4 | **0.02** | 0.04 |
| TAL1 | T-cell acute lymphocytic leukemia 1 | **0.02** | 0.05 |
| EFNA4 | ephrin-A4 | **0.02** | 0.02 |
| EIF3S9 | eukaryotic translation initiation factor 3, subunit B | **0.02** | 0.01 |
| CASP10 | caspase 10, apoptosis-related cysteine peptidase | **0.02** | 0.01 |
| ANAPC4 | anaphase promoting complex subunit 4 | **0.02** | 0.01 |
| CAPN2 | calpain 2, (m/II) large subunit | **0.02** | 0.01 |
| CRSP9 | mediator complex subunit 7 | **0.03** | 0.01 |
| EIF3S8 | eukaryotic translation initiation factor 3, subunit C | **0.03** | 0.01 |
| STK11 |  | **0.03** | 0.02 |
| KIAA1679 | thrombospondin, type I, domain containing 7B | **0.03** | 0.05 |
| SRMS |  | **0.03** | 0.01 |
| GABRR1 | gamma-aminobutyric acid (GABA) receptor, rho 1 | **0.03** | 0.01 |
| LMNA | lamin A/C | **0.03** | 0.05 |
| SFRS7 | splicing factor, arginine/serine-rich 7, 35kDa | **0.03** | 0.04 |
| RRM1 | ribonucleotide reductase M1 | **0.03** | 0.02 |
| NTSR1 |  | **0.03** | 0.02 |
| PDE6G | phosphodiesterase 6G, cGMP-specific, rod, gamma | **0.03** | 0.00 |
| FN3KRP |  | **0.03** | 0.01 |
| RECK | reversion-inducing-cysteine-rich protein with kazal motifs | **0.03** | 0.01 |
| ALCAM | activated leukocyte cell adhesion molecule | **0.03** | 0.03 |
| BCAS2 | breast carcinoma amplified sequence 2 | **0.03** | 0.07 |
| COPB2 |  | **0.03** | 0.01 |
| P2RX2 | purinergic receptor P2X, ligand-gated ion channel, 2 | **0.03** | 0.02 |
| ALAS2 | aminolevulinate, delta-, synthase 2 | **0.03** | 0.02 |
| FGFRL1 | fibroblast growth factor receptor-like 1 | **0.03** | 0.01 |
| NCOA4 | nuclear receptor coactivator 4 | **0.03** | 0.01 |
| THBS3 | thrombospondin 3 | **0.03** | 0.01 |
| HGFAC | HGF activator | **0.03** | 0.01 |
| HNRPUL1 | heterogeneous nuclear ribonucleoprotein U-like 1 | **0.03** | 0.09 |
| CSE1L | CSE1 chromosome segregation 1-like (yeast) | **0.03** | 0.02 |
| CCT8 | chaperonin containing TCP1, subunit 8 (theta) | **0.03** | 0.01 |
| NDE1 | nudE nuclear distribution gene E homolog 1 (A. nidulans) | **0.03** | 0.02 |
| RPS9 | ribosomal protein S9 | **0.03** | 0.06 |
| LOXL3 | lysyl oxidase-like 3 | **0.03** | 0.01 |
| TGM1 | transglutaminase 1 (K polypeptide epidermal type I, protein-glutamine-gamma-glutamyltransferase) | **0.03** | 0.03 |
| PLRG1 | pleiotropic regulator 1 (PRL1 homolog, Arabidopsis) | **0.03** | 0.01 |
| UBA52 | ubiquitin A-52 residue ribosomal protein fusion product 1 | **0.03** | 0.04 |
| VPS52 | vacuolar protein sorting 52 homolog (S. cerevisiae) | **0.03** | 0.01 |
| LMTK3 |  | **0.03** | 0.01 |
| EXT2 | exostoses (multiple) 2 | **0.03** | 0.02 |
| ARNT | aryl hydrocarbon receptor nuclear translocator | **0.03** | 0.01 |
| PLA2G1B | phospholipase A2, group IB (pancreas) | **0.03** | 0.01 |
| DOK5 | docking protein 5 | **0.03** | 0.03 |
| TAF4 | TAF4 RNA polymerase II, TATA box binding protein (TBP)-associated factor, 135kDa | **0.03** | 0.04 |
| LIM |  | **0.03** | 0.03 |
| GPR156 |  | **0.03** | 0.01 |
| CGI-63 | mitochondrial trans-2-enoyl-CoA reductase | **0.03** | 0.02 |
| PPP1R10 | protein phosphatase 1, regulatory (inhibitor) subunit 10 | **0.03** | 0.04 |
| CCL3L1 | chemokine (C-C motif) ligand 3-like 1 | **0.03** | 0.03 |
| SFRS3 | splicing factor, arginine/serine-rich 3 | **0.03** | 0.05 |
| MYF6 | myogenic factor 6 (herculin) | **0.03** | 0.01 |
| BIRC5 | baculoviral IAP repeat-containing 5 | **0.03** | 0.03 |
| PCOLCE | procollagen C-endopeptidase enhancer | **0.03** | 0.06 |
| BCL10 | B-cell CLL/lymphoma 10 | **0.03** | 0.04 |
| TULP2 | tubby like protein 2 | **0.03** | 0.02 |
| HLA-G | major histocompatibility complex, class I, G | **0.03** | 0.03 |
| APOBEC3F | apolipoprotein B mRNA editing enzyme, catalytic polypeptide-like 3F | **0.03** | 0.01 |
| PIGR | polymeric immunoglobulin receptor | **0.03** | 0.03 |
| GALK2 |  | **0.03** | 0.02 |
| PSMD2 | proteasome (prosome, macropain) 26S subunit, non-ATPase, 2 | **0.03** | 0.03 |
| ADAMTS10 | ADAM metallopeptidase with thrombospondin type 1 motif, 10 | **0.03** | 0.03 |
| DYNC1H1 | dynein, cytoplasmic 1, heavy chain 1 | **0.03** | 0.01 |
| GMEB1 | glucocorticoid modulatory element binding protein 1 | **0.03** | 0.03 |
| SMARCA5 | SWI/SNF related, matrix associated, actin dependent regulator of chromatin, subfamily a, member 5 | **0.03** | 0.02 |
| CDC26 | cell division cycle 26 homolog (S. cerevisiae) | **0.03** | 0.03 |
| SURB7 | mediator complex subunit 21 | **0.03** | 0.01 |
| TNFSF8 | tumor necrosis factor (ligand) superfamily, member 8 | **0.03** | 0.02 |
| CORT | cortistatin | **0.03** | 0.05 |
| FBXL6 | F-box and leucine-rich repeat protein 6 | **0.03** | 0.02 |
| GRAP2 | GRB2-related adaptor protein 2 | **1.07** | 0.21 |
| MOAP1 | modulator of apoptosis 1 | **1.07** | 0.42 |
| USP20 | ubiquitin specific peptidase 20 | **1.07** | 0.19 |
| ALDH9A1 | aldehyde dehydrogenase 9 family, member A1 | **1.07** | 0.17 |
| FER |  | **1.07** | 0.04 |
| LY64 | CD180 molecule | **1.07** | 0.28 |
| ARFGAP1 | ADP-ribosylation factor GTPase activating protein 1 | **1.07** | 0.10 |
| RAB3A | RAB3A, member RAS oncogene family | **1.07** | 0.09 |
| ERCC3 | excision repair cross-complementing rodent repair deficiency, complementation group 3 (xeroderma pigmentosum group B complementing) | **1.07** | 0.30 |
| PES1 | pescadillo homolog 1, containing BRCT domain (zebrafish) | **1.07** | 0.35 |
| TCFL1 | vacuolar protein sorting 72 homolog (S. cerevisiae) | **1.07** | 0.09 |
| CAPZA2 | capping protein (actin filament) muscle Z-line, alpha 2 | **1.07** | 0.16 |
| MAP4K4 |  | **1.07** | 0.16 |
| DC-UBP | ubiquitin domain containing 2 | **1.08** | 0.27 |
| HGF | hepatocyte growth factor (hepapoietin A; scatter factor) | **1.08** | 0.20 |
| SERF1A | small EDRK-rich factor 1A (telomeric) | **1.08** | 0.11 |
| PHF1 | PHD finger protein 1 | **1.08** | 0.19 |
| PTPN5 |  | **1.08** | 0.18 |
| KIAA1363 | arylacetamide deacetylase-like 1 | **1.08** | 0.09 |
| UBL3 | ubiquitin-like 3 | **1.08** | 0.21 |
| HIST2H2BE | histone cluster 2, H2be | **1.08** | 0.53 |
| ENAM | enamelin | **1.08** | 0.26 |
| PLEKHA1 | pleckstrin homology domain containing, family A (phosphoinositide binding specific) member 1 | **1.08** | 0.26 |
| CTF1 | cardiotrophin 1 | **1.09** | 0.25 |
| TCF7L2 | transcription factor 7-like 2 (T-cell specific, HMG-box) | **1.09** | 0.17 |
| RAB20 | RAB20, member RAS oncogene family | **1.09** | 0.16 |
| DNTTIP1 | deoxynucleotidyltransferase, terminal, interacting protein 1 | **1.09** | 0.34 |
| HERC6 | hect domain and RLD 6 | **1.09** | 0.16 |
| FH | fumarate hydratase | **1.09** | 0.25 |
| KCNF1 | potassium voltage-gated channel, subfamily F, member 1 | **1.09** | 0.16 |
| SIAH1 | seven in absentia homolog 1 (Drosophila) | **1.09** | 0.17 |
| RBL1 | retinoblastoma-like 1 (p107) | **1.10** | 0.25 |
| CDY2 | chromodomain protein, Y-linked, 2A | **1.10** | 0.24 |
| QDPR | quinoid dihydropteridine reductase | **1.10** | 0.18 |
| ARHGEF15 | Rho guanine nucleotide exchange factor (GEF) 15 | **1.10** | 0.23 |
| DLX5 | distal-less homeobox 5 | **1.10** | 0.51 |
| G3BP | GTPase activating protein (SH3 domain) binding protein 1 | **1.10** | 0.36 |
| DRG2 | developmentally regulated GTP binding protein 2 | **1.10** | 0.77 |
| HR | hairless homolog (mouse) | **1.11** | 0.34 |
| KCNJ11 | potassium inwardly-rectifying channel, subfamily J, member 11 | **1.11** | 0.14 |
| PLG | plasminogen | **1.11** | 0.21 |
| LMO7 | LIM domain 7 | **1.11** | 0.14 |
| ARL4A | ADP-ribosylation factor-like 4A | **1.11** | 0.15 |
| SLC12A3 | solute carrier family 12 (sodium/chloride transporters), member 3 | **1.11** | 0.25 |
| RYBP | RING1 and YY1 binding protein | **1.11** | 0.31 |
| PELP1 | proline, glutamate and leucine rich protein 1 | **1.11** | 0.47 |
| KIR3DL3 | killer cell immunoglobulin-like receptor, three domains, long cytoplasmic tail, 3 | **1.11** | 0.23 |
| DAXX | death-domain associated protein | **1.11** | 0.31 |
| MMP7 | matrix metallopeptidase 7 (matrilysin, uterine) | **1.11** | 0.25 |
| OAS1 | 2',5'-oligoadenylate synthetase 1, 40/46kDa | **1.11** | 0.41 |
| THRAP4 | mediator complex subunit 24 | **1.12** | 0.39 |
| RUNX3 | runt-related transcription factor 3 | **1.12** | 0.14 |
| BAD | BCL2-associated agonist of cell death | **1.12** | 0.20 |
| CDH18 | cadherin 18, type 2 | **1.12** | 0.28 |
| RAPSN | receptor-associated protein of the synapse | **1.12** | 0.19 |
| SLC8A1 | solute carrier family 8 (sodium/calcium exchanger), member 1 | **1.12** | 0.22 |
| CDKN2A | cyclin-dependent kinase inhibitor 2A (melanoma, p16, inhibits CDK4) | **1.13** | 0.21 |
| OR1D5 |  | **1.13** | 0.07 |
| TH | tyrosine hydroxylase | **1.13** | 0.34 |
| UCP1 | uncoupling protein 1 (mitochondrial, proton carrier) | **1.13** | 0.26 |
| FLJ35107 |  | **1.13** | 0.16 |
| CSK |  | **1.13** | 0.21 |
| PRSS2 | protease, serine, 2 (trypsin 2) | **1.13** | 0.22 |
| POLR3D | polymerase (RNA) III (DNA directed) polypeptide D, 44kDa | **1.13** | 0.33 |
| C20ORF46 | chromosome 20 open reading frame 46 | **1.13** | 0.31 |
| FKBP4 | FK506 binding protein 4, 59kDa | **1.13** | 0.36 |
| KAI1 | CD82 molecule | **1.14** | 0.22 |
| C6ORF108 | chromosome 6 open reading frame 108 | **1.14** | 0.16 |
| BAIAP2 | BAI1-associated protein 2 | **1.14** | 0.27 |
| AXIN2 | axin 2 | **1.14** | 0.21 |
| C6 | complement component 6 | **1.14** | 0.27 |
| ASPM | asp (abnormal spindle) homolog, microcephaly associated (Drosophila) | **1.14** | 0.21 |
| PTTG2 | pituitary tumor-transforming 2 | **1.15** | 0.23 |
| CR2 | complement component (3d/Epstein Barr virus) receptor 2 | **1.15** | 0.10 |
| LIPC | lipase, hepatic | **1.15** | 0.23 |
| WHSC2 | Wolf-Hirschhorn syndrome candidate 2 | **1.15** | 0.26 |
| DDB1 | damage-specific DNA binding protein 1, 127kDa | **1.15** | 0.21 |
| MYCL1 | v-myc myelocytomatosis viral oncogene homolog 1, lung carcinoma derived (avian) | **1.15** | 0.12 |
| H1F0 | H1 histone family, member 0 | **1.15** | 0.50 |
| PHOX2A | paired-like homeobox 2a | **1.16** | 0.27 |
| SLC26A3 | solute carrier family 26, member 3 | **1.16** | 0.17 |
| JARID2 | jumonji, AT rich interactive domain 2 | **1.16** | 0.26 |
| CDH20 | cadherin 20, type 2 | **1.16** | 0.28 |
| CAPS | calcyphosine | **1.16** | 0.26 |
| TBX21 | T-box 21 | **1.16** | 0.17 |
| UBE2R2 | ubiquitin-conjugating enzyme E2R 2 | **1.16** | 0.14 |
| KRT12 | keratin 12 | **1.17** | 0.19 |
| ZBTB7 | zinc finger and BTB domain containing 7A | **1.17** | 0.34 |
| RAPGEFL1 | Rap guanine nucleotide exchange factor (GEF)-like 1 | **1.18** | 0.21 |
| ARHGDIB | Rho GDP dissociation inhibitor (GDI) beta | **1.18** | 0.25 |
| LGR6 | leucine-rich repeat-containing G protein-coupled receptor 6 | **1.18** | 0.16 |
| SH3BP5 | SH3-domain binding protein 5 (BTK-associated) | **1.18** | 0.28 |
| CCL14 | chemokine (C-C motif) ligand 14 | **1.19** | 0.25 |
| NKX2-8 | NK2 homeobox 8 | **1.19** | 0.12 |
| FGF3 | fibroblast growth factor 3 (murine mammary tumor virus integration site (v-int-2) oncogene homolog) | **1.19** | 0.35 |
| UBE3C | ubiquitin protein ligase E3C | **1.20** | 0.44 |
| ATRX | alpha thalassemia/mental retardation syndrome X-linked (RAD54 homolog, S. cerevisiae) | **1.20** | 0.23 |
| THEDC1 | oleoyl-ACP hydrolase | **1.20** | 0.23 |
| HAVCR1 | hepatitis A virus cellular receptor 1 | **1.20** | 0.29 |
| SOCS4 | suppressor of cytokine signaling 4 | **1.20** | 0.23 |
| COL4A1 | collagen, type IV, alpha 1 | **1.20** | 0.19 |
| RAC1 |  | **1.20** | 0.19 |
| DEPC-1 | alkB, alkylation repair homolog 3 (E. coli) | **1.20** | 0.23 |
| HOXD4 | homeobox D4 | **1.21** | 0.14 |
| E2F6 | E2F transcription factor 6 | **1.21** | 0.16 |
| WHSC1L1 | Wolf-Hirschhorn syndrome candidate 1-like 1 | **1.21** | 0.13 |
| SKIL | SKI-like oncogene | **1.21** | 0.23 |
| PRDX2 | peroxiredoxin 2 | **1.21** | 0.18 |
| SHBG | sex hormone-binding globulin | **1.22** | 0.08 |
| RASGRP3 | RAS guanyl releasing protein 3 (calcium and DAG-regulated) | **1.22** | 0.25 |
| RBKS |  | **1.22** | 0.17 |
| DNYNC1I1 | dynein, cytoplasmic 1, intermediate chain 1 | **1.23** | 0.20 |
| KIF2 | kinesin heavy chain member 2A | **1.23** | 0.14 |
| EPB42 | erythrocyte membrane protein band 4.2 | **1.23** | 0.17 |
| PPP2R2C | protein phosphatase 2 (formerly 2A), regulatory subunit B, gamma isoform | **1.23** | 0.30 |
| MC2R |  | **1.23** | 0.19 |
| CCNB3 | cyclin B3 | **1.23** | 0.39 |
| MYL3 | myosin, light chain 3, alkali; ventricular, skeletal, slow | **1.23** | 0.32 |
| DLG7 | discs, large (Drosophila) homolog-associated protein 5 | **1.24** | 0.23 |
| PNRC2 | proline-rich nuclear receptor coactivator 2 | **1.24** | 0.33 |
| GLIPR1 | GLI pathogenesis-related 1 | **1.24** | 0.21 |
| USP19 | ubiquitin specific peptidase 19 | **1.26** | 0.14 |
| MYO5A | myosin VA (heavy chain 12, myoxin) | **1.26** | 0.33 |
| DRIM | UTP20, small subunit (SSU) processome component, homolog (yeast) | **1.26** | 0.29 |
| PPP4R2 | protein phosphatase 4, regulatory subunit 2 | **1.27** | 0.45 |
| SPINT2 | serine peptidase inhibitor, Kunitz type, 2 | **1.27** | 0.26 |
| CBFA2T2 | core-binding factor, runt domain, alpha subunit 2; translocated to, 2 | **1.28** | 0.17 |
| CFC1 | cripto, FRL-1, cryptic family 1 | **1.28** | 0.35 |
| NRGN | neurogranin (protein kinase C substrate, RC3) | **1.29** | 0.19 |
| MLF2 | myeloid leukemia factor 2 | **1.29** | 0.36 |
| MGC40069 | hypothetical protein MGC40069 | **1.29** | 0.36 |
| DFNA5 | deafness, autosomal dominant 5 | **1.29** | 0.30 |
| PCDHB14 | protocadherin beta 14 | **1.30** | 0.16 |
| GAS8 | growth arrest-specific 8 | **1.30** | 0.35 |
| DLGAP1 | discs, large (Drosophila) homolog-associated protein 1 | **1.31** | 0.21 |
| CLCNKB | chloride channel Kb | **1.31** | 0.37 |
| FLJ11535 | plasticity-related gene 2 | **1.31** | 0.20 |
| STAT3 | signal transducer and activator of transcription 3 (acute-phase response factor) | **1.32** | 0.23 |
| PHEMX | tetraspanin 32 | **1.32** | 0.29 |
| SHFM1 | split hand/foot malformation (ectrodactyly) type 1 | **1.33** | 0.24 |
| FBXO9 | F-box protein 9 | **1.33** | 0.34 |
| HSPCA | heat shock protein 90kDa alpha (cytosolic), class A member 1 | **1.34** | 0.20 |
| BHMT | betaine-homocysteine methyltransferase | **1.35** | 0.28 |
| CAP1 | CAP, adenylate cyclase-associated protein 1 (yeast) | **1.35** | 0.38 |
| UBL4 | ubiquitin-like 4A | **1.37** | 0.20 |
| HMX1 | H6 family homeobox 1 | **1.37** | 0.41 |
| FOXA1 | forkhead box A1 | **1.38** | 0.29 |
| FAIM | Fas apoptotic inhibitory molecule | **1.39** | 0.22 |
| COL4A6 | collagen, type IV, alpha 6 | **1.39** | 0.30 |
| TADA2L | transcriptional adaptor 2 (ADA2 homolog, yeast)-like | **1.39** | 0.24 |
| DRG1 | developmentally regulated GTP binding protein 1 | **1.39** | 0.62 |
| FCER2 | Fc fragment of IgE, low affinity II, receptor for (CD23) | **1.39** | 0.19 |
| TAS2R14 |  | **1.39** | 0.08 |
| NUCKS | nuclear casein kinase and cyclin-dependent kinase substrate 1 | **1.40** | 0.40 |
| APBB3 | amyloid beta (A4) precursor protein-binding, family B, member 3 | **1.40** | 0.49 |
| PDE6B | phosphodiesterase 6B, cGMP-specific, rod, beta | **1.40** | 0.36 |
| HOXC9 | homeobox C9 | **1.43** | 0.20 |
| PSPH | phosphoserine phosphatase | **1.46** | 0.14 |
| SOX4 | SRY (sex determining region Y)-box 4 | **1.47** | 0.34 |
| NR3C2 | nuclear receptor subfamily 3, group C, member 2 | **1.47** | 0.43 |
| HSXIAPAF1 | XIAP associated factor 1 | **1.48** | 0.34 |
| LOX | lysyl oxidase | **1.48** | 0.42 |
| AGTRAP | angiotensin II receptor-associated protein | **1.48** | 0.25 |
| STAM2 | signal transducing adaptor molecule (SH3 domain and ITAM motif) 2 | **1.49** | 0.30 |
| GCKR | glucokinase (hexokinase 4) regulator | **1.50** | 0.22 |
| CBX5 | chromobox homolog 5 (HP1 alpha homolog, Drosophila) | **1.52** | 0.51 |
| HOXA10 | homeobox A10 | **1.53** | 0.27 |
| GTF3C1 | general transcription factor IIIC, polypeptide 1, alpha 220kDa | **1.53** | 0.22 |
| PM5 | NODAL modulator 1 | **1.53** | 0.41 |
| PSMD5 | proteasome (prosome, macropain) 26S subunit, non-ATPase, 5 | **1.56** | 0.39 |
| GP5 | glycoprotein V (platelet) | **1.56** | 0.37 |
| TOP3B | topoisomerase (DNA) III beta | **1.58** | 0.54 |
| ICAM5 | intercellular adhesion molecule 5, telencephalin | **1.59** | 0.50 |
| NDN | necdin homolog (mouse) | **1.65** | 0.24 |
| CRSP3 | mediator complex subunit 23 | **1.68** | 0.31 |
| PTPN14 | protein tyrosine phosphatase, non-receptor type 14 | **1.70** | 0.34 |
| IFNAR1 | interferon (alpha, beta and omega) receptor 1 | **1.75** | 0.28 |
| TRIP10 | thyroid hormone receptor interactor 10 | **1.84** | 0.21 |
| CD53 | CD53 molecule | **2.03** | 0.42 |

*^a^* Mean slope of replication during the linear growth phase over 6 individual assay replicates.

*^b^* Standard deviation of the mean replication slopes.

**Table S2:** Overlap of primary hit list with RNAi screens in other viruses

| **Symbol** | **Description** | **Virus (Reference)** |
| --- | --- | --- |
| PRPF8 | PRP8 pre-mRNA processing factor 8 homolog (S. cerevisiae) | Inf A [[1](#_ENREF_1),[2](#_ENREF_2)], HIV [[3](#_ENREF_3)] |
| SYMPK | symplekin | WNV [[4](#_ENREF_4)] |
| NHP2L1 | NHP2 non-histone chromosome protein 2-like 1 (S. cerevisiae) | Inf A [[1](#_ENREF_1),[5](#_ENREF_5)] |
| RPS5 | ribosomal protein S5 | Inf A [[2](#_ENREF_2)] |
| NF2 | neurofibromin 2 (merlin) | HIV [[6](#_ENREF_6)] |
| MED6 | mediator complex subunit 6 | Inf A [[2](#_ENREF_2),[6](#_ENREF_6)], HIV [[3](#_ENREF_3),[7](#_ENREF_7)] |
| POLR2A | polymerase (RNA) II (DNA directed) polypeptide A, 220kDa | HIV [[3](#_ENREF_3)] |
| SART1 | squamous cell carcinoma antigen recognized by T cells | Inf A [[1](#_ENREF_1)] |
| SFRS2 | splicing factor, arginine/serine-rich 2 | HIV [[7](#_ENREF_7)] |
| INTS6 | integrator complex subunit 6 | HIV [[3](#_ENREF_3)] |
| IK | IK cytokine, down-regulator of HLA II | Inf A [[2](#_ENREF_2)] |
| SON | SON DNA binding protein | Inf A [[2](#_ENREF_2)] |
| DVL1 | dishevelled, dsh homolog 1 (Drosophila) | HIV [[7](#_ENREF_7)] |
| XPO1 | exportin 1 (CRM1 homolog, yeast) | Inf A [[2](#_ENREF_2)], HIV [[7](#_ENREF_7)] |
| HRK | harakiri, BCL2 interacting protein (contains only BH3 domain) | Inf A [[1](#_ENREF_1)] |
| MED26 | mediator complex subunit 26 | HIV [[7](#_ENREF_7)] |
| CACNA1A | calcium channel, voltage-dependent, P/Q type, alpha 1A subunit | WNV [[4](#_ENREF_4)] |
| SF3A1 | splicing factor 3a, subunit 1, 120kDa | Inf A [[2](#_ENREF_2),[5](#_ENREF_5)], HIV [[3](#_ENREF_3)] |
| PTS | 6-pyruvoyltetrahydropterin synthase | Inf A [[5](#_ENREF_5)] |
| RAN | RAN, member RAS oncogene family | HCV [[8](#_ENREF_8)] |
| RB1CC1 | RB1-inducible coiled-coil 1 | HIV [[7](#_ENREF_7)] |
| DYSF | dysferlin, limb girdle muscular dystrophy 2B (autosomal recessive) | HIV [[6](#_ENREF_6)] |
| SULF2 | sulfatase 2 | Inf A [[2](#_ENREF_2)] |
| MED8 | mediator complex subunit 8 | HIV [[7](#_ENREF_7)] |
| MED14 | mediator complex subunit 14 | Inf A [[2](#_ENREF_2)], HIV [[3](#_ENREF_3),[6](#_ENREF_6)] |
| PRPF31 | PRP31 pre-mRNA processing factor 31 homolog (S. cerevisiae) | Inf A [[1](#_ENREF_1)] |
| MST1R | macrophage stimulating 1 receptor (c-met-related tyrosine kinase) | Inf A [[2](#_ENREF_2)] |
| NUP98 | nucleoporin 98kDa | Inf A [[1](#_ENREF_1),[2](#_ENREF_2)], HIV [[3](#_ENREF_3)] |
| MED7 | mediator complex subunit 7 | HIV [[3](#_ENREF_3),[6](#_ENREF_6),[7](#_ENREF_7)] |
| EIF3C | eukaryotic translation initiation factor 3, subunit C | Inf A [[2](#_ENREF_2)] |
| NTSR1 | neurotensin receptor 1 (high affinity) | Inf A [[5](#_ENREF_5)] |
| COPB2 | coatomer protein complex, subunit beta 2 (beta prime) | Inf A [[1](#_ENREF_1),[2](#_ENREF_2)], Inf A [[5](#_ENREF_5)], HCV [[8](#_ENREF_8)] |
| CSE1L | CSE1 chromosome segregation 1-like (yeast) | Inf A [[5](#_ENREF_5)] |
| TGM1 | transglutaminase 1 (K polypeptide epidermal type I, protein-glutamine-gamma-glutamyltransferase) | HCV [[8](#_ENREF_8)] |
| UBA52 | ubiquitin A-52 residue ribosomal protein fusion product 1 | HCV [[9](#_ENREF_9)] |
| PSMD2 | proteasome (prosome, macropain) 26S subunit, non-ATPase, 2 | Inf A [[2](#_ENREF_2)] |
| DYNC1H1 | dynein, cytoplasmic 1, heavy chain 1 | HIV [[7](#_ENREF_7)] |
| USP20 | ubiquitin specific peptidase 20 | HIV [[7](#_ENREF_7)] |
| ERCC3 | excision repair cross-complementing rodent repair deficiency, complementation group 3 | HIV [[6](#_ENREF_6)] |
| MAP4K4 | mitogen-activated protein kinase kinase kinase kinase 4 | Inf A [[5](#_ENREF_5)] |
| PHF1 | PHD finger protein 1 | HIV [[7](#_ENREF_7)] |
| FH | fumarate hydratase | Dengue [[10](#_ENREF_10)] |
| KCNJ11 | potassium inwardly-rectifying channel, subfamily J, member 11 | Inf A [[5](#_ENREF_5)], HIV [[3](#_ENREF_3)] |
| BAIAP2 | BAI1-associated protein 2 | Inf A [[2](#_ENREF_2)] |
| TBX21 | T-box 21 | Inf A [[2](#_ENREF_2)] |
| RAC1 | ras-related C3 botulinum toxin substrate 1 (rho family, small GTP binding protein Rac1) | HCV [[9](#_ENREF_9)] |
| ALKBH3 | alkB, alkylation repair homolog 3 (E. coli) | HIV [[3](#_ENREF_3)] |
| DYNC1I1 | dynein, cytoplasmic 1, intermediate chain 1 | Inf A [[1](#_ENREF_1)] |
| CCNB3 | cyclin B3 | Inf A [[2](#_ENREF_2)] |

| STAT3 | signal transducer and activator of transcription 3 | HCV {Li, 2009 #1110} |
| --- | --- | --- |
| HSP90AA1 | heat shock protein 90kDa alpha (cytosolic), class A member 1 | Inf A [[5](#_ENREF_5)] |
| HMX1 | H6 family homeobox 1 | HIV [[7](#_ENREF_7)] |
| AGTRAP | angiotensin II receptor-associated protein | Inf A [[5](#_ENREF_5)] |
| TOP3B | topoisomerase (DNA) III beta | HIV [[3](#_ENREF_3)] |
| PRPF8 | PRP8 pre-mRNA processing factor 8 homolog (S. cerevisiae) | Inf A [[1](#_ENREF_1),[2](#_ENREF_2)], HIV [[3](#_ENREF_3)] |

**Table S3:** HFs identified by Y2H

| **HSV-1 Gene*^a^*** | **Cellular Gene*^b^*** | **Description** | **GeneID*^c^*** |
| --- | --- | --- | --- |
| **High Confidence Interactions** | | | |
| US10 | ZBED1 | zinc finger, BED-type containing 1 | [9189](http://www.ncbi.nlm.nih.gov/entrez/query.fcgi?db=gene&cmd=Retrieve&dopt=full_report&list_uids=9189) |
| UL2 | CTBP1 | C-terminal binding protein 1 | [1487](http://www.ncbi.nlm.nih.gov/entrez/query.fcgi?db=gene&cmd=Retrieve&dopt=full_report&list_uids=1487) |
| UL2 | CTBP2 | C-terminal binding protein 2 | [1488](http://www.ncbi.nlm.nih.gov/entrez/query.fcgi?db=gene&cmd=Retrieve&dopt=full_report&list_uids=1488) |
| UL54 | PRDM14 | PR domain containing 14 | [63978](http://www.ncbi.nlm.nih.gov/entrez/query.fcgi?db=gene&cmd=Retrieve&dopt=full_report&list_uids=63978) |
| UL33 | USHBP1 | Usher syndrome 1C binding protein 1 | [83878](http://www.ncbi.nlm.nih.gov/entrez/query.fcgi?db=gene&cmd=Retrieve&dopt=full_report&list_uids=83878) |
| UL35 | MAGEA1 | melanoma antigen family A, 1 (directs expression of antigen MZ2-E) | [4100](http://www.ncbi.nlm.nih.gov/entrez/query.fcgi?db=gene&cmd=Retrieve&dopt=full_report&list_uids=4100) |
| UL26 | C1orf94 | chromosome 1 open reading frame 94 | [84970](http://www.ncbi.nlm.nih.gov/entrez/query.fcgi?db=gene&cmd=Retrieve&dopt=full_report&list_uids=84970) |
| UL12 | USP15 | ubiquitin specific peptidase 15 | [9958](http://www.ncbi.nlm.nih.gov/entrez/query.fcgi?db=gene&cmd=Retrieve&dopt=full_report&list_uids=9958) |
| UL47 | RBCK1 | RanBP-type and C3HC4-type zinc finger containing 1 | [10616](http://www.ncbi.nlm.nih.gov/entrez/query.fcgi?db=gene&cmd=Retrieve&dopt=full_report&list_uids=10616) |
| UL39 | DYNLL2 | dynein, light chain, LC8-type 2 | [140735](http://www.ncbi.nlm.nih.gov/entrez/query.fcgi?db=gene&cmd=Retrieve&dopt=full_report&list_uids=140735) |
| UL39 | DYNLL1 | dynein, light chain, LC8-type 1 | [8655](http://www.ncbi.nlm.nih.gov/entrez/query.fcgi?db=gene&cmd=Retrieve&dopt=full_report&list_uids=8655) |
| UL10 | MDFI | MyoD family inhibitor | [4188](http://www.ncbi.nlm.nih.gov/entrez/query.fcgi?db=gene&cmd=Retrieve&dopt=full_report&list_uids=4188) |
| UL10 | FATE1 | fetal and adult testis expressed 1 | [89885](http://www.ncbi.nlm.nih.gov/entrez/query.fcgi?db=gene&cmd=Retrieve&dopt=full_report&list_uids=89885) |
| UL31 | RAD51 | RAD51 homolog (RecA homolog, E. coli) (S. cerevisiae) | [5888](http://www.ncbi.nlm.nih.gov/entrez/query.fcgi?db=gene&cmd=Retrieve&dopt=full_report&list_uids=5888) |
| UL47 | CSRNP1 | cysteine-serine-rich nuclear protein 1 | [64651](http://www.ncbi.nlm.nih.gov/entrez/query.fcgi?db=gene&cmd=Retrieve&dopt=full_report&list_uids=64651) |
| US2 | PSMC3 | proteasome (prosome, macropain) 26S subunit, ATPase, 3 | [5702](http://www.ncbi.nlm.nih.gov/entrez/query.fcgi?db=gene&cmd=Retrieve&dopt=full_report&list_uids=5702) |
| UL36 | HGS | hepatocyte growth factor-regulated tyrosine kinase substrate | [9146](http://www.ncbi.nlm.nih.gov/entrez/query.fcgi?db=gene&cmd=Retrieve&dopt=full_report&list_uids=9146) |
| UL31 | TRIP6 | thyroid hormone receptor interactor 6 | [7205](http://www.ncbi.nlm.nih.gov/entrez/query.fcgi?db=gene&cmd=Retrieve&dopt=full_report&list_uids=7205) |
| UL56 | GOPC | golgi associated PDZ and coiled-coil motif containing | [57120](http://www.ncbi.nlm.nih.gov/entrez/query.fcgi?db=gene&cmd=Retrieve&dopt=full_report&list_uids=57120) |
| UL10 | AHCYL1 | S-adenosylhomocysteine hydrolase-like 1 | [10768](http://www.ncbi.nlm.nih.gov/entrez/query.fcgi?db=gene&cmd=Retrieve&dopt=full_report&list_uids=10768) |
| UL10 | LZTS2 | leucine zipper, putative tumor suppressor 2 | [84445](http://www.ncbi.nlm.nih.gov/entrez/query.fcgi?db=gene&cmd=Retrieve&dopt=full_report&list_uids=84445) |
| RL2 | FAM164A | family with sequence similarity 164, member A | [51101](http://www.ncbi.nlm.nih.gov/entrez/query.fcgi?db=gene&cmd=Retrieve&dopt=full_report&list_uids=51101) |
| UL7 | EIF4ENIF1 | eukaryotic translation initiation factor 4E nuclear import factor 1 | [56478](http://www.ncbi.nlm.nih.gov/entrez/query.fcgi?db=gene&cmd=Retrieve&dopt=full_report&list_uids=56478) |
| UL10 | CSK | c-src tyrosine kinase | [1445](http://www.ncbi.nlm.nih.gov/entrez/query.fcgi?db=gene&cmd=Retrieve&dopt=full_report&list_uids=1445) |
| UL17 | PRR22 | PRR22 proline rich 22 | [163154](http://www.ncbi.nlm.nih.gov/entrez/query.fcgi?db=gene&cmd=Retrieve&dopt=full_report&list_uids=163154) |
| UL33 | C10orf96 | chromosome 10 open reading frame 96 | [374355](http://www.ncbi.nlm.nih.gov/entrez/query.fcgi?db=gene&cmd=Retrieve&dopt=full_report&list_uids=374355) |
| UL56 | WWP2 | WW domain containing E3 ubiquitin protein ligase 2 | [11060](http://www.ncbi.nlm.nih.gov/entrez/query.fcgi?db=gene&cmd=Retrieve&dopt=full_report&list_uids=11060) |
| US1 | THAP1 | THAP domain containing, apoptosis associated protein 1 | [55145](http://www.ncbi.nlm.nih.gov/entrez/query.fcgi?db=gene&cmd=Retrieve&dopt=full_report&list_uids=55145) |
| UL34 | FATE1 | fetal and adult testis expressed 1 | [89885](http://www.ncbi.nlm.nih.gov/entrez/query.fcgi?db=gene&cmd=Retrieve&dopt=full_report&list_uids=89885) |
| UL34 | GOLGA2 | golgi autoantigen, golgin subfamily a, 2 | [2801](http://www.ncbi.nlm.nih.gov/entrez/query.fcgi?db=gene&cmd=Retrieve&dopt=full_report&list_uids=2801) |
| UL33 | GMPPA | GDP-mannose pyrophosphorylase A | [29926](http://www.ncbi.nlm.nih.gov/entrez/query.fcgi?db=gene&cmd=Retrieve&dopt=full_report&list_uids=29926) |
| UL7 | SESTD1 | SEC14 and spectrin domains 1 | [91404](http://www.ncbi.nlm.nih.gov/entrez/query.fcgi?db=gene&cmd=Retrieve&dopt=full_report&list_uids=91404) |
| UL25 | HAUS6 | HAUS augmin-like complex, subunit 6 | [54801](http://www.ncbi.nlm.nih.gov/entrez/query.fcgi?db=gene&cmd=Retrieve&dopt=full_report&list_uids=54801) |
| UL46 | IKBKG | inhibitor of kappa light polypeptide gene enhancer in B-cells, kinase gamma | [8517](http://www.ncbi.nlm.nih.gov/entrez/query.fcgi?db=gene&cmd=Retrieve&dopt=full_report&list_uids=8517) |
| UL38 | ELAVL3 | ELAV (embryonic lethal, abnormal vision, Drosophila)-like 3 (Hu antigen C) | [1995](http://www.ncbi.nlm.nih.gov/entrez/query.fcgi?db=gene&cmd=Retrieve&dopt=full_report&list_uids=1995) |
| UL21 | ELAVL2 | ELAV (embryonic lethal, abnormal vision, Drosophila)-like 2 (Hu antigen B) | [1993](http://www.ncbi.nlm.nih.gov/entrez/query.fcgi?db=gene&cmd=Retrieve&dopt=full_report&list_uids=1993) |
| UL21 | ELAVL3 | ELAV (embryonic lethal, abnormal vision, Drosophila)-like 3 (Hu antigen C) | [1995](http://www.ncbi.nlm.nih.gov/entrez/query.fcgi?db=gene&cmd=Retrieve&dopt=full_report&list_uids=1995) |
| RL2 | DPPA2 | developmental pluripotency associated 2 | [151871](http://www.ncbi.nlm.nih.gov/entrez/query.fcgi?db=gene&cmd=Retrieve&dopt=full_report&list_uids=151871) |
| RL2 | TRAIP | TRAF interacting protein | [10293](http://www.ncbi.nlm.nih.gov/entrez/query.fcgi?db=gene&cmd=Retrieve&dopt=full_report&list_uids=10293) |
| UL20 | CBR3 | carbonyl reductase 3 | [874](http://www.ncbi.nlm.nih.gov/entrez/query.fcgi?db=gene&cmd=Retrieve&dopt=full_report&list_uids=874) |
| UL20 | CBR1 | carbonyl reductase 1 | [873](http://www.ncbi.nlm.nih.gov/entrez/query.fcgi?db=gene&cmd=Retrieve&dopt=full_report&list_uids=873) |
| UL22 | HINT2 | histidine triad nucleotide binding protein 2 | [84681](http://www.ncbi.nlm.nih.gov/entrez/query.fcgi?db=gene&cmd=Retrieve&dopt=full_report&list_uids=84681) |
| UL22 | HK2 | hexokinase 2 | [3099](http://www.ncbi.nlm.nih.gov/entrez/query.fcgi?db=gene&cmd=Retrieve&dopt=full_report&list_uids=3099) |
| UL22 | HMGCL | 3-hydroxymethyl-3-methylglutaryl-Coenzyme A lyase | [3155](http://www.ncbi.nlm.nih.gov/entrez/query.fcgi?db=gene&cmd=Retrieve&dopt=full_report&list_uids=3155) |
| UL22 | KCTD4 | potassium channel tetramerisation domain containing 4 | [386618](http://www.ncbi.nlm.nih.gov/entrez/query.fcgi?db=gene&cmd=Retrieve&dopt=full_report&list_uids=386618) |
| UL22 | NUSAP1 | nucleolar and spindle associated protein 1 | [51203](http://www.ncbi.nlm.nih.gov/entrez/query.fcgi?db=gene&cmd=Retrieve&dopt=full_report&list_uids=51203) |
| UL44 | SH3GL2 | SH3-domain GRB2-like 2 | [6456](http://www.ncbi.nlm.nih.gov/entrez/query.fcgi?db=gene&cmd=Retrieve&dopt=full_report&list_uids=6456) |
| UL10 | SAT1 | spermidine/spermine N1-acetyltransferase 1 | [6303](http://www.ncbi.nlm.nih.gov/entrez/query.fcgi?db=gene&cmd=Retrieve&dopt=full_report&list_uids=6303) |
| UL22 | SAA1 | serum amyloid A1 | [6288](http://www.ncbi.nlm.nih.gov/entrez/query.fcgi?db=gene&cmd=Retrieve&dopt=full_report&list_uids=6288) |
| UL46 | NONO | non-POU domain containing, octamer-binding | [4841](http://www.ncbi.nlm.nih.gov/entrez/query.fcgi?db=gene&cmd=Retrieve&dopt=full_report&list_uids=4841) |
| UL17 | MEIS2 | Meis homeobox 2 | [4212](http://www.ncbi.nlm.nih.gov/entrez/query.fcgi?db=gene&cmd=Retrieve&dopt=full_report&list_uids=4212) |
| UL34 | CEP70 | centrosomal protein 70kDa | [80321](http://www.ncbi.nlm.nih.gov/entrez/query.fcgi?db=gene&cmd=Retrieve&dopt=full_report&list_uids=80321) |
| UL56 | WTAP | Wilms tumor 1 associated protein | [9589](http://www.ncbi.nlm.nih.gov/entrez/query.fcgi?db=gene&cmd=Retrieve&dopt=full_report&list_uids=9589) |
| US11 | SH3KBP1 | SH3-domain kinase binding protein 1 | [30011](http://www.ncbi.nlm.nih.gov/entrez/query.fcgi?db=gene&cmd=Retrieve&dopt=full_report&list_uids=30011) |
| UL22 | MRPL24 | mitochondrial ribosomal protein L24 | [79590](http://www.ncbi.nlm.nih.gov/entrez/query.fcgi?db=gene&cmd=Retrieve&dopt=full_report&list_uids=79590) |
| UL40 | MCRS1 | microspherule protein 1 | [10445](http://www.ncbi.nlm.nih.gov/entrez/query.fcgi?db=gene&cmd=Retrieve&dopt=full_report&list_uids=10445) |
| UL53 | LZTS2 | leucine zipper, putative tumor suppressor 2 | [84445](http://www.ncbi.nlm.nih.gov/entrez/query.fcgi?db=gene&cmd=Retrieve&dopt=full_report&list_uids=84445) |
| UL56 | BOLL | bol, boule-like (Drosophila) | [66037](http://www.ncbi.nlm.nih.gov/entrez/query.fcgi?db=gene&cmd=Retrieve&dopt=full_report&list_uids=66037) |
| UL7 | CDR2 | cerebellar degeneration-related protein 2, 62kDa | [1039](http://www.ncbi.nlm.nih.gov/entrez/query.fcgi?db=gene&cmd=Retrieve&dopt=full_report&list_uids=1039) |
| US8 | PRDM14 | PR domain containing 14 | [63978](http://www.ncbi.nlm.nih.gov/entrez/query.fcgi?db=gene&cmd=Retrieve&dopt=full_report&list_uids=63978) |
| UL43 | PLSCR1 | phospholipid scramblase 1 | [5359](http://www.ncbi.nlm.nih.gov/entrez/query.fcgi?db=gene&cmd=Retrieve&dopt=full_report&list_uids=5359) |
| UL34 | PNMA1 | paraneoplastic antigen MA1 | [9240](http://www.ncbi.nlm.nih.gov/entrez/query.fcgi?db=gene&cmd=Retrieve&dopt=full_report&list_uids=9240) |
| UL34 | CALCOCO2 | calcium binding and coiled-coil domain 2 | [10241](http://www.ncbi.nlm.nih.gov/entrez/query.fcgi?db=gene&cmd=Retrieve&dopt=full_report&list_uids=10241) |
| UL38 | CCNDBP1 | cyclin D-type binding-protein 1 | [23582](http://www.ncbi.nlm.nih.gov/entrez/query.fcgi?db=gene&cmd=Retrieve&dopt=full_report&list_uids=23582) |
| UL36 | KRTAP4-12 | keratin associated protein 4-12 | [83755](http://www.ncbi.nlm.nih.gov/entrez/query.fcgi?db=gene&cmd=Retrieve&dopt=full_report&list_uids=83755) |
| UL38 | PNMA1 | paraneoplastic antigen MA1 | [9240](http://www.ncbi.nlm.nih.gov/entrez/query.fcgi?db=gene&cmd=Retrieve&dopt=full_report&list_uids=9240) |
| UL43 | KRTAP4-12 | keratin associated protein 4-12 | [83755](http://www.ncbi.nlm.nih.gov/entrez/query.fcgi?db=gene&cmd=Retrieve&dopt=full_report&list_uids=83755) |
| UL44 | ELAVL3 | ELAV (embryonic lethal, abnormal vision, Drosophila)-like 3 (Hu antigen C) | [1995](http://www.ncbi.nlm.nih.gov/entrez/query.fcgi?db=gene&cmd=Retrieve&dopt=full_report&list_uids=1995) |
| UL44 | ELAVL2 | ELAV (embryonic lethal, abnormal vision, Drosophila)-like 2 (Hu antigen B) | [1993](http://www.ncbi.nlm.nih.gov/entrez/query.fcgi?db=gene&cmd=Retrieve&dopt=full_report&list_uids=1993) |
| UL56 | CCNDBP1 | cyclin D-type binding-protein 1 | [23582](http://www.ncbi.nlm.nih.gov/entrez/query.fcgi?db=gene&cmd=Retrieve&dopt=full_report&list_uids=23582) |
| UL56 | SF3B4 | splicing factor 3b, subunit 4, 49kDa | [10262](http://www.ncbi.nlm.nih.gov/entrez/query.fcgi?db=gene&cmd=Retrieve&dopt=full_report&list_uids=10262) |
| UL46 | SF3B4 | splicing factor 3b, subunit 4, 49kDa | [10262](http://www.ncbi.nlm.nih.gov/entrez/query.fcgi?db=gene&cmd=Retrieve&dopt=full_report&list_uids=10262) |
| US8 | CCDC33 | coiled-coil domain containing 33 | [80125](http://www.ncbi.nlm.nih.gov/entrez/query.fcgi?db=gene&cmd=Retrieve&dopt=full_report&list_uids=80125) |
| RL2 | CCDC33 | coiled-coil domain containing 33 | [80125](http://www.ncbi.nlm.nih.gov/entrez/query.fcgi?db=gene&cmd=Retrieve&dopt=full_report&list_uids=80125) |
| UL10 | CCDC33 | coiled-coil domain containing 33 | [80125](http://www.ncbi.nlm.nih.gov/entrez/query.fcgi?db=gene&cmd=Retrieve&dopt=full_report&list_uids=80125) |
| **Low Confidence interactions** | | | |
| RL2 | DPPA4 | developmental pluripotency associated 4 | [55211](http://www.ncbi.nlm.nih.gov/entrez/query.fcgi?db=gene&cmd=Retrieve&dopt=full_report&list_uids=55211) |
| RL2 | UBE2V1 | ubiquitin-conjugating enzyme E2 variant 1 | [7335](http://www.ncbi.nlm.nih.gov/entrez/query.fcgi?db=gene&cmd=Retrieve&dopt=full_report&list_uids=7335) |
| UL10 | CREB3L1 | cAMP responsive element binding protein 3-like 1 | [90993](http://www.ncbi.nlm.nih.gov/entrez/query.fcgi?db=gene&cmd=Retrieve&dopt=full_report&list_uids=90993) |
| UL2 | PCDHGC5 | protocadherin gamma subfamily C, 5 | [56097](http://www.ncbi.nlm.nih.gov/entrez/query.fcgi?db=gene&cmd=Retrieve&dopt=full_report&list_uids=56097) |
| UL20 | C14orf2 | chromosome 14 open reading frame 2 | [9556](http://www.ncbi.nlm.nih.gov/entrez/query.fcgi?db=gene&cmd=Retrieve&dopt=full_report&list_uids=9556) |
| UL20 | C4orf3 | chromosome 4 open reading frame 3 | [401152](http://www.ncbi.nlm.nih.gov/entrez/query.fcgi?db=gene&cmd=Retrieve&dopt=full_report&list_uids=401152) |
| UL20 | C9orf85 | chromosome 9 open reading frame 85 | [138241](http://www.ncbi.nlm.nih.gov/entrez/query.fcgi?db=gene&cmd=Retrieve&dopt=full_report&list_uids=138241) |
| UL20 | CYTH3 | cytohesin 3 | [9265](http://www.ncbi.nlm.nih.gov/entrez/query.fcgi?db=gene&cmd=Retrieve&dopt=full_report&list_uids=9265) |
| UL20 | DRAM1 | DNA-damage regulated autophagy modulator 1 | [55332](http://www.ncbi.nlm.nih.gov/entrez/query.fcgi?db=gene&cmd=Retrieve&dopt=full_report&list_uids=55332) |
| UL20 | EEF1G | eukaryotic translation elongation factor 1 gamma | [1937](http://www.ncbi.nlm.nih.gov/entrez/query.fcgi?db=gene&cmd=Retrieve&dopt=full_report&list_uids=1937) |
| UL20 | EIF5A | eukaryotic translation initiation factor 5A | [1984](http://www.ncbi.nlm.nih.gov/entrez/query.fcgi?db=gene&cmd=Retrieve&dopt=full_report&list_uids=1984) |
| UL20 | FAM107A | family with sequence similarity 107, member A | [11170](http://www.ncbi.nlm.nih.gov/entrez/query.fcgi?db=gene&cmd=Retrieve&dopt=full_report&list_uids=11170) |
| UL20 | FGFBP1 | fibroblast growth factor binding protein 1 | [9982](http://www.ncbi.nlm.nih.gov/entrez/query.fcgi?db=gene&cmd=Retrieve&dopt=full_report&list_uids=9982) |
| UL20 | GJA5 | gap junction protein, alpha 5, 40kDa | [2702](http://www.ncbi.nlm.nih.gov/entrez/query.fcgi?db=gene&cmd=Retrieve&dopt=full_report&list_uids=2702) |
| UL20 | GP9 | glycoprotein IX (platelet) | [2815](http://www.ncbi.nlm.nih.gov/entrez/query.fcgi?db=gene&cmd=Retrieve&dopt=full_report&list_uids=2815) |
| UL20 | GYPB | glycophorin B (MNS blood group) | [2994](http://www.ncbi.nlm.nih.gov/entrez/query.fcgi?db=gene&cmd=Retrieve&dopt=full_report&list_uids=2994) |
| UL20 | HCG9 | HLA complex group 9 | [10255](http://www.ncbi.nlm.nih.gov/entrez/query.fcgi?db=gene&cmd=Retrieve&dopt=full_report&list_uids=10255) |
| UL20 | HGF | hepatocyte growth factor (hepapoietin A; scatter factor) | [3082](http://www.ncbi.nlm.nih.gov/entrez/query.fcgi?db=gene&cmd=Retrieve&dopt=full_report&list_uids=3082) |
| UL20 | HMGN1 | high-mobility group nucleosome binding domain 1 | [3150](http://www.ncbi.nlm.nih.gov/entrez/query.fcgi?db=gene&cmd=Retrieve&dopt=full_report&list_uids=3150) |
| UL20 | HOXA10 | homeobox A10 | [3206](http://www.ncbi.nlm.nih.gov/entrez/query.fcgi?db=gene&cmd=Retrieve&dopt=full_report&list_uids=3206) |
| UL20 | INSIG1 | insulin induced gene 1 | [3638](http://www.ncbi.nlm.nih.gov/entrez/query.fcgi?db=gene&cmd=Retrieve&dopt=full_report&list_uids=3638) |
| UL20 | LDLRAP1 | low density lipoprotein receptor adaptor protein 1 | [26119](http://www.ncbi.nlm.nih.gov/entrez/query.fcgi?db=gene&cmd=Retrieve&dopt=full_report&list_uids=26119) |
| UL20 | MAP2K1 | mitogen-activated protein kinase kinase 1 | [5604](http://www.ncbi.nlm.nih.gov/entrez/query.fcgi?db=gene&cmd=Retrieve&dopt=full_report&list_uids=5604) |
| UL20 | MRAS | muscle RAS oncogene homolog | [22808](http://www.ncbi.nlm.nih.gov/entrez/query.fcgi?db=gene&cmd=Retrieve&dopt=full_report&list_uids=22808) |
| UL20 | NSUN5C | NOL1/NOP2/Sun domain family, member 5C | [260294](http://www.ncbi.nlm.nih.gov/entrez/query.fcgi?db=gene&cmd=Retrieve&dopt=full_report&list_uids=260294) |
| UL20 | OPRM1 | opioid receptor, mu 1 | [4988](http://www.ncbi.nlm.nih.gov/entrez/query.fcgi?db=gene&cmd=Retrieve&dopt=full_report&list_uids=4988) |
| UL20 | ORM2 | orosomucoid 2 | [5005](http://www.ncbi.nlm.nih.gov/entrez/query.fcgi?db=gene&cmd=Retrieve&dopt=full_report&list_uids=5005) |
| UL20 | OSTF1 | osteoclast stimulating factor 1 | [26578](http://www.ncbi.nlm.nih.gov/entrez/query.fcgi?db=gene&cmd=Retrieve&dopt=full_report&list_uids=26578) |
| UL20 | PLIN2 | perilipin 2 | [123](http://www.ncbi.nlm.nih.gov/entrez/query.fcgi?db=gene&cmd=Retrieve&dopt=full_report&list_uids=123) |
| UL20 | POLR1D | polymerase (RNA) I polypeptide D, 16kDa | [51082](http://www.ncbi.nlm.nih.gov/entrez/query.fcgi?db=gene&cmd=Retrieve&dopt=full_report&list_uids=51082) |
| UL20 | RAB6B | RAB6B, member RAS oncogene family | [51560](http://www.ncbi.nlm.nih.gov/entrez/query.fcgi?db=gene&cmd=Retrieve&dopt=full_report&list_uids=51560) |
| UL20 | RDH11 | retinol dehydrogenase 11 (all-trans/9-cis/11-cis) | [51109](http://www.ncbi.nlm.nih.gov/entrez/query.fcgi?db=gene&cmd=Retrieve&dopt=full_report&list_uids=51109) |
| UL20 | RPS14 | ribosomal protein S14 | [6208](http://www.ncbi.nlm.nih.gov/entrez/query.fcgi?db=gene&cmd=Retrieve&dopt=full_report&list_uids=6208) |
| UL20 | SCNM1 | sodium channel modifier 1 | [79005](http://www.ncbi.nlm.nih.gov/entrez/query.fcgi?db=gene&cmd=Retrieve&dopt=full_report&list_uids=79005) |
| UL20 | SPEF1 | sperm flagellar 1 | [25876](http://www.ncbi.nlm.nih.gov/entrez/query.fcgi?db=gene&cmd=Retrieve&dopt=full_report&list_uids=25876) |
| UL20 | SPINK6 | serine peptidase inhibitor, Kazal type 6 | [404203](http://www.ncbi.nlm.nih.gov/entrez/query.fcgi?db=gene&cmd=Retrieve&dopt=full_report&list_uids=404203) |
| UL20 | SRP9L1 | signal recognition particle 9-like 1 | [414307](http://www.ncbi.nlm.nih.gov/entrez/query.fcgi?db=gene&cmd=Retrieve&dopt=full_report&list_uids=414307) |
| UL20 | TMEM167A | transmembrane protein 167A | [153339](http://www.ncbi.nlm.nih.gov/entrez/query.fcgi?db=gene&cmd=Retrieve&dopt=full_report&list_uids=153339) |
| UL20 | TMEM182 | transmembrane protein 182 | [130827](http://www.ncbi.nlm.nih.gov/entrez/query.fcgi?db=gene&cmd=Retrieve&dopt=full_report&list_uids=130827) |
| UL20 | TMEM206 | transmembrane protein 206 | [55248](http://www.ncbi.nlm.nih.gov/entrez/query.fcgi?db=gene&cmd=Retrieve&dopt=full_report&list_uids=55248) |
| UL20 | TNFSF14 | tumor necrosis factor (ligand) superfamily, member 14 | [8740](http://www.ncbi.nlm.nih.gov/entrez/query.fcgi?db=gene&cmd=Retrieve&dopt=full_report&list_uids=8740) |
| UL20 | UNC119 | unc-119 homolog (C. elegans) | [9094](http://www.ncbi.nlm.nih.gov/entrez/query.fcgi?db=gene&cmd=Retrieve&dopt=full_report&list_uids=9094) |
| UL20 | VASH1 | vasohibin 1 | [22846](http://www.ncbi.nlm.nih.gov/entrez/query.fcgi?db=gene&cmd=Retrieve&dopt=full_report&list_uids=22846) |
| UL20 | ZDHHC7 | zinc finger, DHHC-type containing 7 | [55625](http://www.ncbi.nlm.nih.gov/entrez/query.fcgi?db=gene&cmd=Retrieve&dopt=full_report&list_uids=55625) |
| UL21 | NR2C1 | nuclear receptor subfamily 2, group C, member 1 | [7181](http://www.ncbi.nlm.nih.gov/entrez/query.fcgi?db=gene&cmd=Retrieve&dopt=full_report&list_uids=7181) |
| UL21 | NUP35 | nucleoporin 35kDa | [129401](http://www.ncbi.nlm.nih.gov/entrez/query.fcgi?db=gene&cmd=Retrieve&dopt=full_report&list_uids=129401) |
| UL22 | C12orf23 | chromosome 12 open reading frame 23 | [90488](http://www.ncbi.nlm.nih.gov/entrez/query.fcgi?db=gene&cmd=Retrieve&dopt=full_report&list_uids=90488) |
| UL22 | CCDC89 | coiled-coil domain containing 89 | [220388](http://www.ncbi.nlm.nih.gov/entrez/query.fcgi?db=gene&cmd=Retrieve&dopt=full_report&list_uids=220388) |
| UL22 | CCL18 | chemokine (C-C motif) ligand 18 (pulmonary and activation-regulated) | [6362](http://www.ncbi.nlm.nih.gov/entrez/query.fcgi?db=gene&cmd=Retrieve&dopt=full_report&list_uids=6362) |
| UL22 | CDH19 | cadherin 19, type 2 | [28513](http://www.ncbi.nlm.nih.gov/entrez/query.fcgi?db=gene&cmd=Retrieve&dopt=full_report&list_uids=28513) |
| UL22 | EIF3K | eukaryotic translation initiation factor 3, subunit K | [27335](http://www.ncbi.nlm.nih.gov/entrez/query.fcgi?db=gene&cmd=Retrieve&dopt=full_report&list_uids=27335) |
| UL22 | EXT1 | exostoses (multiple) 1 | [2131](http://www.ncbi.nlm.nih.gov/entrez/query.fcgi?db=gene&cmd=Retrieve&dopt=full_report&list_uids=2131) |
| UL22 | FA2H | fatty acid 2-hydroxylase | [79152](http://www.ncbi.nlm.nih.gov/entrez/query.fcgi?db=gene&cmd=Retrieve&dopt=full_report&list_uids=79152) |
| UL22 | IFI27L1 | interferon, alpha-inducible protein 27-like 1 | [122509](http://www.ncbi.nlm.nih.gov/entrez/query.fcgi?db=gene&cmd=Retrieve&dopt=full_report&list_uids=122509) |
| UL22 | LOC100293440 | similar to Ig lambda chain | [100293440](http://www.ncbi.nlm.nih.gov/entrez/query.fcgi?db=gene&cmd=Retrieve&dopt=full_report&list_uids=100293440) |
| UL22 | LOC400713 | zinc finger-like | [400713](http://www.ncbi.nlm.nih.gov/entrez/query.fcgi?db=gene&cmd=Retrieve&dopt=full_report&list_uids=400713) |
| UL22 | MRPS27 | mitochondrial ribosomal protein S27 | [23107](http://www.ncbi.nlm.nih.gov/entrez/query.fcgi?db=gene&cmd=Retrieve&dopt=full_report&list_uids=23107) |
| UL22 | OLAH | oleoyl-ACP hydrolase | [55301](http://www.ncbi.nlm.nih.gov/entrez/query.fcgi?db=gene&cmd=Retrieve&dopt=full_report&list_uids=55301) |
| UL22 | OSTC | oligosaccharyltransferase complex subunit | [58505](http://www.ncbi.nlm.nih.gov/entrez/query.fcgi?db=gene&cmd=Retrieve&dopt=full_report&list_uids=58505) |
| UL22 | PGF | placental growth factor | [5228](http://www.ncbi.nlm.nih.gov/entrez/query.fcgi?db=gene&cmd=Retrieve&dopt=full_report&list_uids=5228) |
| UL22 | PLEK | pleckstrin | [5341](http://www.ncbi.nlm.nih.gov/entrez/query.fcgi?db=gene&cmd=Retrieve&dopt=full_report&list_uids=5341) |
| UL22 | RHEB | Ras homolog enriched in brain | [6009](http://www.ncbi.nlm.nih.gov/entrez/query.fcgi?db=gene&cmd=Retrieve&dopt=full_report&list_uids=6009) |
| UL22 | RPL28 | ribosomal protein L28 | [6158](http://www.ncbi.nlm.nih.gov/entrez/query.fcgi?db=gene&cmd=Retrieve&dopt=full_report&list_uids=6158) |
| UL22 | S100A16 | S100 calcium binding protein A16 | [140576](http://www.ncbi.nlm.nih.gov/entrez/query.fcgi?db=gene&cmd=Retrieve&dopt=full_report&list_uids=140576) |
| UL22 | SCG5 | secretogranin V (7B2 protein) | [6447](http://www.ncbi.nlm.nih.gov/entrez/query.fcgi?db=gene&cmd=Retrieve&dopt=full_report&list_uids=6447) |
| UL22 | SNX15 | sorting nexin 15 | [29907](http://www.ncbi.nlm.nih.gov/entrez/query.fcgi?db=gene&cmd=Retrieve&dopt=full_report&list_uids=29907) |
| UL22 | SS18L1 | synovial sarcoma translocation gene on chromosome 18-like 1 | [26039](http://www.ncbi.nlm.nih.gov/entrez/query.fcgi?db=gene&cmd=Retrieve&dopt=full_report&list_uids=26039) |
| UL22 | UBE2L6 | ubiquitin-conjugating enzyme E2L 6 | [9246](http://www.ncbi.nlm.nih.gov/entrez/query.fcgi?db=gene&cmd=Retrieve&dopt=full_report&list_uids=9246) |
| UL28 | POU2AF1 | POU class 2 associating factor 1 | [5450](http://www.ncbi.nlm.nih.gov/entrez/query.fcgi?db=gene&cmd=Retrieve&dopt=full_report&list_uids=5450) |
| UL31 | LHX8 | LIM homeobox 8 | [431707](http://www.ncbi.nlm.nih.gov/entrez/query.fcgi?db=gene&cmd=Retrieve&dopt=full_report&list_uids=431707) |
| UL40 | C21orf121 | chromosome 21 open reading frame 121 | [150142](http://www.ncbi.nlm.nih.gov/entrez/query.fcgi?db=gene&cmd=Retrieve&dopt=full_report&list_uids=150142) |
| UL40 | CENPQ | centromere protein Q | [55166](http://www.ncbi.nlm.nih.gov/entrez/query.fcgi?db=gene&cmd=Retrieve&dopt=full_report&list_uids=55166) |
| UL40 | CREBL2 | cAMP responsive element binding protein-like 2 | [1389](http://www.ncbi.nlm.nih.gov/entrez/query.fcgi?db=gene&cmd=Retrieve&dopt=full_report&list_uids=1389) |
| UL40 | DEFB1 | defensin, beta 1 | [1672](http://www.ncbi.nlm.nih.gov/entrez/query.fcgi?db=gene&cmd=Retrieve&dopt=full_report&list_uids=1672) |
| UL40 | HPGDS | hematopoietic prostaglandin D synthase | [27306](http://www.ncbi.nlm.nih.gov/entrez/query.fcgi?db=gene&cmd=Retrieve&dopt=full_report&list_uids=27306) |
| UL40 | SLC16A10 | solute carrier family 16, member 10 (aromatic amino acid transporter) | [117247](http://www.ncbi.nlm.nih.gov/entrez/query.fcgi?db=gene&cmd=Retrieve&dopt=full_report&list_uids=117247) |
| UL43 | ASGR2 | asialoglycoprotein receptor 2 | [433](http://www.ncbi.nlm.nih.gov/entrez/query.fcgi?db=gene&cmd=Retrieve&dopt=full_report&list_uids=433) |
| UL44 | IRF1 | interferon regulatory factor 1 | [3659](http://www.ncbi.nlm.nih.gov/entrez/query.fcgi?db=gene&cmd=Retrieve&dopt=full_report&list_uids=3659) |
| UL45 | IL17RA | interleukin 17 receptor A | [23765](http://www.ncbi.nlm.nih.gov/entrez/query.fcgi?db=gene&cmd=Retrieve&dopt=full_report&list_uids=23765) |
| UL45 | LOC100287323 | hypothetical protein LOC100287323 | [100287323](http://www.ncbi.nlm.nih.gov/entrez/query.fcgi?db=gene&cmd=Retrieve&dopt=full_report&list_uids=100287323) |
| UL45 | LOC100291034 | hypothetical protein LOC100291034 | [100291034](http://www.ncbi.nlm.nih.gov/entrez/query.fcgi?db=gene&cmd=Retrieve&dopt=full_report&list_uids=100291034) |
| UL45 | LOC100292558 | hypothetical protein LOC100292558 | [100292558](http://www.ncbi.nlm.nih.gov/entrez/query.fcgi?db=gene&cmd=Retrieve&dopt=full_report&list_uids=100292558) |
| UL46 | NFE2 | nuclear factor (erythroid-derived 2), 45kDa | [4778](http://www.ncbi.nlm.nih.gov/entrez/query.fcgi?db=gene&cmd=Retrieve&dopt=full_report&list_uids=4778) |
| UL46 | TNFAIP6 | tumor necrosis factor, alpha-induced protein 6 | [7130](http://www.ncbi.nlm.nih.gov/entrez/query.fcgi?db=gene&cmd=Retrieve&dopt=full_report&list_uids=7130) |
| UL46 | TRAF4 | TNF receptor-associated factor 4 | [9618](http://www.ncbi.nlm.nih.gov/entrez/query.fcgi?db=gene&cmd=Retrieve&dopt=full_report&list_uids=9618) |
| UL46 | VPS37B | vacuolar protein sorting 37 homolog B (S. cerevisiae) | [79720](http://www.ncbi.nlm.nih.gov/entrez/query.fcgi?db=gene&cmd=Retrieve&dopt=full_report&list_uids=79720) |
| UL46 | ZC3H12A | zinc finger CCCH-type containing 12A | [80149](http://www.ncbi.nlm.nih.gov/entrez/query.fcgi?db=gene&cmd=Retrieve&dopt=full_report&list_uids=80149) |
| UL49 | C3orf25 | chromosome 3 open reading frame 25 | [90288](http://www.ncbi.nlm.nih.gov/entrez/query.fcgi?db=gene&cmd=Retrieve&dopt=full_report&list_uids=90288) |
| UL54 | CCDC36 | coiled-coil domain containing 36 | [339834](http://www.ncbi.nlm.nih.gov/entrez/query.fcgi?db=gene&cmd=Retrieve&dopt=full_report&list_uids=339834) |
| UL55 | L3MBTL3 | l(3)mbt-like 3 (Drosophila) | [84456](http://www.ncbi.nlm.nih.gov/entrez/query.fcgi?db=gene&cmd=Retrieve&dopt=full_report&list_uids=84456) |
| UL56 | PIM2 | pim-2 oncogene | [11040](http://www.ncbi.nlm.nih.gov/entrez/query.fcgi?db=gene&cmd=Retrieve&dopt=full_report&list_uids=11040) |
| UL56 | SNRPE | small nuclear ribonucleoprotein polypeptide E | [6635](http://www.ncbi.nlm.nih.gov/entrez/query.fcgi?db=gene&cmd=Retrieve&dopt=full_report&list_uids=6635) |
| UL56 | TOMM5 | translocase of outer mitochondrial membrane 5 homolog (yeast) | [401505](http://www.ncbi.nlm.nih.gov/entrez/query.fcgi?db=gene&cmd=Retrieve&dopt=full_report&list_uids=401505) |
| UL7 | ARAF | v-raf murine sarcoma 3611 viral oncogene homolog | [369](http://www.ncbi.nlm.nih.gov/entrez/query.fcgi?db=gene&cmd=Retrieve&dopt=full_report&list_uids=369) |
| UL7 | ATP6V1C2 | ATPase, H+ transporting, lysosomal 42kDa, V1 subunit C2 | [245973](http://www.ncbi.nlm.nih.gov/entrez/query.fcgi?db=gene&cmd=Retrieve&dopt=full_report&list_uids=245973) |
| UL7 | CLEC2B | C-type lectin domain family 2, member B | [9976](http://www.ncbi.nlm.nih.gov/entrez/query.fcgi?db=gene&cmd=Retrieve&dopt=full_report&list_uids=9976) |
| UL7 | EDNRA | endothelin receptor type A | [1909](http://www.ncbi.nlm.nih.gov/entrez/query.fcgi?db=gene&cmd=Retrieve&dopt=full_report&list_uids=1909) |
| UL7 | FAM69A | family with sequence similarity 69, member A | [388650](http://www.ncbi.nlm.nih.gov/entrez/query.fcgi?db=gene&cmd=Retrieve&dopt=full_report&list_uids=388650) |
| UL7 | GABRA3 | gamma-aminobutyric acid (GABA) A receptor, alpha 3 | [2556](http://www.ncbi.nlm.nih.gov/entrez/query.fcgi?db=gene&cmd=Retrieve&dopt=full_report&list_uids=2556) |
| UL7 | GNA11 | guanine nucleotide binding protein (G protein), alpha 11 (Gq class) | [2767](http://www.ncbi.nlm.nih.gov/entrez/query.fcgi?db=gene&cmd=Retrieve&dopt=full_report&list_uids=2767) |
| UL7 | HNRNPUL1 | heterogeneous nuclear ribonucleoprotein U-like 1 | [11100](http://www.ncbi.nlm.nih.gov/entrez/query.fcgi?db=gene&cmd=Retrieve&dopt=full_report&list_uids=11100) |
| UL7 | KDM1 | KDM1 lysine (K)-specific demethylase 1 [ Homo sapiens ] | [23028](http://www.ncbi.nlm.nih.gov/entrez/query.fcgi?db=gene&cmd=Retrieve&dopt=full_report&list_uids=23028) |
| UL7 | KRT6B | keratin 6B | [3854](http://www.ncbi.nlm.nih.gov/entrez/query.fcgi?db=gene&cmd=Retrieve&dopt=full_report&list_uids=3854) |
| UL7 | MRS2 | MRS2 magnesium homeostasis factor homolog (S. cerevisiae) | [57380](http://www.ncbi.nlm.nih.gov/entrez/query.fcgi?db=gene&cmd=Retrieve&dopt=full_report&list_uids=57380) |
| UL7 | TRAF6 | TNF receptor-associated factor 6 | [7189](http://www.ncbi.nlm.nih.gov/entrez/query.fcgi?db=gene&cmd=Retrieve&dopt=full_report&list_uids=7189) |
| UL7 | U2AF1 | U2 small nuclear RNA auxiliary factor 1 | [7307](http://www.ncbi.nlm.nih.gov/entrez/query.fcgi?db=gene&cmd=Retrieve&dopt=full_report&list_uids=7307) |
| UL7 | ZNF626 | zinc finger protein 626 | [199777](http://www.ncbi.nlm.nih.gov/entrez/query.fcgi?db=gene&cmd=Retrieve&dopt=full_report&list_uids=199777) |
| US1 | ZNF24 | zinc finger protein 24 | [7572](http://www.ncbi.nlm.nih.gov/entrez/query.fcgi?db=gene&cmd=Retrieve&dopt=full_report&list_uids=7572) |
| RL2 | ZSCAN21 | zinc finger and SCAN domain containing 21 | [7589](http://www.ncbi.nlm.nih.gov/entrez/query.fcgi?db=gene&cmd=Retrieve&dopt=full_report&list_uids=7589) |
| UL20 | ANKRD39 | ankyrin repeat domain 39 | [51239](http://www.ncbi.nlm.nih.gov/entrez/query.fcgi?db=gene&cmd=Retrieve&dopt=full_report&list_uids=51239) |
| UL20 | ATP6V1F | ATPase, H+ transporting, lysosomal 14kDa, V1 subunit F | [9296](http://www.ncbi.nlm.nih.gov/entrez/query.fcgi?db=gene&cmd=Retrieve&dopt=full_report&list_uids=9296) |
| UL20 | CCL19 | chemokine (C-C motif) ligand 19 | [6363](http://www.ncbi.nlm.nih.gov/entrez/query.fcgi?db=gene&cmd=Retrieve&dopt=full_report&list_uids=6363) |
| UL20 | CCL21 | chemokine (C-C motif) ligand 21 | [6366](http://www.ncbi.nlm.nih.gov/entrez/query.fcgi?db=gene&cmd=Retrieve&dopt=full_report&list_uids=6366) |
| UL20 | EWSR1 | Ewing sarcoma breakpoint region 1 | [2130](http://www.ncbi.nlm.nih.gov/entrez/query.fcgi?db=gene&cmd=Retrieve&dopt=full_report&list_uids=2130) |
| UL20 | FOLR1 | folate receptor 1 (adult) | [2348](http://www.ncbi.nlm.nih.gov/entrez/query.fcgi?db=gene&cmd=Retrieve&dopt=full_report&list_uids=2348) |
| UL20 | HBG2 | hemoglobin, gamma G | [3048](http://www.ncbi.nlm.nih.gov/entrez/query.fcgi?db=gene&cmd=Retrieve&dopt=full_report&list_uids=3048) |
| UL20 | IFITM1 | interferon induced transmembrane protein 1 (9-27) | [8519](http://www.ncbi.nlm.nih.gov/entrez/query.fcgi?db=gene&cmd=Retrieve&dopt=full_report&list_uids=8519) |
| UL20 | NUDT3 | nudix (nucleoside diphosphate linked moiety X)-type motif 3 | [11165](http://www.ncbi.nlm.nih.gov/entrez/query.fcgi?db=gene&cmd=Retrieve&dopt=full_report&list_uids=11165) |
| UL20 | PLA2G2A | phospholipase A2, group IIA (platelets, synovial fluid) | [5320](http://www.ncbi.nlm.nih.gov/entrez/query.fcgi?db=gene&cmd=Retrieve&dopt=full_report&list_uids=5320) |
| UL20 | TPSAB1 | tryptase alpha/beta 1 | [7177](http://www.ncbi.nlm.nih.gov/entrez/query.fcgi?db=gene&cmd=Retrieve&dopt=full_report&list_uids=7177) |
| UL21 | ZNF446 | zinc finger protein 446 | [55663](http://www.ncbi.nlm.nih.gov/entrez/query.fcgi?db=gene&cmd=Retrieve&dopt=full_report&list_uids=55663) |
| UL22 | ANKRD39 | ankyrin repeat domain 39 | [51239](http://www.ncbi.nlm.nih.gov/entrez/query.fcgi?db=gene&cmd=Retrieve&dopt=full_report&list_uids=51239) |
| UL22 | FOLR1 | folate receptor 1 (adult) | [2348](http://www.ncbi.nlm.nih.gov/entrez/query.fcgi?db=gene&cmd=Retrieve&dopt=full_report&list_uids=2348) |
| UL22 | HSPBP1 | hsp70-interacting protein | [23640](http://www.ncbi.nlm.nih.gov/entrez/query.fcgi?db=gene&cmd=Retrieve&dopt=full_report&list_uids=23640) |
| UL22 | PDCD10 | programmed cell death 10 | [11235](http://www.ncbi.nlm.nih.gov/entrez/query.fcgi?db=gene&cmd=Retrieve&dopt=full_report&list_uids=11235) |
| UL22 | PIGX | phosphatidylinositol glycan anchor biosynthesis, class X | [54965](http://www.ncbi.nlm.nih.gov/entrez/query.fcgi?db=gene&cmd=Retrieve&dopt=full_report&list_uids=54965) |
| UL22 | RBP5 | retinol binding protein 5, cellular | [83758](http://www.ncbi.nlm.nih.gov/entrez/query.fcgi?db=gene&cmd=Retrieve&dopt=full_report&list_uids=83758) |
| UL33 | COMMD4 | COMM domain containing 4 | [54939](http://www.ncbi.nlm.nih.gov/entrez/query.fcgi?db=gene&cmd=Retrieve&dopt=full_report&list_uids=54939) |
| UL34 | BEND5 | BEN domain containing 5 | [79656](http://www.ncbi.nlm.nih.gov/entrez/query.fcgi?db=gene&cmd=Retrieve&dopt=full_report&list_uids=79656) |
| UL38 | MTUS2 | microtubule associated tumor suppressor candidate 2 | [23281](http://www.ncbi.nlm.nih.gov/entrez/query.fcgi?db=gene&cmd=Retrieve&dopt=full_report&list_uids=23281) |
| UL40 | BEND5 | BEN domain containing 5 | [79656](http://www.ncbi.nlm.nih.gov/entrez/query.fcgi?db=gene&cmd=Retrieve&dopt=full_report&list_uids=79656) |
| UL40 | DNAJC16 | DnaJ (Hsp40) homolog, subfamily C, member 16 | [23341](http://www.ncbi.nlm.nih.gov/entrez/query.fcgi?db=gene&cmd=Retrieve&dopt=full_report&list_uids=23341) |
| UL40 | GNG7 | guanine nucleotide binding protein (G protein), gamma 7 | [2788](http://www.ncbi.nlm.nih.gov/entrez/query.fcgi?db=gene&cmd=Retrieve&dopt=full_report&list_uids=2788) |
| UL40 | ING5 | inhibitor of growth family, member 5 | [84289](http://www.ncbi.nlm.nih.gov/entrez/query.fcgi?db=gene&cmd=Retrieve&dopt=full_report&list_uids=84289) |
| UL40 | STX11 | syntaxin 11 | [8676](http://www.ncbi.nlm.nih.gov/entrez/query.fcgi?db=gene&cmd=Retrieve&dopt=full_report&list_uids=8676) |
| UL44 | NME1 | non-metastatic cells 1, protein (NM23A) expressed in | [4830](http://www.ncbi.nlm.nih.gov/entrez/query.fcgi?db=gene&cmd=Retrieve&dopt=full_report&list_uids=4830) |
| UL44 | TSC22D4 | TSC22 domain family, member 4 | [81628](http://www.ncbi.nlm.nih.gov/entrez/query.fcgi?db=gene&cmd=Retrieve&dopt=full_report&list_uids=81628) |
| UL46 | CSRNP1 | cysteine-serine-rich nuclear protein 1 | [64651](http://www.ncbi.nlm.nih.gov/entrez/query.fcgi?db=gene&cmd=Retrieve&dopt=full_report&list_uids=64651) |
| UL46 | FOS | v-fos FBJ murine osteosarcoma viral oncogene homolog | [2353](http://www.ncbi.nlm.nih.gov/entrez/query.fcgi?db=gene&cmd=Retrieve&dopt=full_report&list_uids=2353) |
| UL46 | MLLT6 | myeloid/lymphoid or mixed-lineage leukemia (trithorax homolog, Drosophila); translocated to, 6 | [4302](http://www.ncbi.nlm.nih.gov/entrez/query.fcgi?db=gene&cmd=Retrieve&dopt=full_report&list_uids=4302) |
| UL46 | MTUS2 | microtubule associated tumor suppressor candidate 2 | [23281](http://www.ncbi.nlm.nih.gov/entrez/query.fcgi?db=gene&cmd=Retrieve&dopt=full_report&list_uids=23281) |
| UL46 | RAB20 | RAB20, member RAS oncogene family | [55647](http://www.ncbi.nlm.nih.gov/entrez/query.fcgi?db=gene&cmd=Retrieve&dopt=full_report&list_uids=55647) |
| UL46 | WBP11 | WW domain binding protein 11 | [51729](http://www.ncbi.nlm.nih.gov/entrez/query.fcgi?db=gene&cmd=Retrieve&dopt=full_report&list_uids=51729) |
| UL47 | SYNJ2BP | synaptojanin 2 binding protein | [55333](http://www.ncbi.nlm.nih.gov/entrez/query.fcgi?db=gene&cmd=Retrieve&dopt=full_report&list_uids=55333) |
| UL49 | PFKM | phosphofructokinase, muscle | [5213](http://www.ncbi.nlm.nih.gov/entrez/query.fcgi?db=gene&cmd=Retrieve&dopt=full_report&list_uids=5213) |
| UL49 | PRPF38B | PRP38 pre-mRNA processing factor 38 (yeast) domain containing B | [55119](http://www.ncbi.nlm.nih.gov/entrez/query.fcgi?db=gene&cmd=Retrieve&dopt=full_report&list_uids=55119) |
| UL53 | MYST2 | MYST histone acetyltransferase 2 | [11143](http://www.ncbi.nlm.nih.gov/entrez/query.fcgi?db=gene&cmd=Retrieve&dopt=full_report&list_uids=11143) |
| UL7 | DEDD2 | death effector domain containing 2 | [162989](http://www.ncbi.nlm.nih.gov/entrez/query.fcgi?db=gene&cmd=Retrieve&dopt=full_report&list_uids=162989) |
| UL7 | GKN1 | gastrokine 1 | [56287](http://www.ncbi.nlm.nih.gov/entrez/query.fcgi?db=gene&cmd=Retrieve&dopt=full_report&list_uids=56287) |
| UL7 | IFIT1 | interferon-induced protein with tetratricopeptide repeats 1 | [3434](http://www.ncbi.nlm.nih.gov/entrez/query.fcgi?db=gene&cmd=Retrieve&dopt=full_report&list_uids=3434) |
| US1 | CDYL2 | chromodomain protein, Y-like 2 | [124359](http://www.ncbi.nlm.nih.gov/entrez/query.fcgi?db=gene&cmd=Retrieve&dopt=full_report&list_uids=124359) |
| RL2 | ATP5J | ATP synthase, H+ transporting, mitochondrial F0 complex, subunit F6 | [522](http://www.ncbi.nlm.nih.gov/entrez/query.fcgi?db=gene&cmd=Retrieve&dopt=full_report&list_uids=522) |
| RL2 | GKAP1 | G kinase anchoring protein 1 | [80318](http://www.ncbi.nlm.nih.gov/entrez/query.fcgi?db=gene&cmd=Retrieve&dopt=full_report&list_uids=80318) |
| UL10 | TSR2 | TSR2, 20S rRNA accumulation, homolog (S. cerevisiae) | [90121](http://www.ncbi.nlm.nih.gov/entrez/query.fcgi?db=gene&cmd=Retrieve&dopt=full_report&list_uids=90121) |
| UL10 | ZNF496 | zinc finger protein 496 | [84838](http://www.ncbi.nlm.nih.gov/entrez/query.fcgi?db=gene&cmd=Retrieve&dopt=full_report&list_uids=84838) |
| UL17 | IKZF3 | IKAROS family zinc finger 3 (Aiolos) | [22806](http://www.ncbi.nlm.nih.gov/entrez/query.fcgi?db=gene&cmd=Retrieve&dopt=full_report&list_uids=22806) |
| UL20 | C10orf57 | chromosome 10 open reading frame 57 | [80195](http://www.ncbi.nlm.nih.gov/entrez/query.fcgi?db=gene&cmd=Retrieve&dopt=full_report&list_uids=80195) |
| UL20 | CCNK | cyclin K | [8812](http://www.ncbi.nlm.nih.gov/entrez/query.fcgi?db=gene&cmd=Retrieve&dopt=full_report&list_uids=8812) |
| UL20 | DPY30 | DPY30 dpy-30 homolog (C. elegans) [ Homo sapiens ] | [84661](http://www.ncbi.nlm.nih.gov/entrez/query.fcgi?db=gene&cmd=Retrieve&dopt=full_report&list_uids=84661) |
| UL20 | RPS17 | ribosomal protein S17 | [6218](http://www.ncbi.nlm.nih.gov/entrez/query.fcgi?db=gene&cmd=Retrieve&dopt=full_report&list_uids=6218) |
| UL20 | TMEM115 | transmembrane protein 115 | [11070](http://www.ncbi.nlm.nih.gov/entrez/query.fcgi?db=gene&cmd=Retrieve&dopt=full_report&list_uids=11070) |
| UL20 | TSPO | translocator protein (18kDa) | [706](http://www.ncbi.nlm.nih.gov/entrez/query.fcgi?db=gene&cmd=Retrieve&dopt=full_report&list_uids=706) |
| UL22 | BIRC5 | baculoviral IAP repeat-containing 5 (survivin) | [332](http://www.ncbi.nlm.nih.gov/entrez/query.fcgi?db=gene&cmd=Retrieve&dopt=full_report&list_uids=332) |
| UL22 | CPSF4 | cleavage and polyadenylation specific factor 4, 30kDa | [10898](http://www.ncbi.nlm.nih.gov/entrez/query.fcgi?db=gene&cmd=Retrieve&dopt=full_report&list_uids=10898) |
| UL22 | SPIN3 | spindlin family, member 3 | [169981](http://www.ncbi.nlm.nih.gov/entrez/query.fcgi?db=gene&cmd=Retrieve&dopt=full_report&list_uids=169981) |
| UL26 | HAUS6 | HAUS augmin-like complex, subunit 6 | [54801](http://www.ncbi.nlm.nih.gov/entrez/query.fcgi?db=gene&cmd=Retrieve&dopt=full_report&list_uids=54801) |
| UL27 | HAUS6 | HAUS augmin-like complex, subunit 6 | [54801](http://www.ncbi.nlm.nih.gov/entrez/query.fcgi?db=gene&cmd=Retrieve&dopt=full_report&list_uids=54801) |
| UL34 | KRT33B | keratin 33B | [3884](http://www.ncbi.nlm.nih.gov/entrez/query.fcgi?db=gene&cmd=Retrieve&dopt=full_report&list_uids=3884) |
| UL38 | IKBKG | inhibitor of kappa light polypeptide gene enhancer in B-cells, kinase gamma | [8517](http://www.ncbi.nlm.nih.gov/entrez/query.fcgi?db=gene&cmd=Retrieve&dopt=full_report&list_uids=8517) |
| UL39 | MAGEA6 | melanoma antigen family A, 6 | [4105](http://www.ncbi.nlm.nih.gov/entrez/query.fcgi?db=gene&cmd=Retrieve&dopt=full_report&list_uids=4105) |
| UL40 | CEP70 | centrosomal protein 70kDa | [80321](http://www.ncbi.nlm.nih.gov/entrez/query.fcgi?db=gene&cmd=Retrieve&dopt=full_report&list_uids=80321) |
| UL46 | RBM45 | RNA binding motif protein 45 | [129831](http://www.ncbi.nlm.nih.gov/entrez/query.fcgi?db=gene&cmd=Retrieve&dopt=full_report&list_uids=129831) |
| UL46 | SSNA1 | Sjogren syndrome nuclear autoantigen 1 | [8636](http://www.ncbi.nlm.nih.gov/entrez/query.fcgi?db=gene&cmd=Retrieve&dopt=full_report&list_uids=8636) |
| UL46 | TRAF2 | TNF receptor-associated factor 2 | [7186](http://www.ncbi.nlm.nih.gov/entrez/query.fcgi?db=gene&cmd=Retrieve&dopt=full_report&list_uids=7186) |
| UL47 | GKAP1 | G kinase anchoring protein 1 | [80318](http://www.ncbi.nlm.nih.gov/entrez/query.fcgi?db=gene&cmd=Retrieve&dopt=full_report&list_uids=80318) |
| UL49 | SH3KBP1 | SH3-domain kinase binding protein 1 | [30011](http://www.ncbi.nlm.nih.gov/entrez/query.fcgi?db=gene&cmd=Retrieve&dopt=full_report&list_uids=30011) |
| UL53 | IKZF3 | IKAROS family zinc finger 3 (Aiolos) | [22806](http://www.ncbi.nlm.nih.gov/entrez/query.fcgi?db=gene&cmd=Retrieve&dopt=full_report&list_uids=22806) |
| UL53 | TSPYL1 | TSPY-like 1 | [7259](http://www.ncbi.nlm.nih.gov/entrez/query.fcgi?db=gene&cmd=Retrieve&dopt=full_report&list_uids=7259) |
| UL56 | FAM64A | family with sequence similarity 64, member A | [54478](http://www.ncbi.nlm.nih.gov/entrez/query.fcgi?db=gene&cmd=Retrieve&dopt=full_report&list_uids=54478) |
| UL56 | IKZF3 | IKAROS family zinc finger 3 (Aiolos) | [22806](http://www.ncbi.nlm.nih.gov/entrez/query.fcgi?db=gene&cmd=Retrieve&dopt=full_report&list_uids=22806) |
| UL56 | ZC4H2 | zinc finger, C4H2 domain containing | [55906](http://www.ncbi.nlm.nih.gov/entrez/query.fcgi?db=gene&cmd=Retrieve&dopt=full_report&list_uids=55906) |
| UL7 | CTSD | cathepsin D | [1509](http://www.ncbi.nlm.nih.gov/entrez/query.fcgi?db=gene&cmd=Retrieve&dopt=full_report&list_uids=1509) |
| UL7 | KCNE3 | potassium voltage-gated channel, Isk-related family, member 3 | [10008](http://www.ncbi.nlm.nih.gov/entrez/query.fcgi?db=gene&cmd=Retrieve&dopt=full_report&list_uids=10008) |
| UL7 | MEIS2 | Meis homeobox 2 | [4212](http://www.ncbi.nlm.nih.gov/entrez/query.fcgi?db=gene&cmd=Retrieve&dopt=full_report&list_uids=4212) |
| UL7 | PIK3R2 | phosphoinositide-3-kinase, regulatory subunit 2 (p85 beta) | [5296](http://www.ncbi.nlm.nih.gov/entrez/query.fcgi?db=gene&cmd=Retrieve&dopt=full_report&list_uids=5296) |
| UL7 | PPP3CC | protein phosphatase 3 (formerly 2B), catalytic subunit, gamma isoform | [5533](http://www.ncbi.nlm.nih.gov/entrez/query.fcgi?db=gene&cmd=Retrieve&dopt=full_report&list_uids=5533) |
| US8 | ZC4H2 | zinc finger, C4H2 domain containing | [55906](http://www.ncbi.nlm.nih.gov/entrez/query.fcgi?db=gene&cmd=Retrieve&dopt=full_report&list_uids=55906) |
| US9 | ZC4H2 | zinc finger, C4H2 domain containing | [55906](http://www.ncbi.nlm.nih.gov/entrez/query.fcgi?db=gene&cmd=Retrieve&dopt=full_report&list_uids=55906) |
| UL10 | RALYL | RALY RNA binding protein-like | [138046](http://www.ncbi.nlm.nih.gov/entrez/query.fcgi?db=gene&cmd=Retrieve&dopt=full_report&list_uids=138046) |
| UL20 | ARL1 | ADP-ribosylation factor-like 1 | [400](http://www.ncbi.nlm.nih.gov/entrez/query.fcgi?db=gene&cmd=Retrieve&dopt=full_report&list_uids=400) |
| UL20 | GUK1 | guanylate kinase 1 | [2987](http://www.ncbi.nlm.nih.gov/entrez/query.fcgi?db=gene&cmd=Retrieve&dopt=full_report&list_uids=2987) |
| UL20 | PREPL | prolyl endopeptidase-like | [9581](http://www.ncbi.nlm.nih.gov/entrez/query.fcgi?db=gene&cmd=Retrieve&dopt=full_report&list_uids=9581) |
| UL20 | SRP9 | signal recognition particle 9kDa | [6726](http://www.ncbi.nlm.nih.gov/entrez/query.fcgi?db=gene&cmd=Retrieve&dopt=full_report&list_uids=6726) |
| UL22 | JTB | jumping translocation breakpoint | [10899](http://www.ncbi.nlm.nih.gov/entrez/query.fcgi?db=gene&cmd=Retrieve&dopt=full_report&list_uids=10899) |
| UL22 | NAPA | N-ethylmaleimide-sensitive factor attachment protein, alpha | [8775](http://www.ncbi.nlm.nih.gov/entrez/query.fcgi?db=gene&cmd=Retrieve&dopt=full_report&list_uids=8775) |
| UL22 | SERPINF1 | serpin peptidase inhibitor, clade F (alpha-2 antiplasmin, pigment epithelium derived factor), member 1 | [5176](http://www.ncbi.nlm.nih.gov/entrez/query.fcgi?db=gene&cmd=Retrieve&dopt=full_report&list_uids=5176) |
| UL22 | SPEG | SPEG complex locus | [10290](http://www.ncbi.nlm.nih.gov/entrez/query.fcgi?db=gene&cmd=Retrieve&dopt=full_report&list_uids=10290) |
| UL44 | NCOA5 | nuclear receptor coactivator 5 | [57727](http://www.ncbi.nlm.nih.gov/entrez/query.fcgi?db=gene&cmd=Retrieve&dopt=full_report&list_uids=57727) |
| UL46 | MKRN2 | makorin, ring finger protein, 2 | [23609](http://www.ncbi.nlm.nih.gov/entrez/query.fcgi?db=gene&cmd=Retrieve&dopt=full_report&list_uids=23609) |
| UL53 | GLYR1 | glyoxylate reductase 1 homolog (Arabidopsis) | [84656](http://www.ncbi.nlm.nih.gov/entrez/query.fcgi?db=gene&cmd=Retrieve&dopt=full_report&list_uids=84656) |
| UL7 | ACTA2 | actin, alpha 2, smooth muscle, aorta | [59](http://www.ncbi.nlm.nih.gov/entrez/query.fcgi?db=gene&cmd=Retrieve&dopt=full_report&list_uids=59) |
| UL7 | ELK1 | ELK1, member of ETS oncogene family | [2002](http://www.ncbi.nlm.nih.gov/entrez/query.fcgi?db=gene&cmd=Retrieve&dopt=full_report&list_uids=2002) |
| RL2 | PRDM14 | PR domain containing 14 | [63978](http://www.ncbi.nlm.nih.gov/entrez/query.fcgi?db=gene&cmd=Retrieve&dopt=full_report&list_uids=63978) |
| UL20 | S100A7 | S100 calcium binding protein A7 | [6278](http://www.ncbi.nlm.nih.gov/entrez/query.fcgi?db=gene&cmd=Retrieve&dopt=full_report&list_uids=6278) |
| UL26 | BOLL | bol, boule-like (Drosophila) | [66037](http://www.ncbi.nlm.nih.gov/entrez/query.fcgi?db=gene&cmd=Retrieve&dopt=full_report&list_uids=66037) |
| UL38 | GOLGA2 | golgi autoantigen, golgin subfamily a, 2 | [2801](http://www.ncbi.nlm.nih.gov/entrez/query.fcgi?db=gene&cmd=Retrieve&dopt=full_report&list_uids=2801) |
| UL38 | PRDM14 | PR domain containing 14 | [63978](http://www.ncbi.nlm.nih.gov/entrez/query.fcgi?db=gene&cmd=Retrieve&dopt=full_report&list_uids=63978) |
| UL40 | TCEAL8 | transcription elongation factor A (SII)-like 8 | [90843](http://www.ncbi.nlm.nih.gov/entrez/query.fcgi?db=gene&cmd=Retrieve&dopt=full_report&list_uids=90843) |
| UL49 | FRS3 | fibroblast growth factor receptor substrate 3 | [10817](http://www.ncbi.nlm.nih.gov/entrez/query.fcgi?db=gene&cmd=Retrieve&dopt=full_report&list_uids=10817) |
| UL56 | PCNP | PEST proteolytic signal containing nuclear protein | [57092](http://www.ncbi.nlm.nih.gov/entrez/query.fcgi?db=gene&cmd=Retrieve&dopt=full_report&list_uids=57092) |
| UL56 | RBCK1 | RanBP-type and C3HC4-type zinc finger containing 1 | [10616](http://www.ncbi.nlm.nih.gov/entrez/query.fcgi?db=gene&cmd=Retrieve&dopt=full_report&list_uids=10616) |
| UL7 | CRYAB | crystallin, alpha B | [1410](http://www.ncbi.nlm.nih.gov/entrez/query.fcgi?db=gene&cmd=Retrieve&dopt=full_report&list_uids=1410) |
| UL7 | HGS | hepatocyte growth factor-regulated tyrosine kinase substrate | [9146](http://www.ncbi.nlm.nih.gov/entrez/query.fcgi?db=gene&cmd=Retrieve&dopt=full_report&list_uids=9146) |
| US1 | MCRS1 | microspherule protein 1 | [10445](http://www.ncbi.nlm.nih.gov/entrez/query.fcgi?db=gene&cmd=Retrieve&dopt=full_report&list_uids=10445) |
| US10 | PRDM14 | PR domain containing 14 | [63978](http://www.ncbi.nlm.nih.gov/entrez/query.fcgi?db=gene&cmd=Retrieve&dopt=full_report&list_uids=63978) |
| US9 | MED25 | mediator of RNA polymerase II transcription, subunit 25 homolog (S. cerevisiae) | [81857](http://www.ncbi.nlm.nih.gov/entrez/query.fcgi?db=gene&cmd=Retrieve&dopt=full_report&list_uids=81857) |
| UL15 | CALCOCO2 | calcium binding and coiled-coil domain 2 | [10241](http://www.ncbi.nlm.nih.gov/entrez/query.fcgi?db=gene&cmd=Retrieve&dopt=full_report&list_uids=10241) |
| UL22 | NDUFV3 | NADH dehydrogenase (ubiquinone) flavoprotein 3, 10kDa | [4731](http://www.ncbi.nlm.nih.gov/entrez/query.fcgi?db=gene&cmd=Retrieve&dopt=full_report&list_uids=4731) |
| UL22 | RPS12 | ribosomal protein S12 | [6206](http://www.ncbi.nlm.nih.gov/entrez/query.fcgi?db=gene&cmd=Retrieve&dopt=full_report&list_uids=6206) |
| UL28 | PNMA1 | paraneoplastic antigen MA1 | [9240](http://www.ncbi.nlm.nih.gov/entrez/query.fcgi?db=gene&cmd=Retrieve&dopt=full_report&list_uids=9240) |
| UL34 | CCNDBP1 | cyclin D-type binding-protein 1 | [23582](http://www.ncbi.nlm.nih.gov/entrez/query.fcgi?db=gene&cmd=Retrieve&dopt=full_report&list_uids=23582) |
| UL35 | DYNLT3 | dynein, light chain, Tctex-type 3 | [6990](http://www.ncbi.nlm.nih.gov/entrez/query.fcgi?db=gene&cmd=Retrieve&dopt=full_report&list_uids=6990) |
| UL36 | CCNDBP1 | cyclin D-type binding-protein 1 | [23582](http://www.ncbi.nlm.nih.gov/entrez/query.fcgi?db=gene&cmd=Retrieve&dopt=full_report&list_uids=23582) |
| UL44 | KRTAP4-12 | keratin associated protein 4-12 | [83755](http://www.ncbi.nlm.nih.gov/entrez/query.fcgi?db=gene&cmd=Retrieve&dopt=full_report&list_uids=83755) |
| US8 | DNAJA3 | DnaJ (Hsp40) homolog, subfamily A, member 3 | [9093](http://www.ncbi.nlm.nih.gov/entrez/query.fcgi?db=gene&cmd=Retrieve&dopt=full_report&list_uids=9093) |
| US9 | RPS28 | ribosomal protein S28 | [6234](http://www.ncbi.nlm.nih.gov/entrez/query.fcgi?db=gene&cmd=Retrieve&dopt=full_report&list_uids=6234) |
| UL47 | SF3B4 | splicing factor 3b, subunit 4, 49kDa | [10262](http://www.ncbi.nlm.nih.gov/entrez/query.fcgi?db=gene&cmd=Retrieve&dopt=full_report&list_uids=10262) |
| UL53 | CCDC33 | coiled-coil domain containing 33 | [80125](http://www.ncbi.nlm.nih.gov/entrez/query.fcgi?db=gene&cmd=Retrieve&dopt=full_report&list_uids=80125) |
| UL17 | SF3B4 | splicing factor 3b, subunit 4, 49kDa | [10262](http://www.ncbi.nlm.nih.gov/entrez/query.fcgi?db=gene&cmd=Retrieve&dopt=full_report&list_uids=10262) |
| UL19 | SF3B4 | splicing factor 3b, subunit 4, 49kDa | [10262](http://www.ncbi.nlm.nih.gov/entrez/query.fcgi?db=gene&cmd=Retrieve&dopt=full_report&list_uids=10262) |

*^a^* HSV-1 gene cloned into pGBKT7-GATEWAY ‘Bait’ vector.

*^b^* Cellular gene cloned into pGADT7 ‘prey’ vector.

*^c^* Entrz Gene ID with hyperlink.

**Table S4:** Known interactors of HSV-1

| **Symbol** | **GeneId** | **Hit source(s)*^a^*** | **Virus*^b^*** | **Virus Gene** | **Virus GeneId** | **Direct?** | **References*^c^*** |
| --- | --- | --- | --- | --- | --- | --- | --- |
| BIRC5 | 332 | RNAi_5%; Y2H_ALL | EBV | LMP-1 | 3783750 | no | 16246267 |
| CSK | 1445 | Y2H_HC; RNAi_5% | EBV | LMP-2A | 3783751 | yes | 10233937 |
| LZTS2 | 84445 | Y2H_HC | EBV | BRRF1 | 3783728 | yes | 17446270 |
| LZTS2 | 84445 | Y2H_HC | EBV | BALF4 | 3783680 | yes | 17446270 |
| CTBP1 | 1487 | Y2H_HC | HHV4-2 | gp30 | 5176150 | yes | 11462050 |
| CTBP1 | 1487 | Y2H_HC | EBV | EBNA-3A | 3783762 | yes | 12372828 |
| CTBP1 | 1487 | Y2H_HC | EBV | EBNA-3B/C | 3783763 | yes | 11462050 |
| CDR2 | 1039 | Y2H_HC | VZV | ORF27 | 1487666 | yes | UNPUBLISHED_HAAS_LAB_VZV_Y2H |
| SH3KBP1 | 30011 | Y2H_HC | HSV-1 | RL2 | 2703389 | yes | 15824310 |
| WWP2 | 11060 | Y2H_HC | HSV-2 | UL56 | 1487345 | yes | 18353951 |
| WWP2 | 11060 | Y2H_HC | EBV | LMP-2A | 3783751 | yes | 10683340 |
| FATE1 | 89885 | Y2H_HC | VZV | ORF24 | 1487693 | yes | UNPUBLISHED_HAAS_LAB_VZV_Y2H |
| MDFI | 4188 | Y2H_HC | EBV | EBNA-1 | 3783774 | yes | 17446270 |
| PLSCR1 | 5359 | Y2H_HC | EBV | BFLF2 | 3783698 | yes | 17446270 |
| PLSCR1 | 5359 | Y2H_HC | EBV | BARF0 | 3783682 | yes | 15542637 |
| PLSCR1 | 5359 | Y2H_HC | VZV | ORF27 | 1487666 | yes | UNPUBLISHED_HAAS_LAB_VZV_Y2H |
| PLSCR1 | 5359 | Y2H_HC | EBV | BRRF1 | 3783728 | yes | 17446270 |
| RBCK1 | 10616 | Y2H_HC | EBV | BGLF2 | 3783768 | yes | 17446270 |
| MCRS1 | 10445 | Y2H_HC | EBV | BRLF1 | 3783727 | yes | 16460827 |
| MCRS1 | 10445 | Y2H_HC | HSV-1 | US1 | 2703435 | yes | 9765390 |
| PNMA1 | 9240 | Y2H_HC | KSHV | ORF8 | 4961501 | yes | UNPUBLISHED_HAAS_LAB_KSHV_Y2H |
| PNMA1 | 9240 | Y2H_HC | KSHV | vIRF-2 | 4961491 | yes | UNPUBLISHED_HAAS_LAB_KSHV_Y2H |
| CBR3 | 874 | Y2H_HC | KSHV | ORF29 | 4961443 | yes | UNPUBLISHED_HAAS_LAB_KSHV_Y2H |
| CBR3 | 874 | Y2H_HC | KSHV | ORF28 | 4961507 | yes | UNPUBLISHED_HAAS_LAB_KSHV_Y2H |
| CBR3 | 874 | Y2H_HC | KSHV | ORF30 | 4961433 | yes | UNPUBLISHED_HAAS_LAB_KSHV_Y2H |
| DYNLL1 | 8655 | Y2H_HC | EBV | EBNA-3A | 3783762 | yes | 17446270 |
| RAD51 | 5888 | Y2H_HC | HSV-1 | UL29 | 2703458 | no | 15078960 |
| NONO | 4841 | Y2H_HC | EBV | EBNA-LP | 3783746 | yes | 18457437 |
| IKBKG | 8517 | Y2H_HC | KSHV | HHV8GK18_gp80 | 4961494 | yes | 12890756 |
| IKBKG | 8517 | Y2H_HC | EBV | LMP-1 | 3783750 | no | 14691250; 14576817 |
| PRPF8 | 10594 | RNAi_5% | EBV | EBNA-LP | 3783746 | yes | 18457437 |
| RPS2 | 6187 | RNAi_5% | EBV | EBNA-LP | 3783746 | yes | 18457437 |
| RPS2 | 6187 | RNAi_5% | hCMV | UL44 | 3077460 | no | 20007282 |
| NUMA1 | 4926 | RNAi_5% | KSHV | HHV8GK18_gp81 | 4961527 | yes | 18417561 |
| MED6 | 10001 | RNAi_5% | HSV-1 | UL48 | 2703416 | no | 14657022; 14983011; 14576168 |
| HTRA1 | 5654 | RNAi_5% | VZV | ORF9A | 4711773 | yes | UNPUBLISHED_HAAS_LAB_VZV_Y2H |
| HTRA1 | 5654 | RNAi_5% | VZV | ORF1 | 1487664 | yes | UNPUBLISHED_HAAS_LAB_VZV_Y2H |
| POLR2A | 5430 | RNAi_5% | HSV-1 | UL48 | 2703416 | no | 8602364 |
| SFPQ | 6421 | RNAi_5% | EBV | EBNA-LP | 3783746 | yes | 18457437 |
| SFRS2 | 6427 | RNAi_5% | HSV-1 | UL54 | 2703426 | no | 7666511 |
| XPO1 | 7514 | RNAi_5% | KSHV | vIRF-3 | 4961493 | yes | 16214130 |
| XPO1 | 7514 | RNAi_5% | KSHV | ORF45 | 4961474 | yes | 19116250 |
| XPO1 | 7514 | RNAi_5% | EBV | LMP-1 | 3783750 | no | 12860972 |
| XPO1 | 7514 | RNAi_5% | HSV-1 | UL47 | 2703415 | yes | 18715912 |
| MED26 | 9441 | RNAi_5% | HSV-1 | UL48 | 2703416 | no | 14657022; 14983011; 14576168 |
| RPL5 | 6125 | RNAi_5% | hCMV | UL44 | 3077460 | no | 20007282 |
| CANX^†^ | 821 | RNAi_5% | HSV-1 | UL44 | 2703410 | yes | 8918549 |
| CANX^†^ | 821 | RNAi_5% | HSV-1 | UL27 | 2703455 | yes | 8918549 |
| CANX^†^ | 821 | RNAi_5% | HSV-1 | US5 | 2703406 | yes | 8918549 |
| CANX | 821 | RNAi_5% | KSHV | K2 | 4961449 | no | 19386721 |
| SP1 | 6667 | RNAi_5% | HSV-1 | RS1 | 2703391 | no | 2993923 |
| SP1 | 6667 | RNAi_5% | HSV-1 | UL23 | 2703374 | no | 2842233; 2072909; 1846184 |
| SP1 | 6667 | RNAi_5% | EBV | EBNA-3B/3C | 3783763 | yes | 16014933 |
| SP1 | 6667 | RNAi_5% | EBV | BMRF1 | 3783718 | yes | 10545120; 15613302 |
| SP1 | 6667 | RNAi_5% | VZV | ORF68 | 1487709 | no | 10989187 |
| SP1 | 6667 | RNAi_5% | VZV | ORF62 | 1487695 | yes | 12855699 |
| SP1 | 6667 | RNAi_5% | hCMV | UL122 | 3077563 | yes | 8035517 |
| SP1 | 6667 | RNAi_5% | VZV | ORF62 | 1487699 | yes | 12855699 |
| SP1 | 6667 | RNAi_5% | VZV | ORF67 | 1487689 | no | 12477854 |
| SP1 | 6667 | RNAi_5% | hCMV | UL123 | 3077513 | no | 1385862 |
| SP1 | 6667 | RNAi_5% | EBV | BALF5 | 3783681 | yes | 10545120 |
| SP1 | 6667 | RNAi_5% | KSHV | HHV8GK18_gp81 | 4961527 | yes | 15367601 |
| TRAF1 | 7185 | RNAi_5% | KSHV | K15 | 4961473 | yes | 10400794 |
| TRAF1 | 7185 | RNAi_5% | KSHV | HHV8GK18_gp80 | 4961494 | yes | 10523854 |
| TRAF1 | 7185 | RNAi_5% | EBV | LMP-1 | 3783750 | yes | 8943365; 9151858; 9580648; 9733827 |
| RAD50 | 10111 | RNAi_5% | EBV | EBNA-LP | 3783746 | yes | 18457437 |
| RAD50 | 10111 | RNAi_5% | HSV-1 | UL29 | 2703458 | no | 15140983 |
| MED8 | 112950 | RNAi_5% | KSHV | ORF38 | 4961435 | yes | UNPUBLISHED_HAAS_LAB_KSHV_Y2H |
| MED8^†^ | 112950 | RNAi_5% | HSV-1 | UL48 | 2703416 | yes | 14576168; 14657022; 14983011 |
| CEBPB | 1051 | RNAi_5% | KSHV | HHV8GK18_gp83 | 4961465 | no | 20004457 |
| PVRL1^†^ | 5818 | RNAi_5% | HSV-1 | US6 | 2703444 | yes | 12915568 |
| IDE | 3416 | RNAi_5% | VZV | ORF68 | 1487709 | yes | 17553876 |
| MED14 | 9282 | RNAi_5% | HSV-1 | UL48 | 2703416 | no | 14657022; 14983011; 14576168 |
| RPL10 | 6134 | RNAi_5% | EBV | EBNA-LP | 3783746 | yes | 18457437 |
| PDE6G | 5148 | RNAi_5% | EBV | BALF4 | 3783680 | yes | 17446270 |
| SFRS7 | 6432 | RNAi_5% | VZV | ORF4 | 1487672 | yes | 19924249 |
| MED7 | 9443 | RNAi_5% | HSV-1 | UL48 | 2703416 | no | 14657022; 14983011; 14576168 |
| LMNA | 4000 | RNAi_5% | hCMV | UL97 | 3077517 | yes | 19165338 |
| LMNA^†^ | 4000 | RNAi_5% | HSV-1 | UL34 | 2703355 | yes | 15140953 |
| LMNA | 4000 | RNAi_5% | HSV-1 | UL29 | 2703458 | no | 15140983 |
| LMNA^†^ | 4000 | RNAi_5% | HSV-1 | US3 | 2703401 | yes | 17428859 |
| LMNA^†^ | 4000 | RNAi_5% | HSV-1 | UL31 | 2703350 | yes | 15140953 |
| LMNA | 4000 | RNAi_5% | EBV | BGLF4 | 3783704 | yes | 18815303 |
| EIF3B | 8662 | RNAi_5% | EBV | EBNA-LP | 3783746 | yes | 18457437 |
| EIF3B^†^ | 8662 | RNAi_5% | HSV-1 | UL54 | 2703426 | yes | 15567442 |
| CSE1L | 1434 | RNAi_5% | EBV | EBNA-LP | 3783746 | yes | 18457437 |
| RPS16 | 6217 | RNAi_5% | EBV | EBNA-LP | 3783746 | yes | 18457437 |
| RPS9 | 6203 | RNAi_5% | EBV | EBNA-LP | 3783746 | yes | 18457437 |
| RPS9 | 6203 | RNAi_5% | hCMV | UL44 | 3077460 | no | 20007282 |
| TAF4 | 6874 | RNAi_5% | hCMV | UL123 | 3077513 | yes | 9311796 |
| TAF4 | 6874 | RNAi_5% | hCMV | UL122 | 3077563 | yes | 9311796 |
| SFRS3 | 6428 | RNAi_5% | VZV | ORF4 | 1487672 | yes | 19924249 |
| SFRS3 | 6428 | RNAi_5% | HSV-1 | UL54 | 2703426 | no | 12660167; 19553338 |
| RECK | 8434 | RNAi_5% | EBV | LMP-1 | 3783750 | no | 14614450 |
| HLA-G | 3135 | RNAi_5% | hCMV | US2 | 3077542 | yes | 14662880 |
| HLA-G | 3135 | RNAi_5% | hCMV | US3 | 3077532 | yes | 10623826 |
| PSMD2 | 5708 | RNAi_5% | EBV | EBNA-LP | 3783746 | yes | 18457437 |
| SMARCA5 | 8467 | RNAi_5% | HSV-1 | UL29 | 2703458 | no | 15140983 |
| PSMB6 | 5694 | RNAi_5% | EBV | EBNA-LP | 3783746 | yes | 18457437 |
| ECH1 | 1891 | RNAi_5% | KSHV | ORF62 | 4961461 | yes | UNPUBLISHED_HAAS_LAB_KSHV_Y2H |
| HIST2H2BE | 8349 | RNAi_5% | MHV1 | MuHV1_gpm123Ex2 | 3293881 | no | 2833016 |
| HIST2H2BE | 8349 | RNAi_5% | KSHV | HHV8GK18_gp81 | 4961527 | yes | 16469929 |
| SIAH1 | 6477 | RNAi_5% | EBV | LMP-1 | 3783750 | no | 16344472; 17047048 |
| RBL1 | 5933 | RNAi_5% | HSV-1 | RS1 | 2703392 | no | 10366563 |
| RBL1 | 5933 | RNAi_5% | HSV-1 | UL29 | 2703458 | no | 10366563 |
| RBL1 | 5933 | RNAi_5% | hCMV | UL123 | 3077513 | yes | 8892909; 10355776 |
| DAXX | 1616 | RNAi_5% | hCMV | UL82 | 3077530 | yes | 11992005; 18922870; 15919932 |
| DAXX | 1616 | RNAi_5% | hCMV | UL123 | 3077513 | yes | 12502852 |
| MED24 | 9862 | RNAi_5% | HSV-1 | UL48 | 2703416 | no | 14657022; 14983011; 14576168 |
| BAD | 572 | RNAi_5% | HSV-1 | US3 | 2703401 | no | 11517326 |
| RUNX3 | 864 | RNAi_5% | EBV | EBNA-2 | 3783761 | no | 11967309 |
| CDKN2A | 1029 | RNAi_5% | EBV | LMP-1 | 3783750 | no | 10803461 |
| CDKN2A | 1029 | RNAi_5% | EBV | EBNA-LP | 3783746 | yes | 12740913 |
| CR2 | 1380 | RNAi_5% | KSHV | ORF50 | 4961526 | no | 15795251 |
| CR2 | 1380 | RNAi_5% | EBV | LMP-1 | 3783750 | no | 8093369; 1354347 |
| CR2 | 1380 | RNAi_5% | EBV | EBNA-2 | 3783761 | no | 8093369; 2153641; 2154588 |
| CR2 | 1380 | RNAi_5% | EBV | BLLF1 | 3783713 | yes | 3036369; 3033269; 2464439; 2555366; 18786993 |
| H1F0 | 3005 | RNAi_5% | KSHV | HHV8GK18_gp81 | 4961527 | no | 10562490 |
| RAC1 | 5879 | RNAi_5% | HSV-2 | US3 | 1487355 | no | 11168588 |
| RAC1 | 5879 | RNAi_5% | KSHV | HHV8GK18_gp83 | 4961465 | no | 15231571 |
| KIF2A | 3796 | RNAi_5% | EBV | EBNA-3B/EBNA-3C | 3783763 | no | 16014933 |
| DYNC1I1^†^ | 1780 | RNAi_5% | HSV-1 | UL34 | 2703355 | yes | 10627546 |
| MYO5A | 4644 | RNAi_5% | VZV | ORF34 | 1487687 | yes | UNPUBLISHED_HAAS_LAB_VZV_Y2H |
| MLF2 | 8079 | RNAi_5% | EBV | EBNA-LP | 3783746 | yes | 18457437 |
| STAT3 | 6774 | RNAi_5% | KSHV | ORF50 | 4961526 | yes | 11741976 |
| STAT3 | 6774 | RNAi_5% | EBV | LMP-1 | 3783750 | no | 11222718 |
| STAT3 | 6774 | RNAi_5% | EBV | LMP-2A | 3783751 | no | 19339266 |
| STAT3 | 6774 | RNAi_5% | KSHV | K2 | 4961449 | no | 10566591 |
| STAT3 | 6774 | RNAi_5% | EBV | EBNA-1 | 3783709 | no | 11222718 |
| STAT3 | 6774 | RNAi_5% | EBV | EBNA-1 | 3783774 | no | 11222718 |
| STAT3 | 6774 | RNAi_5% | EBV | EBNA-2 | 3783761 | no | 19048596 |
| STAT3 | 6774 | RNAi_5% | KSHV | HHV8GK18_gp81 | 4961527 | yes | 16364321 |
| HSP90AA1 | 3320 | RNAi_5% | EBV | EBNA-LP | 3783746 | yes | 18457437 |
| TADA2A^†^ | 6871 | RNAi_5% | HSV-1 | UL48 | 2703416 | yes | 8189518; 7972120 |
| FCER2 | 2208 | RNAi_5% | EBV | EBNA-3A | 3783762 | no | 3033649; PMID:1649318 |
| FCER2 | 2208 | RNAi_5% | EBV | EBNA-2 | 3783761 | no | 8093369; 2153641; 2154588 |
| FCER2 | 2208 | RNAi_5% | EBV | LMP-1 | 3783750 | no | 2352328; 1354347 |
| FCER2 | 2208 | RNAi_5% | KSHV | ORF50 | 4961526 | no | 15795251 |
| CBX5 | 23468 | RNAi_5% | KSHV | HHV8GK18_gp81 | 4961527 | yes | 12486118 |
| CBX5 | 23468 | RNAi_5% | KSHV | ORF19 | 4961508 | yes | UNPUBLISHED_HAAS_LAB_KSHV_Y2H |
| GTF3C1 | 2975 | RNAi_5% | KSHV | ORF29 | 4961443 | yes | UNPUBLISHED_HAAS_LAB_KSHV_Y2H |
| GTF3C1 | 2975 | RNAi_5% | KSHV | ORF16 | 4961447 | yes | UNPUBLISHED_HAAS_LAB_KSHV_Y2H |
| GTF3C1 | 2975 | RNAi_5% | KSHV | ORF30 | 4961433 | yes | UNPUBLISHED_HAAS_LAB_KSHV_Y2H |
| GTF3C1 | 2975 | RNAi_5% | HSV-1 | UL54 | 2703426 | no | 1320373 |
| GTF3C1 | 2975 | RNAi_5% | KSHV | ORF28 | 4961507 | yes | UNPUBLISHED_HAAS_LAB_KSHV_Y2H |
| ICAM5^†^ | 7087 | RNAi_5% | HSV-1 | RL1 | 2703395 | yes | 19589604 |
| ICAM5^†^ | 7087 | RNAi_5% | HSV-1 | RL1 | 2703396 | yes | 19589604 |
| MED23 | 9439 | RNAi_5% | HSV-1 | UL48 | 2703416 | no | 14657022; 14983011; 14576168 |
| NME1 | 4830 | Y2H_ALL | EBV | EBNA-3B/EBNA-3C | 3783763 | yes | 12163590 |
| NME1 | 4830 | Y2H_ALL | EBV | EBNA-LP | 3783746 | yes | 15650182 |
| DNAJA3 | 9093 | Y2H_ALL | HSV-1 | UL9 | 2703434 | yes | 11854491 |
| DNAJA3 | 9093 | Y2H_ALL | EBV | BARF1 | 3783772 | yes | 16518412 |
| DNAJA3 | 9093 | Y2H_ALL | VZV | ORF42 | 1487719 | yes | UNPUBLISHED_HAAS_LAB_VZV_Y2H |
| CCL19 | 6363 | Y2H_ALL | EBV | LMP-1 | 3783750 | no | 14691250 |
| STX11 | 8676 | Y2H_ALL | VZV | ORF27 | 1487666 | yes | UNPUBLISHED_HAAS_LAB_VZV_Y2H |
| RPS17 | 6218 | Y2H_ALL | EBV | EBNA-LP | 3783746 | yes | 18457437 |
| PLEK | 5341 | Y2H_ALL | EBV | EBNA-3A | 3783762 | no | 8862411 |
| TRAF6 | 7189 | Y2H_ALL | EBV | LMP-1 | 3783750 | no | 16446357; 11598011; 14673102; 16477006; 16280329; 14673155 |
| PIK3R2 | 5296 | Y2H_ALL | KSHV | HHV8GK18_gp83 | 4961465 | no | 11799169 |
| PIK3R2 | 5296 | Y2H_ALL | hCMV | UL122 | 3077563 | no | 11907212 |
| PIK3R2 | 5296 | Y2H_ALL | EBV | LMP-2A | 3783751 | no | 11044112 |
| PIK3R2 | 5296 | Y2H_ALL | hCMV | UL123 | 3077513 | no | 11907212 |
| PIK3R2 | 5296 | Y2H_ALL | EBV | LMP-1 | 3783750 | no | 12446712 |
| ING5 | 84289 | Y2H_ALL | KSHV | ORF65 | 4961451 | yes | UNPUBLISHED_HAAS_LAB_KSHV_Y2H |
| ING5 | 84289 | Y2H_ALL | KSHV | ORF62 | 4961461 | yes | UNPUBLISHED_HAAS_LAB_KSHV_Y2H |
| ING5 | 84289 | Y2H_ALL | KSHV | HHV8GK18_gp77 | 4961470 | yes | UNPUBLISHED_HAAS_LAB_KSHV_Y2H |
| ING5 | 84289 | Y2H_ALL | KSHV | ORF55 | 4961432 | yes | UNPUBLISHED_HAAS_LAB_KSHV_Y2H |
| ING5 | 84289 | Y2H_ALL | KSHV | ORF19 | 4961508 | yes | UNPUBLISHED_HAAS_LAB_KSHV_Y2H |
| TSPO | 706 | Y2H_ALL | VZV | ORF15 | 1487652 | yes | UNPUBLISHED_HAAS_LAB_VZV_Y2H |
| DYNLT3 | 6990 | Y2H_ALL | HSV-1 | UL35 | 2703356 | yes | 15117959 |
| MED25 | 81857 | Y2H_ALL | VZV | ORF62 | 1487695 | yes | 18842726 |
| MED25 | 81857 | Y2H_ALL | VZV | ORF62 | 1487699 | yes | 18842726 |
| MED25 | 81857 | Y2H_ALL | HSV-1 | UL48 | 2703416 | yes | 14657022; 14983011; 14576168 |
| GUK1 | 2987 | Y2H_ALL | VZV | ORF9A | 4711773 | yes | UNPUBLISHED_HAAS_LAB_VZV_Y2H |
| CPSF4 | 10898 | Y2H_ALL | HSV-1 | UL48 | 2703416 | no | 17135252 |
| TRAF2 | 7186 | Y2H_ALL | EBV | BRRF1 | 3783728 | yes | 17446270 |
| TRAF2 | 7186 | Y2H_ALL | KSHV | K15 | 4961473 | yes | 12915550; 10400794 |
| TRAF2 | 7186 | Y2H_ALL | EBV | BALF4 | 3783680 | yes | 17446270 |
| TRAF2 | 7186 | Y2H_ALL | KSHV | HHV8GK18_gp80 | 4961494 | yes | 10523854; 16311516 |
| TRAF2 | 7186 | Y2H_ALL | EBV | LMP-1 | 3783750 | yes | 8943365; 9151858 |
| FOS | 2353 | Y2H_ALL | KSHV | ORF50 | 4961526 | no | 15047839 |
| FOS | 2353 | Y2H_ALL | KSHV | K8 | 4961462 | no | 15047839 |
| FOS | 2353 | Y2H_ALL | hCMV | UL123 | 3077513 | no | 1328493; 1325808 |
| FOS | 2353 | Y2H_ALL | hCMV | UL122 | 3077563 | no | 1328493; 1325808 |
| MYST2 | 11143 | Y2H_ALL | KSHV | ORF19 | 4961508 | yes | UNPUBLISHED_HAAS_LAB_KSHV_Y2H |
| UNC119 | 9094 | Y2H_ALL | KSHV | ORF55 | 4961432 | yes | UNPUBLISHED_HAAS_LAB_KSHV_Y2H |
| UNC119 | 9094 | Y2H_ALL | KSHV | ORF62 | 4961461 | yes | UNPUBLISHED_HAAS_LAB_KSHV_Y2H |
| TSC22D4 | 81628 | Y2H_ALL | EBV | BGRF1/BDRF1 | 3783767 | yes | 17446270 |
| IRF1 | 3659 | Y2H_ALL | hCMV | UL83 | 3077579 | no | 12972646 |
| IRF1 | 3659 | Y2H_ALL | EBV | EBNA-1 | 3783774 | no | 9261415 |
| IRF1 | 3659 | Y2H_ALL | KSHV | vIRF-2 | 4961491 | yes | 10200596; 16501120 |
| IRF1 | 3659 | Y2H_ALL | KSHV | vIRF-1 | 4961464 | yes | 10438822 |
| PTPN6 | 5777 | RNAi_10% | KSHV | K1 | 4961511 | yes | 16160144 |
| MED21 | 9412 | RNAi_10% | HSV-1 | UL48 | 2703416 | no | 8871557; 14657022; 14983011; 14576168 |
| GTF2A1 | 2957 | RNAi_10% | EBV | BZLF1 | 3783744 | yes | 7565798 |
| GTF2A1 | 2957 | RNAi_10% | HSV-1 | UL48 | 2703416 | yes | 7565798 |
| HCFC1 | 3054 | RNAi_10% | VZV | ORF10 | 1487675 | yes | 7609034 |
| HCFC1 | 3054 | RNAi_10% | HSV-1 | UL48 | 2703416 | yes | 9094665; 2571937; 1980658 |
| RPL13A | 23521 | RNAi_10% | hCMV | UL44 | 3077460 | no | 20007282 |
| RPL13A | 23521 | RNAi_10% | EBV | LMP-1 | 3783750 | no | 11513954 |
| RPL11 | 6135 | RNAi_10% | EBV | EBNA-LP | 3783746 | yes | 18457437 |
| PVRL2 | 5819 | RNAi_10% | HSV-1 | US6 | 2703444 | yes | 12915568 |
| MED27 | 9442 | RNAi_10% | HSV-1 | UL48 | 2703416 | no | 14657022; 14983011; 14576168 |
| USF2 | 7392 | RNAi_10% | EBV | LMP-1 | 3783750 | no | 10074148 |
| HNRNPK | 3190 | RNAi_10% | KSHV | ORF57 | 4961525 | yes | 15486205 |
| HNRNPK | 3190 | RNAi_10% | HHV 6A | U86 | 1487966 | yes | 15031534 |
| HNRNPK | 3190 | RNAi_10% | HSV-1 | UL54 | 2703426 | yes | 10506147 |
| HNRNPK | 3190 | RNAi_10% | EBV | EBNA-LP | 3783746 | yes | 18457437 |
| FGR | 2268 | RNAi_10% | EBV | EBNA-3A | 3783762 | no | 2159528 |
| ANXA2 | 302 | RNAi_10% | hCMV | UL55 | 3077424 | yes | 9371650 |
| ANXA2 | 302 | RNAi_10% | EBV | LMP-1 | 3783750 | no | 11513954; 16989986 |
| CREBZF | 58487 | RNAi_10% | HSV-1 | UL48 | 2703416 | no | 16282471 |
| TFAM | 7019 | RNAi_10% | EBV | BZLF1 | 3783744 | no | 19656881 |
| LRPPRC | 10128 | RNAi_10% | EBV | EBNA-LP | 3783746 | yes | 18457437 |
| TUBA1B | 10376 | RNAi_10% | EBV | EBNA-LP | 3783746 | yes | 18457437 |
| ZNF148 | 7707 | RNAi_10% | EBV | BALF5 | 3783681 | yes | 10545120 |
| ZNF148 | 7707 | RNAi_10% | EBV | BMRF1 | 3783718 | yes | 10545120; 15613302 |
| NFKB2 | 4791 | RNAi_10% | EBV | LMP-1 | 3783750 | no | 14576817 |
| OPTN | 10133 | RNAi_10% | EBV | BGLF2 | 3783768 | yes | 17446270 |
| RPS19 | 6223 | RNAi_10% | hCMV | UL44 | 3077460 | no | 20007282 |
| LDOC1 | 23641 | RNAi_10% | VZV | ORF33.5 | 4711772 | yes | UNPUBLISHED_HAAS_LAB_VZV_Y2H |
| TERT | 7015 | RNAi_10% | EBV | LMP-2A | 3783751 | no | 15389515 |
| TERT | 7015 | RNAi_10% | EBV | LMP-1 | 3783750 | no | 15967702 |
| NEDD4 | 4734 | RNAi_10% | EBV | LMP-2A | 3783751 | yes | 10683340 |
| NEDD4 | 4734 | RNAi_10% | HSV-2 | UL56 | 1487345 | yes | 18353951 |
| TMEM98 | 26022 | RNAi_10% | KSHV | ORF28 | 4961507 | yes | UNPUBLISHED_HAAS_LAB_KSHV_Y2H |
| KCNAB2 | 8514 | RNAi_10% | VZV | ORF68 | 1487709 | yes | UNPUBLISHED_HAAS_LAB_VZV_Y2H |
| KHDRBS1 | 10657 | RNAi_10% | EBV | EBNA-LP | 3783746 | yes | 18457437 |
| CASP8 | 841 | RNAi_10% | KSHV | K8 | 4961462 | yes | UNPUBLISHED_HAAS_LAB_KSHV_Y2H |
| CASP8 | 841 | RNAi_10% | KSHV | ORF55 | 4961432 | yes | UNPUBLISHED_HAAS_LAB_KSHV_Y2H |
| CASP8 | 841 | RNAi_10% | KSHV | ORF62 | 4961461 | yes | UNPUBLISHED_HAAS_LAB_KSHV_Y2H |
| KPNA1 | 3836 | RNAi_10% | EBV | EBNA-1 | 3783774 | yes | 16439554; 10612665 |
| KPNA1 | 3836 | RNAi_10% | hCMV | UL56 | 3077457 | yes | 10950981 |
| KPNA1 | 3836 | RNAi_10% | VZV | ORF43 | 1487670 | yes | UNPUBLISHED_HAAS_LAB_VZV_Y2H |
| CCR4 | 1233 | RNAi_10% | KSHV | K4.1 | 4961436 | yes | 10666184 |
| CDK8 | 1024 | RNAi_10% | HSV-1 | UL48 | 2703416 | no | 8871557 |
| HDLBP | 3069 | RNAi_10% | VZV | ORF48 | 1487679 | yes | UNPUBLISHED_HAAS_LAB_VZV_Y2H |
| SMARCC1 | 6599 | RNAi_10% | HSV-1 | UL29 | 2703458 | no | 15140983 |

*^a^* Source screen of HSV-1 hit. RNAi_10%, hit within the top 10% enhancing/inhibiting cut-off; RNAi_5%, hit within the top 10% enhancing/inhibiting cut-off; Y2H_ALL, within full HSV-1:host Y2H screen; Y2H_HC, high confidence hit from HSV-1:host Y2H screen.

*^b^* HHV4-2, human herpesvirus four type 2; MHV-1, murid herpesvirus type 1; HHV6A, human herpesvirus 6A.

*^c^* Reference for interaction, Pubmed ID or unpublished observations.

^†^ HDF identified in our RNAi screen that has a previously published interaction with HSV-1 viral protein(s)

**Table S5:** Overlap between HSV-1 siRNA and Y2H screens

| **Symbol** | **Name** | **Y2H detected interactors** |
| --- | --- | --- |
| SAA1 | serum amyloid A1 | UL22 (y2h_hc) |
| HMGN1 | high-mobility group nucleosome binding domain 1 | UL20 (y2h_all) |
| CSRNP1 | cysteine-serine-rich nuclear protein 1 | UL46 (y2h_all), UL47 (y2h_hc) |
| HNRNPUL1 | heterogeneous nuclear ribonucleoprotein U-like 1 | UL7 (y2h_all) |
| BIRC5 | baculoviral IAP repeat-containing 5 | UL22 (y2h_all) |
| HGF | hepatocyte growth factor (hepapoietin A; scatter factor) | UL20 (y2h_all) |
| RAB20 | RAB20, member RAS oncogene family | UL46 (y2h_all) |
| CSK | c-src tyrosine kinase | UL10 (y2h_hc) |
| OLAH | oleoyl-ACP hydrolase | UL22 (y2h_all) |
| HOXA10 | homeobox A10 | UL20 (y2h_all) |

**Table S6:** Validation of primary screen phenotypes by siRNA SMARTpool deconvolution and quantitative RT-PCR.

| **GENE SYMBOL** | **Individual siRNA** | | | | **Reconstituted SMARTpool** | **Primary Screen** |
| --- | --- | --- | --- | --- | --- | --- |
|  | **1** | **2** | **3** | **4** |  |  |
| GTF2A1 | 0.18 | 0.12 | 0.16 | 0.11 | 0.08 | 0.03 |
| GTF3C1*^a^* | 0.44 | 0.36 | 0.93 | 0.67 | 0.76 | 1.53 |
| GTF3C2 | 0.23 | 0.28 | 0.19 | 0.87 | 0.32 | 0.08 |
| MED4 | 0.19 | 0.07 | 0.16 | 0.08 | 0.09 | 0.17 |
| MED6 | 0.04 | 0.02 | 0.03 | 0.02 | 0.02 | 0.00 |
| MED7 | 0.24 | 0.05 | 0.03 | 0.09 | 0.03 | 0.03 |
| MED8 | 0.18 | 0.09 | 0.03 | 0.63 | 0.15 | 0.02 |
| MED14 | 0.08 | 0.05 | 0.04 | 0.05 | 0.06 | 0.02 |
| MED23 | 1.45 | 1.32 | 1.73 | 0.92 | 1.55 | 1.68 |
| TOP1 | 0.25 | 0.31 | 0.73 | 0.05 | 0.04 | 0.09 |
| TOP2A | 0.15 | 0.50 | 0.18 | 0.39 | 0.11 | 0.07 |
| TOP3B*^a^* | 0.60 | 0.66 | 0.38 | 0.78 | 0.95 | 1.58 |
| TOPBP1 | 0.19 | 0.13 | 0.08 | 0.14 | 0.05 | 0.04 |
| HOXA10*^a^* | 0.37 | 0.24 | 1.00 | 0.25 | 0.35 | 1.53 |
| HOXA5 | 0.67 | 0.41 | 0.07 | 0.44 | 0.29 | 0.18 |
| HOXC5 | 0.25 | 0.07 | 0.83 | 0.07 | 0.08 | 0.07 |
| CBX4 | 0.18 | 0.09 | 0.33 | 0.51 | 0.54 | 0.08 |
| CBX5*^a^* | 0.71 | 0.89 | 0.47 | 0.15 | 0.46 | 1.52 |
| NR1H2 | 0.35 | 0.04 | 0.71 | 0.41 | 0.36 | 0.05 |
| NR3C2*^a^* | 0.75 | 0.67 | 0.47 | 0.46 | 1.15 | 1.47 |
| NR4A1*^a^* | 0.18 | 0.02 | 0.39 | 0.11 | 0.06 | 0.75 |
| NR4A2 | 0.29 | 0.12 | 0.14 | 0.17 | 0.11 | 0.04 |
| IFITM1 | 0.12 | 0.08 | 0.42 | 0.08 | 0.07 | 0.15 |
| IFITM2 | 0.03 | 0.14 | 0.20 | 0.06 | 0.11 | 0.24 |
| IFITM3*^a^* | 0.31 | 0.07 | 0.11 | 0.34 | 0.15 | 0.86 |
| ITGA2 | 0.44 | 0.61 | 0.12 | 0.72 | 0.50 | 0.24 |
| ITGA6 | 0.05 | 0.41 | 0.38 | 0.93 | 0.35 | 0.24 |
| ITGAV | 1.18 | 0.14 | 0.48 | 0.14 | 0.51 | 0.20 |
| ITGB2 | 0.55 | 0.70 | 0.42 | 0.42 | 0.61 | 0.23 |
| ITGB3 | 0.28 | 0.50 | 0.59 | 0.10 | 0.22 | 0.07 |
| PVRL1 | 0.09 | 0.35 | 0.05 | 0.06 | 0.04 | 0.02 |
| PVRL2 | 0.09 | 0.23 | 0.19 | 0.24 | 0.17 | 0.04 |
| SCAMP1 | 0.31 | 0.67 | 0.43 | 0.11 | 0.29 | 0.14 |
| SCAMP3*^a^* | 0.76 | 0.20 | 0.54 | 0.88 | 0.38 | 0.26 |
| RAB10 | 0.10 | 0.57 | 0.16 | 0.39 | 0.09 | 0.05 |
| RAB13 | 0.10 | 0.28 | 0.49 | 0.32 | 0.05 | 0.04 |
| RAB26 | 0.32 | 0.10 | 0.11 | 0.69 | 0.44 | 0.15 |
| RAB6A | 0.15 | 0.15 | 0.39 | 0.19 | 0.09 | 0.04 |
| RAB6B | 0.63 | 0.11 | 0.38 | 0.85 | 0.48 | 0.22 |
| RALA | 1.04 | 1.04 | 0.04 | 0.08 | 0.18 | 0.22 |
| RALB | 0.09 | 0.51 | 0.16 | 0.15 | 0.12 | 0.15 |
| RALGPS2 | 0.23 | 0.07 | 0.22 | 0.03 | 0.04 | 0.10 |
| VAMP8*^a^* | 0.59 | 0.55 | 0.20 | 0.67 | 0.45 | 0.11 |
| EPIM | 0.41 | 0.60 | 0.48 | 0.26 | 0.50 | 0.25 |
| PNUTL1 | 0.26 | 0.18 | 0.05 | 0.56 | 0.10 | 0.24 |
| SMURF1 | 0.24 | 0.06 | 0.13 | 0.47 | 0.15 | 0.11 |
| SMURF2 | 0.06 | 0.04 | 0.10 | 0.67 | 0.03 | 0.07 |
| MYRIP | 0.29 | 0.04 | 0.92 | 0.44 | 0.20 | 0.30 |
| DNCI2 | 0.18 | 0.20 | 0.08 | 0.31 | 0.19 | 0.02 |
| DNCLI2 | 0.35 | 0.24 | 0.05 | 0.43 | 0.08 | 0.09 |
| DYNC1H1 | 0.04 | 0.15 | 0.09 | 0.23 | 0.12 | 0.03 |
| UBE2D1 | 0.44 | 0.32 | 0.56 | 0.24 | 0.53 | 0.49 |
| UBE2D2*^a^* | 0.89 | 0.18 | 0.21 | 0.54 | 0.45 | 0.59 |
| UBE2D3 | 0.58 | 0.14 | 0.19 | 0.39 | 0.29 | 0.18 |
| UBE2D4 | 0.11 | 0.50 | 0.62 | 0.27 | 0.23 | 0.07 |
| UBE2E1 | 0.16 | 0.13 | 0.83 | 0.59 | 0.08 | 0.20 |
| UBE2E2 | 1.06 | 0.61 | 0.76 | 0.47 | 0.80 | 0.96 |
| UBE2E3*^a^* | 0.61 | 0.51 | 0.68 | 0.27 | 0.38 | 0.47 |
| UBE2I | 0.40 | 0.41 | 0.48 | 0.17 | 0.46 | 0.23 |
| UBE2L3 | 0.32 | 0.27 | 0.53 | 0.33 | 0.25 | 0.34 |
| UBE2N | 0.35 | 0.18 | 0.59 | 0.21 | 0.14 | 0.28 |
| UBE2V1 | 0.80 | 0.70 | 0.26 | 0.43 | 0.68 | 0.86 |
| PSMB6 | 0.10 | 0.03 | 0.19 | 0.08 | 0.05 | 0.03 |

| PSMD4 | 0.17 | 0.13 | 0.14 | 0.13 | 0.06 | 0.05 |
| --- | --- | --- | --- | --- | --- | --- |
| PSMD5*^a^* | 0.72 | 0.54 | 1.02 | 0.73 | 1.03 | 1.56 |
| ANXA1 | 0.62 | 0.13 | 0.32 | 0.05 | 0.20 | 0.22 |
| MRC2 | 0.23 | 0.22 | 0.75 | 0.15 | 0.25 | 0.09 |
| N4BP2 | 0.41 | 0.17 | 0.47 | 0.40 | 0.24 | 0.23 |
| PLSCR1 | 0.06 | 0.11 | 0.29 | 0.08 | 0.28 | 0.32 |
| PLSCR3 | 0.22 | 0.85 | 0.12 | 0.23 | 0.23 | 0.50 |
| WRB | 0.52 | 0.80 | 0.05 | 0.16 | 0.16 | 0.69 |
| XPO1 | 0.02 | 0.07 | 0.04 | 0.05 | 0.01 | 0.01 |

*^a^* Validation candidate genes where <2 of 4 deconvoluted siRNAs reproduced the HSV-1 phenotype observed in the primary screen.

**Table S7:** Specificity of identified HFs to HSV-1 replication

| **GENE SYMBOL** | **α-herpesvirus** | | **β-herpesvirus** | **RNA Virus** |
| --- | --- | --- | --- | --- |
|  | **HSV** | **VZV** | **hCMV** | **SFV** |
| Mock | 1.00 | 0.96 | 1.00 | 1.00 |
| negative control | 0.93 | 1.00 | 0.77 | 0.92 |
| Positive control | 0.04 | 0.53 | 0.10 | 0.03 |
| GTF2A1 | 0.05 | 0.92 | 0.05 | 0.59 |
| GTF3C1 | 0.96 | 0.50 | 0.82 | 0.65 |
| GTF3C2 | 0.25 | 0.99 | 0.41 | 0.69 |
| MED4 | 0.23 | 0.96 | 0.34 | 0.52 |
| MED6 | 0.03 | 0.67 | 0.05 | 0.49 |
| MED7 | 0.04 | 1.22 | 0.12 | 0.25 |
| MED8 | 0.04 | 1.29 | 0.50 | 0.27 |
| MED14 | 0.14 | 0.43 | 0.14 | 0.69 |
| MED23 | 1.89 | 1.05 | 0.68 | 0.65 |
| TOP1 | 0.10 | 0.39 | 0.43 | 1.10 |
| TOP2A | 0.09 | 1.10 | 0.82 | 0.48 |
| TOP3B | 1.14 | 1.05 | 0.73 | 0.29 |
| TOPBP1 | 0.08 | 0.63 | 0.18 | 0.36 |
| HOXA10 | 0.28 | 0.89 | 0.42 | 0.91 |
| HOXA5 | 0.26 | 1.10 | 0.18 | 0.62 |
| HOXC5 | 0.03 | 0.56 | 0.19 | 0.13 |
| CBX4 | 0.15 | 0.84 | 0.59 | 0.23 |
| CBX5 | 0.32 | 1.03 | 0.86 | 0.70 |
| NR1H2 | 0.14 | 0.96 | 0.16 | 0.19 |
| NR3C2 | 1.35 | 1.02 | 0.70 | 0.36 |
| NR4A1 | 0.09 | 0.73 | 0.26 | 1.03 |
| NR4A2 | 0.10 | 0.30 | 0.30 | 0.45 |
| IFITM1 | 0.20 | 0.82 | 0.69 | 0.99 |
| IFITM2 | 0.13 | 1.26 | 0.72 | 0.36 |
| IFITM3 | 0.31 | 0.86 | 0.47 | 0.40 |
| ITGA2 | 0.17 | 0.91 | 0.61 | 0.71 |
| ITGA6 | 0.33 | 0.82 | 1.00 | 1.23 |
| ITGAV | 0.37 | 0.30 | 0.45 | 0.79 |
| ITGB2 | 0.69 | 1.03 | 0.42 | 0.63 |
| ITGB3 | 0.16 | 0.81 | 0.35 | 0.33 |
| PVRL1 | 0.05 | 0.91 | 0.33 | 1.21 |
| PVRL2 | 0.13 | 1.05 | 0.64 | 0.76 |
| SCAMP1 | 0.35 | 1.13 | 0.63 | 0.37 |
| SCAMP3 | 0.33 | 0.87 | 0.86 | 0.37 |
| RAB10 | 0.06 | 1.18 | 0.46 | 0.66 |
| RAB13 | 0.05 | 1.24 | 0.37 | 0.63 |
| RAB26 | 0.14 | 1.25 | 0.20 | 0.22 |
| RAB6A | 0.07 | 0.61 | 0.62 | 0.63 |
| RAB6B | 0.48 | 1.01 | 0.50 | 0.38 |
| RALA | 0.31 | 0.95 | 0.44 | 0.84 |
| RALB | 0.32 | 0.77 | 0.35 | 0.72 |
| RALGPS2 | 0.17 | 1.23 | 0.39 | 0.51 |
| VAMP8 | 0.60 | 1.04 | 0.29 | 0.49 |
| EPIM | 0.45 | 0.58 | 0.74 | 0.73 |
| PNUTL1 | 0.22 | 0.86 | 0.77 | 0.51 |
| SMURF1 | 0.27 | 1.12 | 0.79 | 0.47 |
| SMURF2 | 0.06 | 0.62 | 0.44 | 0.47 |
| MYRIP | 0.62 | 0.97 | 0.92 | 0.50 |
| DNCI2 | 0.03 | 1.10 | 0.51 | 0.30 |
| DNCLI2 | 0.11 | 0.98 | 0.44 | 0.46 |
| DYNC1H1 | 0.08 | 0.74 | 1.21 | 0.36 |
| UBE2D1 | 0.39 | 0.95 | 0.56 | 0.85 |
| UBE2D2 | 0.54 | 0.69 | 0.28 | 1.16 |
| UBE2D3 | 0.23 | 0.53 | 0.45 | 0.22 |
| UBE2D4 | 0.11 | 0.96 | 0.61 | 0.50 |
| UBE2E1 | 0.08 | 0.74 | 0.34 | 0.50 |
| UBE2E2 | 1.01 | 0.87 | 0.67 | 0.45 |
| UBE2E3 | 0.34 | 0.62 | 0.59 | 0.40 |
| UBE2I | 0.20 | 1.01 | 0.30 | 0.24 |
| UBE2L3 | 0.21 | 0.82 | 1.11 | 0.43 |
| UBE2N | 0.14 | 0.80 | 0.55 | 0.55 |
| UBE2V1 | 0.67 | 0.83 | 0.28 | 0.44 |
| PSMB6 | 0.09 | 0.11 | 0.23 | 0.29 |
| PSMD4 | 0.10 | 0.88 | 0.61 | 0.36 |
| PSMD5 | 1.29 | 1.03 | 0.62 | 0.75 |
| ANXA1 | 0.33 | 0.91 | 0.53 | 0.70 |
| MRC2 | 0.13 | 0.90 | 0.53 | 0.66 |
| N4BP2 | 0.82 | 1.07 | 0.64 | 0.67 |
| PLSCR1 | 0.29 | 1.01 | 0.37 | 0.65 |
| PLSCR3 | 0.19 | 0.70 | 0.58 | 0.45 |
| WRB | 0.55 | 1.04 | 0.54 | 0.76 |
| XPO1 | 0.01 | 0.17 | 0.58 | 0.05 |

**Table S8:** Functional and pathway analysis of siRNA HFs

| **Term** | **P-Value*^a^*** | **Fold*^b^*** | **FDR*^c^*** |
| --- | --- | --- | --- |
| **GO: together** |  |  |  |
| GO:0031981~nuclear lumen | 4.27E-08 | 1.84 | 5.85E-05 |
| GO:0008380~RNA splicing | 7.17E-08 | 4.63 | 1.23E-04 |
| GO:0006397~mRNA processing | 1.05E-07 | 4.30 | 1.81E-04 |
| GO:0006396~RNA processing | 1.55E-07 | 3.45 | 2.66E-04 |
| GO:0016071~mRNA metabolic process | 8.85E-07 | 3.62 | 0.0015 |
| GO:0030529~ribonucleoprotein complex | 1.21E-06 | 3.40 | 0.0017 |
| GO:0000377~RNA splicing, via transesterification reactions with bulged adenosine as nucleophile | 1.62E-06 | 5.65 | 0.0028 |
| GO:0000398~nuclear mRNA splicing, via spliceosome | 1.62E-06 | 5.65 | 0.0028 |
| GO:0000375~RNA splicing, via transesterification reactions | 1.62E-06 | 5.65 | 0.0028 |
| GO:0043233~organelle lumen | 4.46E-06 | 1.60 | 0.0061 |
| GO:0031974~membrane-enclosed lumen | 9.17E-06 | 1.57 | 0.0126 |
| GO:0005654~nucleoplasm | 9.29E-06 | 1.84 | 0.0127 |
| GO:0070013~intracellular organelle lumen | 1.22E-05 | 1.59 | 0.0167 |
| GO:0003723~RNA binding | 2.58E-05 | 2.60 | 0.0375 |
| **GO: inhibiting only** |  |  |  |
| GO:0006397~mRNA processing | 1.13E-11 | 7.97 | 1.85E-08 |
| GO:0008380~RNA splicing | 1.36E-11 | 8.57 | 2.22E-08 |
| GO:0006396~RNA processing | 6.57E-11 | 5.92 | 1.08E-07 |
| GO:0016071~mRNA metabolic process | 5.25E-10 | 6.39 | 8.61E-07 |
| GO:0000375~RNA splicing, via transesterification reactions | 9.18E-10 | 11.03 | 1.51E-06 |
| GO:0000377~RNA splicing, via transesterification reactions with bulged adenosine as nucleophile | 9.18E-10 | 11.03 | 1.51E-06 |
| GO:0000398~nuclear mRNA splicing, via spliceosome | 9.18E-10 | 11.03 | 1.51E-06 |
| GO:0003723~RNA binding | 2.28E-09 | 4.65 | 3.13E-06 |
| GO:0030529~ribonucleoprotein complex | 3.95E-09 | 5.61 | 5.24E-06 |
| GO:0031981~nuclear lumen | 4.61E-08 | 2.23 | 6.12E-05 |
| GO:0043233~organelle lumen | 8.06E-07 | 1.94 | 0.001 |
| GO:0005681~spliceosome | 1.38E-06 | 10.38 | 0.002 |
| GO:0031974~membrane-enclosed lumen | 1.42E-06 | 1.91 | 0.002 |
| GO:0070013~intracellular organelle lumen | 3.56E-06 | 1.91 | 0.005 |
| GO:0022626~cytosolic ribosome | 9.73E-06 | 12.92 | 0.013 |
| GO:0006414~translational elongation | 1.76E-05 | 11.88 | 0.029 |
| GO:0003735~structural constituent of ribosome | 2.53E-05 | 11.22 | 0.035 |
| GO:0005654~nucleoplasm | 3.19E-05 | 2.14 | 0.042 |
| **GO: enhancing only** |  |  |  |
| NONE |  |  |  |
| **PATHWAYS: together** |  |  |  |
| REACT_71:Gene Expression | 7.02E-13 | 4.1 | 6.79E-10 |
| REACT_6167:Influenza Infection | 4.84E-10 | 6.7 | 4.68E-07 |
| REACT_125:Processing of Capped Intron-Containing Pre-mRNA | 6.46E-08 | 7.0 | 6.25E-05 |
| hsa03040:Spliceosome | 2.37E-07 | 7.3 | 2.76E-04 |
| hsa03020:RNA polymerase | 2.46E-05 | 20.7 | 2.86E-02 |
| **PATHWAYS: inhibiting only** |  |  |  |
| REACT_71:Gene Expression | 4.23E-16 | 5.91 | 4.11E-13 |
| REACT_6167:Influenza Infection | 1.25E-13 | 11.08 | 1.17E-10 |
| REACT_125:Processing of Capped Intron-Containing Pre-mRNA | 1.53E-10 | 11.56 | 1.43E-07 |
| hsa03040:Spliceosome | 2.93E-10 | 13.53 | 3.16E-07 |
| REACT_1762:3' -UTR-mediated translational regulation | 4.53E-06 | 8.52 | 4.21E-03 |
| hsa03010:Ribosome | 5.03E-06 | 14.12 | 5.44E-03 |
| REACT_12472:Regulatory RNA pathways | 1.66E-05 | 24.45 | 1.54E-02 |
| **PATHWAYS: enhancing only** |  |  |  |
| NONE |  |  |  |

*^a^* P-Value, calculated in the Database for Annotation, Visualization and Integrated Discovery (DAVID), using a modified Fisher’s exact test.

*^b^* Fold, fold enrichment of a particular category/pathway for a specific set of genes (1 = neutral). *^c^* FDR, False Discovery Rate.

**Table S9:** Primers and probes for qPCR assays

| **Gene Symbol** | **Forward primer (5' to 3')** | **Reverse primer (5' to 3')** | **UPL Probe #*^a^*** |
| --- | --- | --- | --- |
| MED25 | CTCTGGATGCACAACTGAGAA | GGGGAGACACAATGGAGAAG | 84 |
| MED14 | TGCCTTCATCTCCCACTTCT | AAAGTTTAGTCAACTGCTCCTGAAC | 53 |
| MED23 | TGACATGGTATGGAAGTATAACATTG | CCTTCGTGACTACGCATGG | 13 |
| MED8 | CTTCCATGCATCCCTACCAG | CTTCACCTTGCGCCTTAGAG | 51 |
| UBE2D1 | TATGGGGCCTCCTGATAGC | AAATTTTTGTTGTGAAAGCAATCTT | 39 |
| UBE2D2 | AATGGCAGCATTTGTCTTGA | ATCACACAACAGAGAACAGATGG | 67 |
| UBE2D3 | AATGCTTTCTAACCGAAAGTGC | CATCCCCAACTGGACCTG | 67 |
| UBE2D4 | CGCTAAAGCGGATCCAGA | AACAAGTCATCACCGACAGGT | 51 |
| UBE2E1 | CACCAGCGCCAAGAGAAT | TATCGCCTTTGGGACCAG | 17 |
| UBE2E2 | CAGAAGGAACTTGCAGAAATCA | TCCTTTGGGTCCAGCACTAC | 52 |
| UBE2E3 | GGAGCTAGCTGAAATAACCCTTG | GAACCCGGTGGACCAAGTAT | 63 |
| UBE2I | AGGGAAGTCCCGAGACAAAG | TCCCCGACATGTTCAAAGTC | 39 |
| UBE2L3 | ATGCATTCTGGGGAAGGAG | TTGCGGATTTCTTCAAGCTC | 75 |
| UBE2N | CGCAGGATCATCAAGGAAA | AAATAACGGGCGTTGCTCT | 72 |
| UBE2V1 | AAGCAAGAGCGACGCAAG | ATTGCGAGGGACTTTTACTCC | 83 |
| GTF2A1 | CGATTCTCGAGGTTTTCAGC | GTAGGGGAGAGCGGAGAGAG | 52 |
| GTF3C1 | CATTACCAATGACATCAGAACCA | CTGGGAGGCAACGATGAT | 31 |
| GTF3C2 | CATCCAGAATTGCTGATTCG | ACCCCGCAGGTATCCATC | 46 |
| NR1H2 | CGCTGTTGCTTGGAGAGG | GAGGACATGGTGGGGTCA | 80 |
| NR3C2 | TTTTCTTCAAAAGAGCAGTGGA | GGACAATTCTTTCGTCGAATCT | 11 |
| NR4A2 | TGAAGAGAGACGCGGAGAAC | AAAGCAATGGGGAGTCCAG | 63 |
| HOXA10 | CCTACACGAAGCACCAGACA | CGCTCTCGAGTAAGGTACATATTG | 78 |
| HOXA5 | GCGCAAGCTGCACATAAG | CGGTTGAAGTGGAACTCCTT | 1 |
| HOXC5 | CCCAGCAAGTGGTCCTAGAG | AGGAGGGCACAGAAATTCG | 56 |
| CBX4 | GCTGATCGCCTTCCAGAAC | CCTCTCTTCCGATATCCCATC | 51 |
| CBX5 | GCGGACAGCTGACAGTTCTT | TTCCCAAGTATTGTGCTCCTC | 63 |
| PSMB6 | GGCGGCTACCTTACTAGCTG | AAACTGCACGGCCATGATA | 48 |
| PSMD4 | TTGGAGGAGTTGTTGTTAGGC | CGCATATACTCACTGTTGTCCAC | 69 |
| PSMD5 | GCTGACTGGTGAGGATGTGTT | TGCCAGTGATGTCACCATTT | 21 |
| TOP2A | CAACATGCCAATTGAGTGAAA | GGACTTGGGCCTTAAACTTCA | 76 |
| TOP3B | CCCTTCCGAGACATGAAGAA | CAGCATGCTCAGCGAGTG | 51 |
| TOPBP1 | TGCAGGACTGCTGGACACT | GTATGGAACTACTTCCCCATTCA | 4 |
| DNCI2 | GCATGGGGAGATTGGATTTA | ATGGGTCCATCTCACACGAT | 15 |
| DNCLI2 | CATGATGAGGACCGAGATGA | TCAGCAGGCCTTTGTGGTA | 58 |
| DYNC1H1 | AGTTGGTGGAATGTGGGTTG | TGATTGATCTGGGTGATCTGA | 78 |
| EPIM | CGGAGTACAATGAGGCACAG | CGTCGTCTGTGGTGGTTCT | 42 |
| RAB10 | TTTCGGATGATGCCTTCAAT | TCTTTCCTTGTAATTCAACTGTTTTG | 31 |
| RAB13 | CCTCTTGCTGGGGAACAA | TTTCGAAAAATCGGATTCCA | 68 |
| RAB26 | TCTACGACGTCGCCTTCAA | TTGAATCGCACCAGCAGAC | 60 |
| RAB6A | CCGTAGCCTCATTCCCAGT | TCCACTTTGTAGTTTGCTGGAA | 84 |
| SCAMP3 | TTGACGTCTACAACCCTTTTGA | GGGCTGGAGGCTCATAGG | 77 |
| VAMP8 | CCTGGGCTGCTCTGAGAC | TCCGCACACGATCATTTC | 69 |
| ITGA2 | TCGTGCACAGTTTTGAAGATG | TGGAACACTTCCTGTTGTTACC | 7 |
| ITGA6 | TTTGAAGATGGGCCTTATGAA | CCCTGAGTCCAAAGAAAAACC | 22 |
| ITGAV | GCCGTGGATTTCTTCGTG | GAGGACCTGCCCTCCTTC | 64 |
| ITGB2 | CAGCAATGTGGTCCAACTCA | GAGGGCGTTGTGATCCAG | 25 |
| ITGB3 | CGCTAAATTTGAGGAAGAACG | GAAGGTAGACGTGGCCTCTTT | 76 |
| PVRL1 | TGCCTGATAGAGGCATTTCC | GGCAGTGCAGGATAAAGGAG | 63 |
| PVRL2 | GAGGACGAGGGCAACTACAC | GGCCTCAGCTTGGTTCTTG | 60 |
| CSNK1G1 | CTGTGACCGACATTTACTTTGA | TGCACGTATTCCATTCGAGA | 42 |
| CSNK2A2 | CCATGGAGCACCCATACTTC | CACAGCATTGTCTGCACAAG | 68 |
| GAB1 | ATCCACACCACCACGTAAGC | CAACAACATAATCCACTCTCTCATC | 27 |
| GAB2 | CGAAGAGAACTATGTCCCTATGC | AGGGGCAGGACTGTTCGT | 48 |
| PI4KA | TTTGGACCGCCATGTTCT | TCCGGGTGTCCTGATTATCT | 20 |
| PLCG1 | CGGAATCGTGAGGATCGTA | GGGACCCGGTAGTTGACC | 56 |
| PTPRJ | GTCCTGTCCTAGGTGACATCG | GGAAGTCAGAAACTGGAACAGG | 69 |
| SRC | GCCAAATTCCCCATCAAGT | GATGGTGAAGCGGCCATA | 50 |
| AHCYL1 | TGGTGTGTGGCTATGGTGAG | GGGGTCGATTTCGGTAATGT | 66 |
| CTBP1 | CGAGTCGGAACCCTTCAG | CAGATGAGGTTGGGTGCAT | 81 |
| CTBP2 | GCTCAATGGTGCCACATACA | TCCATGGCTGCAGGAAGT | 1 |
| SUMO1 | AAGCCACCGTCATCATGTCT | TTATCCCCCAAGTCCTCAGTT | 79 |
| IRF1 | GGCACATCCCAGTGGAAG | CCCTTCCTCATCCTCATCTGT | 56 |
| IRF3 | CTTGGAAGCACGGCCTAC | CGGGAACATATGCACCAGT | 18 |
| IRF7 | GGTGACAAGGGGGACCTC | GACGCTGTCAGCAGATGGT | 1 |
| IL2 | CAGTGTCTAGAAGAAGAACTCAAACCT | GGTCTTAAGTGAAAGTTTTTGCTTTG | 69 |
| IL4 | CTTTGTCAGCATTGCATCGT | GATTTGCAGTGACAATGTGAG | 2 |
| IL5 | TTCCTGTTCCTGTACATAAAAATCAC | CAGTACCCCCTTGCACAGTT | 47 |
| IL6 | GATGAGTACAAAAGTCCTGATCCA | CTGCAGCCACTGGTTCTGT | 40 |
| IL8 | AGACAGCAGAGCACACAAGC | ATGGTTCCTTCCGGTGGT | 72 |
| IL10 | CATAAATTAGAGGTCTCCAAAATCG | AAGGGGCTGGGTCAGCTAT | 45 |
| IFN-β | CTTTGCTATTTTCAGACAAGATTC | GCCAGGAGGTTCTCAACAAT | 25 |
| IFN-λ_1_ | CTGTCCCCACTTCCAAGC | TCAGCTTGAGTGACTCTTCCAA | 48 |
| IFN-λ_2/3_ | GCCCAGTTCAAGTCCCTGT | TCCTTCAGCAGAAGCGACTC | 17 |
| Med23-R611Q | GATGTTTAGCTACCAGATGCATCATATTCAGCCTCATTAC | GAATATGATGCATCTGGTAGCTAAACATCTCAAGGAGTG | - |
| R611Q-Seq | CTATGAACCTCCTGGATTCAC |  | - |

**References**

1. Brass AL, Huang IC, Benita Y, John SP, Krishnan MN, et al. (2009) The IFITM proteins mediate cellular resistance to influenza A H1N1 virus, West Nile virus, and dengue virus. Cell 139: 1243-1254.

2. Karlas A, Machuy N, Shin Y, Pleissner KP, Artarini A, et al. (2010) Genome-wide RNAi screen identifies human host factors crucial for influenza virus replication. Nature 463: 818-822.

3. Konig R, Zhou Y, Elleder D, Diamond TL, Bonamy GM, et al. (2008) Global analysis of host-pathogen interactions that regulate early-stage HIV-1 replication. Cell 135: 49-60.

4. Krishnan MN, Ng A, Sukumaran B, Gilfoy FD, Uchil PD, et al. (2008) RNA interference screen for human genes associated with West Nile virus infection. Nature 455: 242-245.

5. Konig R, Stertz S, Zhou Y, Inoue A, Hoffmann HH, et al. (2010) Human host factors required for influenza virus replication. Nature 463: 813-817.

6. Brass AL, Dykxhoorn DM, Benita Y, Yan N, Engelman A, et al. (2008) Identification of host proteins required for HIV infection through a functional genomic screen. Science 319: 921-926.

7. Zhou H, Xu M, Huang Q, Gates AT, Zhang XD, et al. (2008) Genome-scale RNAi screen for host factors required for HIV replication. Cell Host Microbe 4: 495-504.

8. Tai AW, Benita Y, Peng LF, Kim SS, Sakamoto N, et al. (2009) A functional genomic screen identifies cellular cofactors of hepatitis C virus replication. Cell Host Microbe 5: 298-307.

9. Li Q, Brass AL, Ng A, Hu Z, Xavier RJ, et al. (2009) A genome-wide genetic screen for host factors required for hepatitis C virus propagation. Proc Natl Acad Sci U S A 106: 16410-16415.

10. Sessions OM, Barrows NJ, Souza-Neto JA, Robinson TJ, Hershey CL, et al. (2009) Discovery of insect and human dengue virus host factors. Nature 458: 1047-1050.
