# Supplementary figures and images for: A Systematic Analysis of Host Factors Reveals a Med23-Interferon-λ Regulatory Axis against Herpes Simplex Virus Type 1 Replication
Source: PLoS Pathog. 2013 Aug 8;9(8):e1003514. doi: 10.1371/journal.ppat.1003514 (PMC3738494; doi:10.1371/journal.ppat.1003514)

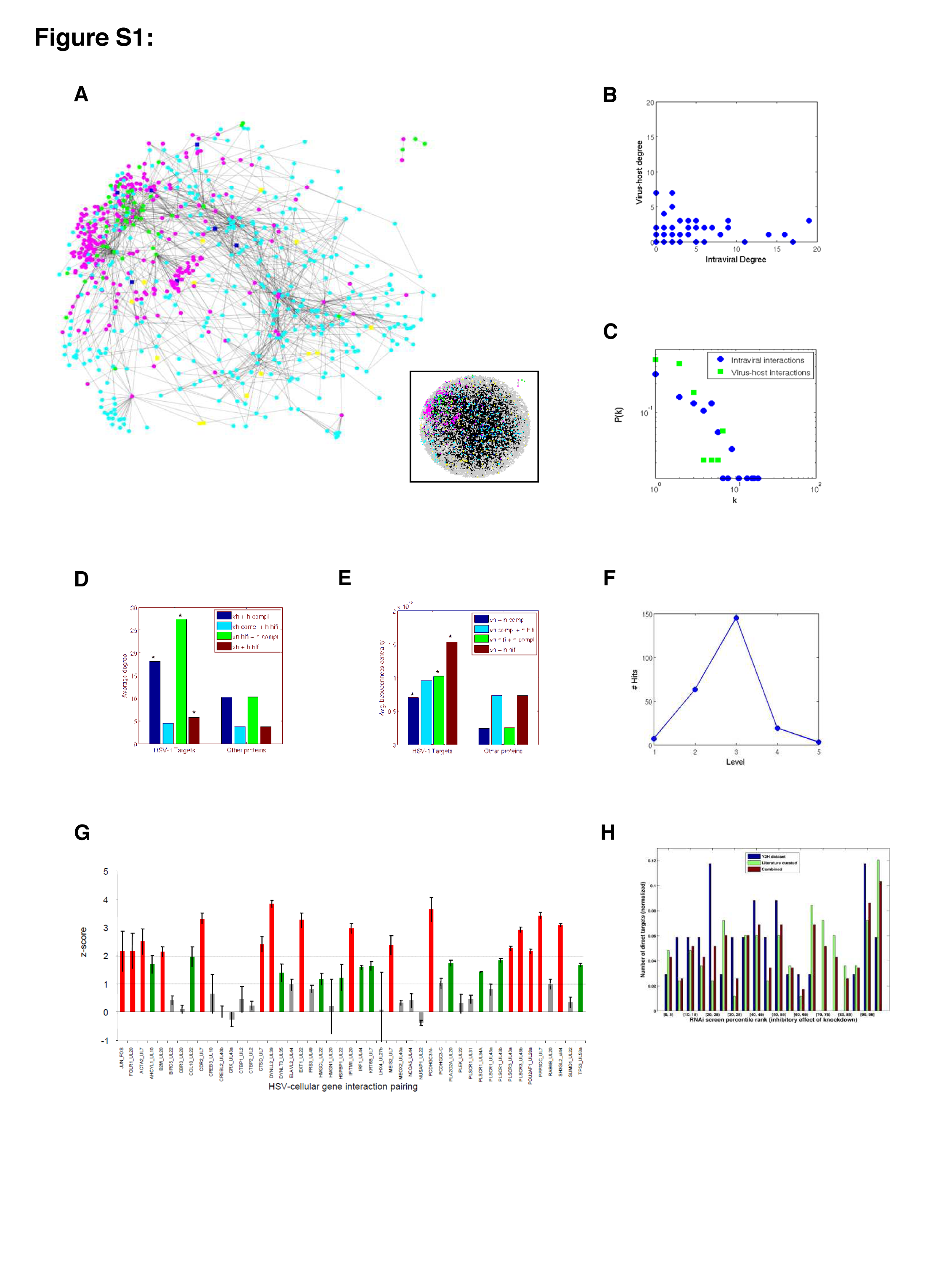

Supplement: Figure S1 — Pathogen-host interactome analysis of RNAi and Y2H HFs. (a) Combined HSV-1-human interactomes with intraviral, virus-host and high-confidence host-host interactions. Green, viral proteins; Pink, direct (level 1) interactors (Y2H screen); Blue, level 1 interactors (Y2H/RNAi screens); Cyan, host protein-protein (level 2) interactors (Y2H screen); Yellow, Level 2 interactors (Y2H/RNAi screens). Due to the large size of the human network, and in order to discern viral proteins, only the first two levels were plotted (inset: all levels). Combined HSV-1-human interactome. Inset, all levels. (b) Intraviral and virus-host degree (# interactions) distribution of HSV-1 proteins. (c) Correlation between intraviral and virus-host degrees in HSV-1. (d) Degree comparison of cellular interactors versus all proteins in human networks, where the degree indicates the number of interactions a particular protein has. (e) Betweenness centrality comparison of cellular interactors versus all proteins in human networks, where betweenness indicates the number of shortest paths between a protein pair, passing through the protein of interest. Statistically significant differences between viral targets and remaining proteins are denoted by *. (f) Distribution of HFs in the virus-host interactomes. Level 1 proteins are direct interactors with HSV-1 proteins, level 2 their interactors and so on. (g) Validation of HSV-1-host Y2H interactors. A subset of protein interactions identified in the HSV-1-host Y2H screen were validated using the LUMIER pull-down assay in a mammalian cell system. Strength of interaction was determined by Z-score, where a score 1 to 2 represents a weak interaction and score >2 represents a strong interaction. (h) Distribution of direct HSV-1 targets in the RNAi screen. The proteins directly targeted by HSV-1 were taken from the Y2H data set and from the literature curation. An enrichment of literature-derived targets could be observed in the top 5% most inhibiting knockdow [file ppat.1003514.s003.tif]

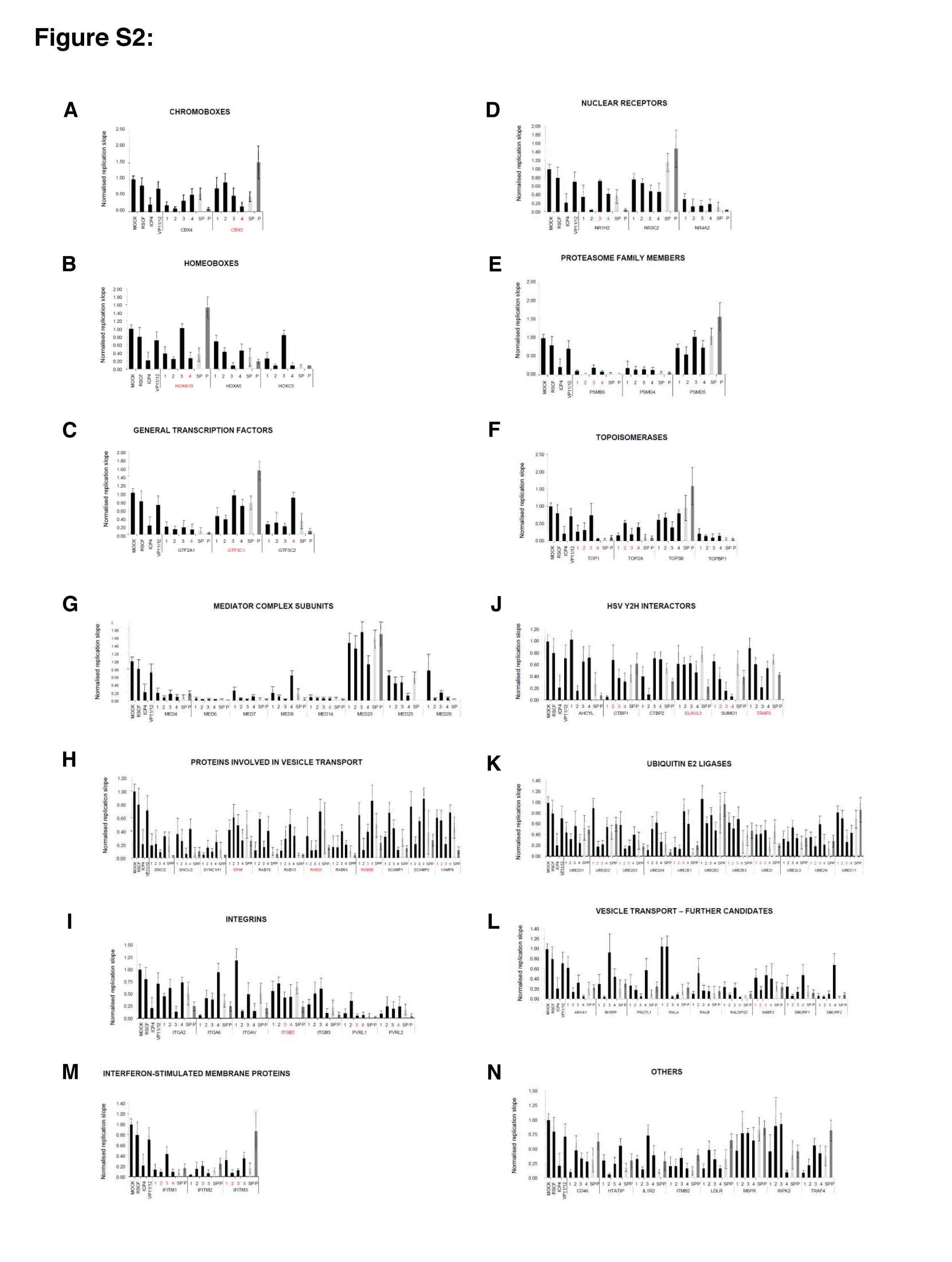

Supplement: Figure S2 — Validation of HF identified by RNAi. HFs identified in the HSV-1 perturbation screen were validated with deconvoluted siRNAs and qPCR: (a) chromoboxes, (b) homeoboxes, (c) general transcription factors, (d) nuclear receptors, (e) proteasome family members, (f) topoisomerases, (g) mediator complex subunits, (h) proteins involved in vesicle transport, (i) integrins, (j) Y2H interactors, (k) ubiquitin E2 ligases, (l) vesicle transport – further candidates, (m) interferon-stimulated membrane proteins and (n) others. HSV replication is presented as normalized replication slope, and is the mean of six individual assay points. Error bars represent standard deviation of the six data points. Deconvoluted siRNAs which had a sequence different to that in the original screen are highlighted in red. Genes for which the primary screen phenotype was not confirmed in the deconvolution assay are shown in bold text. (TIF) [file ppat.1003514.s004.tif]

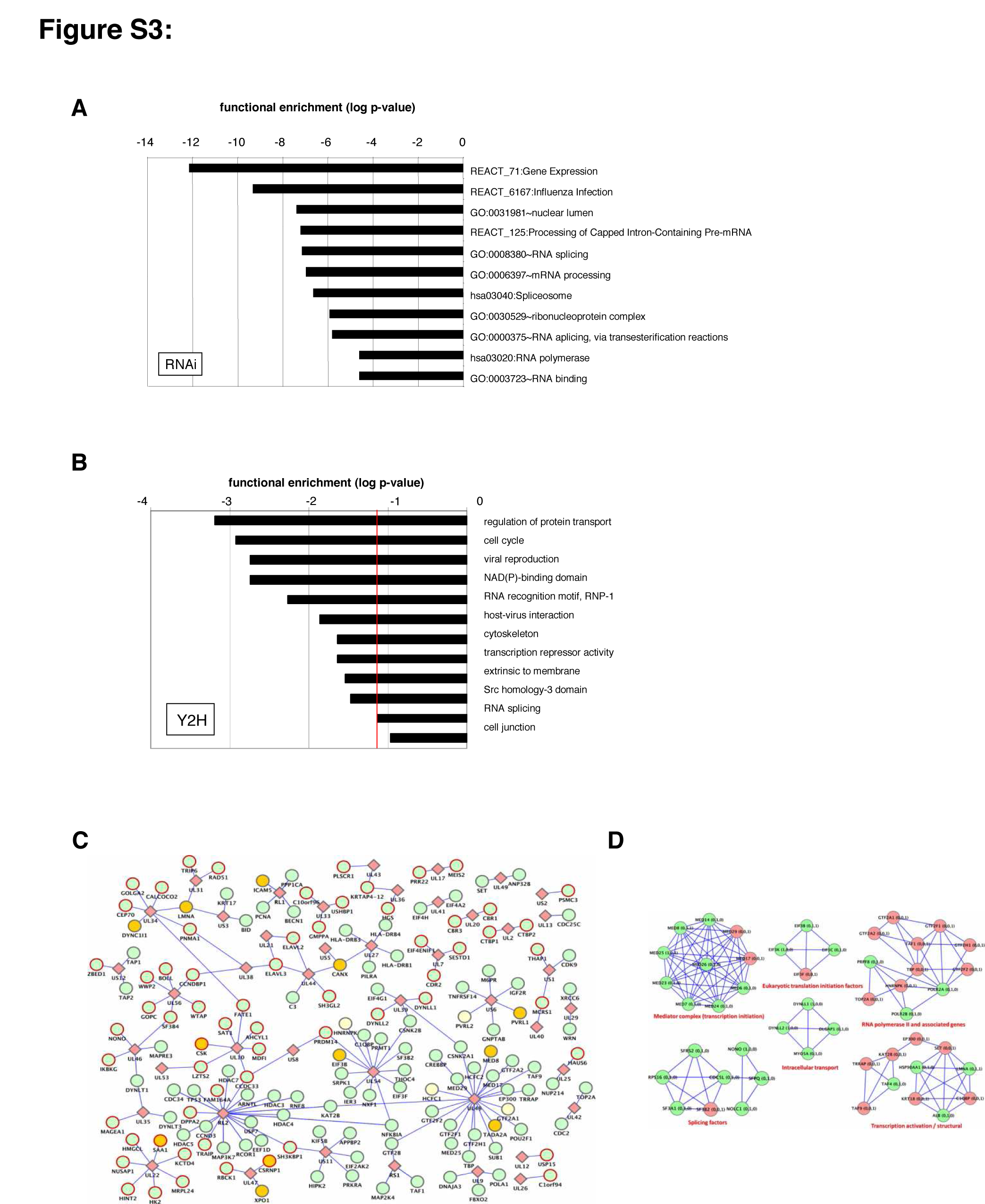

Supplement: Figure S3 — HSV-1 HFs are involved in diverse cellular pathways and at multiple stages of the HSV life cycle. Enrichment of protein functions among the HSV-1 HFs. The enrichment for gene ontology (GO) terms and KEGG, BIOCARTA or REACTOME pathway annotations among the HSV-1 HFs identified by (a) RNAi and (b) Y2H assay was performed using DAVID bioinformatics software. (c) Direct interactions between human and HSV-1 proteins. The protein-protein interactions (PPIs) depicted are from the high confidence Y2H data set and from literature curation. Circles and diamonds correspond to human and HSV-1 proteins, respectively. Human proteins detected in the Y2H screen are drawn with red borders. Human genes that showed the strongest effects in the RNAi screen are colored yellow (extreme 10%) and orange (extreme 5%). (d) Highly interconnected regions in the human interaction network composed of HFs. Highly interconnected regions in the human interaction network composed of HFs. We assembled a human interaction network using data from the major PPI databases. A subnetwork consisting of HFs was then defined by limiting the network to the HFs detected in the Y2H screen, the RNAi (extreme 5%) screen, and the literature curation. Highly interconnected regions in the subnetwork were sought out using the MCODE algorithm. The top six scoring regions are shown. Proteins displayed in red correspond to HFs that are known only from the literature, and those in green are those that were detected in either of the screens performed in this study. The three boolean values beside each gene symbol represent whether the HF is present in the Y2H screen, the RNAi screen (extreme 5%), or the literature curated set, in that order. Dominant functional categories could be observed in each of the regions, including transcription (e.g., Mediator complex, RNA polymerase II and associated genes), translation initiation, splicing, and intracellular transport. (TIF) [file ppat.1003514.s005.tif]

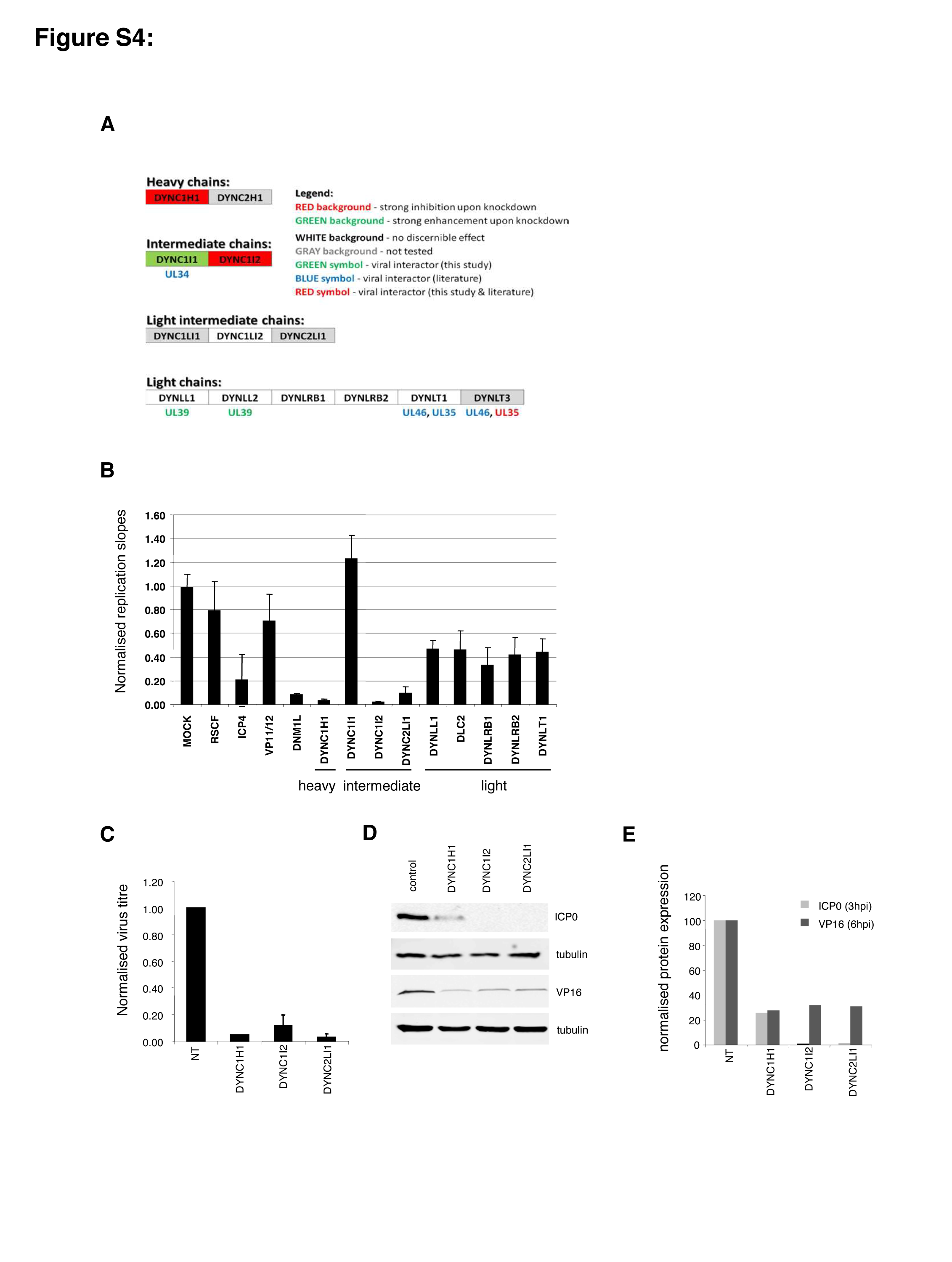

Supplement: Figure S4 — HFs involved in viral entry and capsid transport. (a) Diagrammatic summary of the role of dynein chains in HSV-1 infection. (b) Microtubule transport is required for HSV-1 infection. The role of dynein microtubule transport components in HSV-1 replication was analysed by comparing the replication slope of HSV-1-eGFP (C12)-infected cells depleted for a range of dynein chains from the primary siRNA perturbation screen. Error bars represent the mean of three independent experiments done in duplicate. (c) Depletion of dyneins inhibits virus particle release. The effect of dynein chain depletion on HSV-1 particle release was determined by quantifying virus titer in supernatants of Hela cells depleted of DYNC1H1 (heavy chain), DYNC1I2 (intermediate chain) and DYNC2LI1 (light intermediate chains) in a high multiplicity (MOI 5; HSV-1 KOS) growth assay. Titers were compared to control transfected cells (NT, non-targeting siRNA). (d) Depletion of dynein chains prevents immediate-early gene expression. Immediate-early (ICP0) and late (VP16) viral protein expression in cells depleted of DYNC1H1, DYNC1I2 or DYNC2LI1 was analysed and quantified by Western blot. Levels were compared to control transfected cells (NT, non-targeting siRNA). (e) Quantification of ICP0 and VP16 protein expression. Protein levels of ICP0 and VP16 in cells depleted of DYNC1H1, DYNC1I2 or DYNC2LI1 were quantified with an Odyssey Imager and normalized to protein levels in control transfected cells (Non-targeting siRNA). (TIF) [file ppat.1003514.s006.tif]

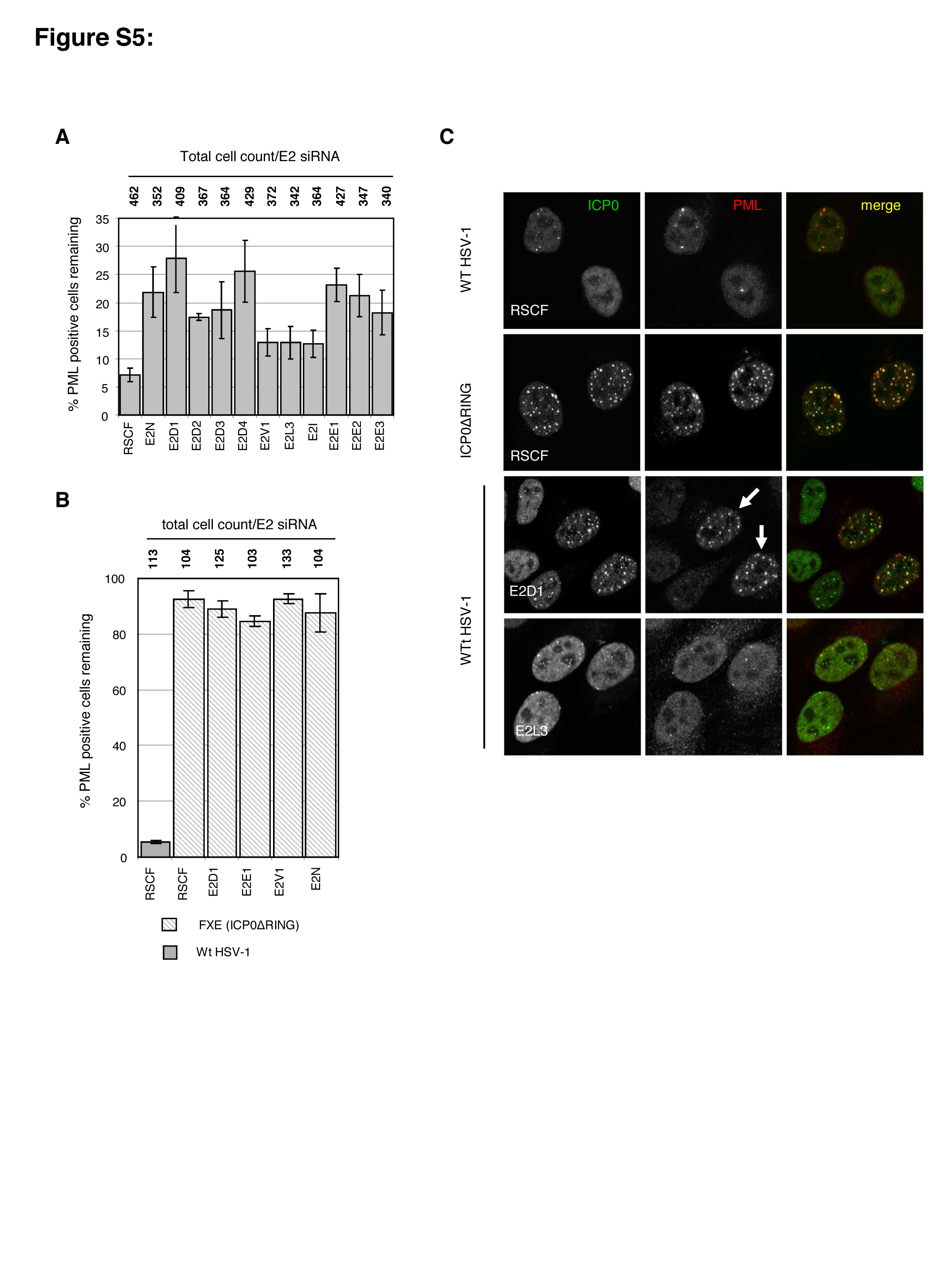

Supplement: Figure S5 — E2 ubiquitin conjugating enzymes in HSV-1 immune evasion. (a) Depletion of E2 ubiquitin ligases inhibits PML degradation following HSV-1 infection. Hela cells mounted on coverslips were depleted for a range of E2 ubiquitin ligases for 24 h before infecting with HSV-1 17+. Cells were fixed and stained for ICP0 (green) and PML (red), analysed by confocal microscopy and PML-positive cells were counted (5 fields of view per coverslip) and expressed as a mean percentage of PML-positive cells remaining (3 independent experiments). Error bars represent the standard deviation over 3 independent experiments. (b) Inhibition of PML degradation by E2s is ICP0-dependent. Hela cells were seeded on coverslips, transfected as above and infected with an ICP0 RING-finger deletion mutant (FXE). Remaining PML-positive cells were quantified as above. (c) Immunofluorescence staining for PML bodies in cells transfected with control siRNA not incorporated into the RISC complex (RSCF) or cells depleted of the E2 ubiquitin conjugating enzymes E2D1 or E2L3. The arrows highlight cells that have wt PML levels remaining in them whilst containing wt ICP0. (TIF) [file ppat.1003514.s007.tif]

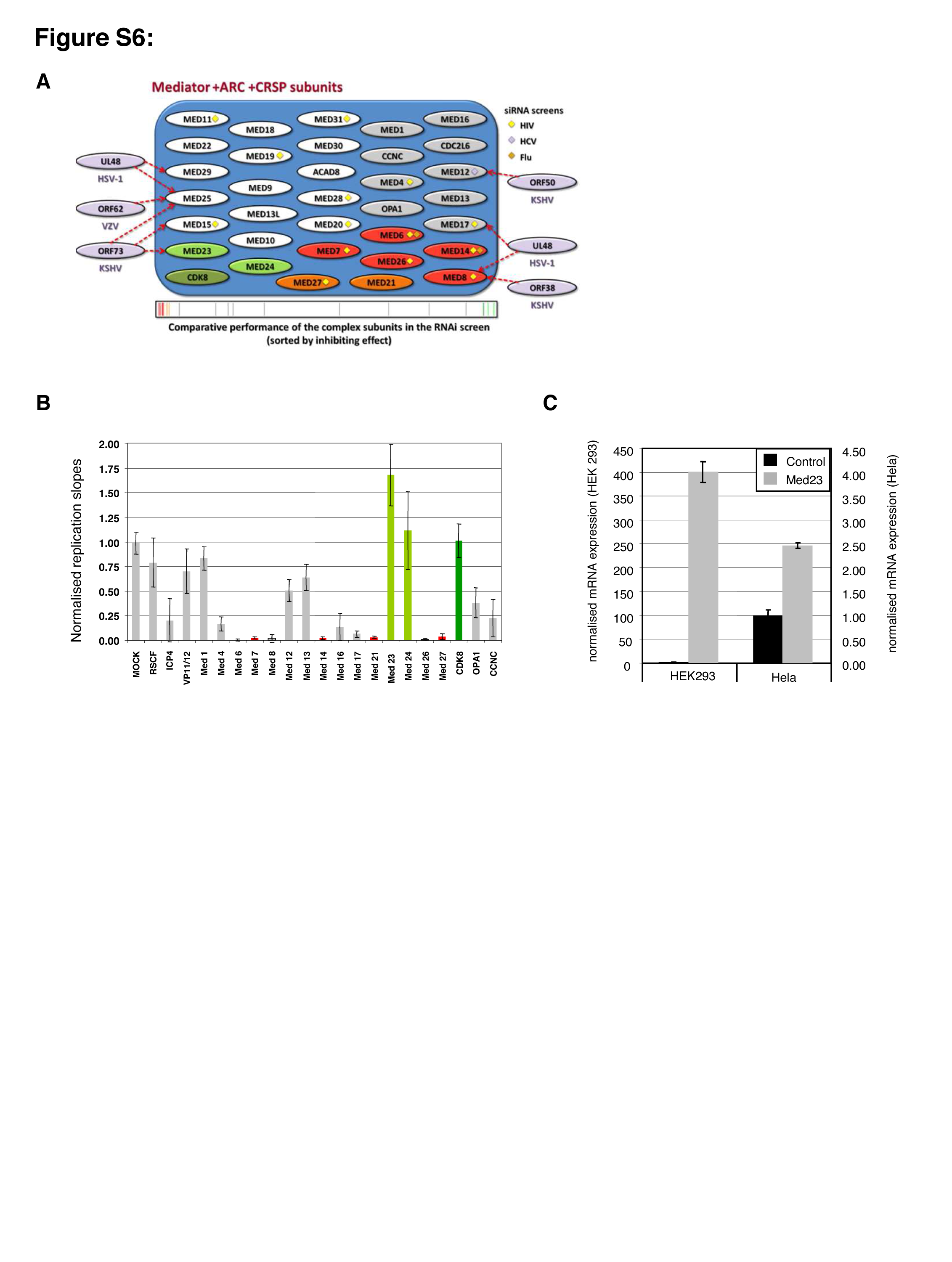

Supplement: Figure S6 — Med23 is an anti-viral component of the largely pro-viral multi-protein Mediator complex. (a) Diagrammatic summary of the role of Mediator complex subunits in virus replication. Subunits are coloured according to whether HSV-1 replication was unchanged (grey), inhibited (top 5%, red; top 10%, orange) or enhanced upon gene knockdown (top 5%, light green; top 10%, dark green). Subunits not included are white; herpesvirus proteins reported to target Mediator subunits are violet; Mediator subunits detected in other viral RNAi screens are highlighted by coloured diamonds. (b) Mediator complex subunits influence HSV-1 replication. Individual subunits of the Mediator complex and associated proteins were depleted by siRNA knockdown and infected with HSV-1 C12 (MOI 0.5). Replication was monitored over multiple rounds and the slope of replication over the linear phase was calculated and normalized to controls (mock-transfected cells). Grey, no significant effect; red, strongly pro-viral; green, strongly anti-viral. Error bars represent the mean of six replicates. (c) Stable or transient overexpression of Med23. Med23 was stably (HEK) or transiently (Hela) overexpressed and Med23 mRNA levels quantified by RT-PCR. Expression was normalized to HPRT and calibrated to parental cells. Error bars represent the standard deviation of technical replicates. (TIF) [file ppat.1003514.s008.tif]

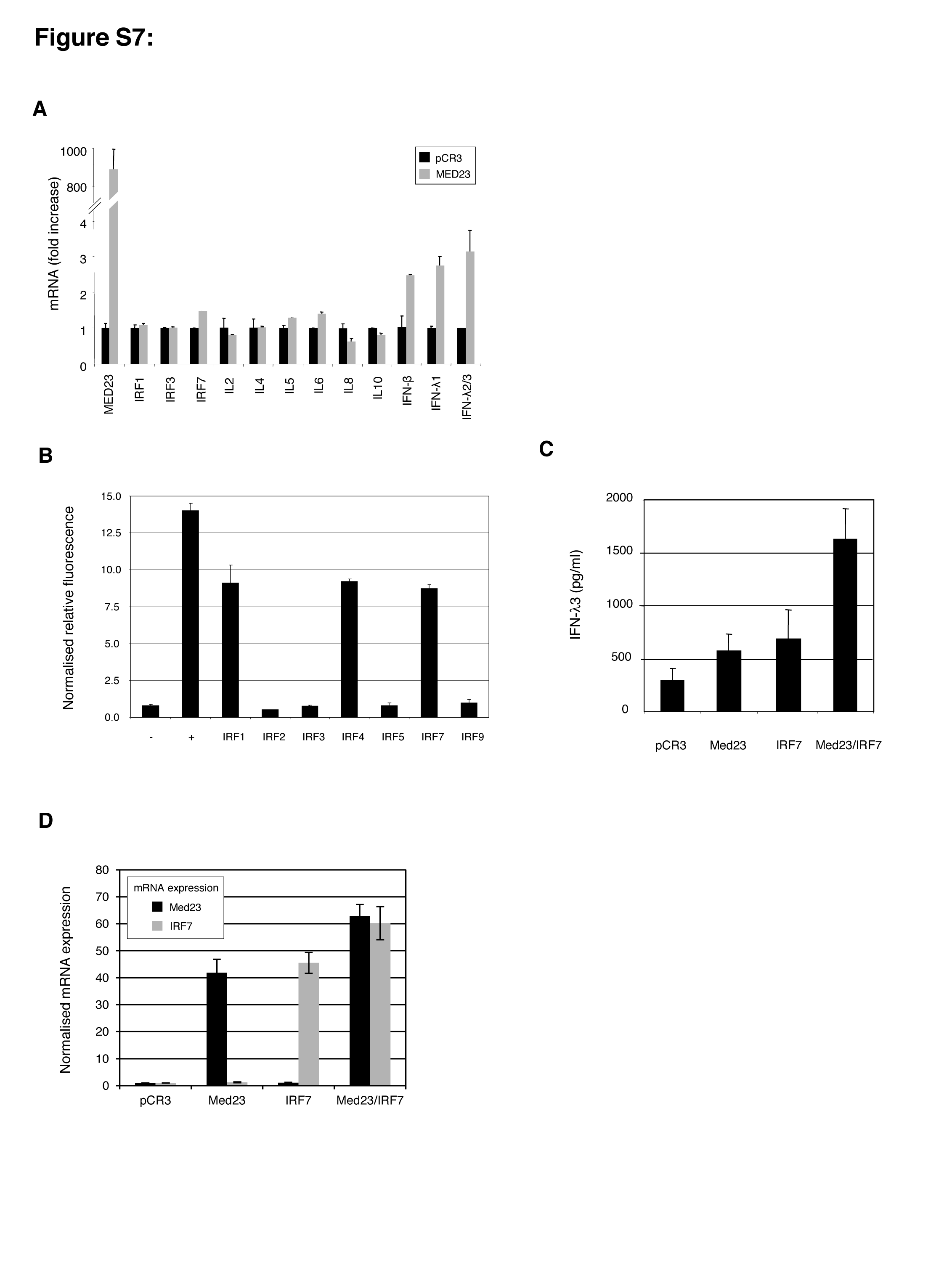

Supplement: Figure S7 — Med23 inhibits HSV-1 by directly interacting with IRF7 to induce a Type III, IFN-λ interferon response. (a) Med23 specifically induces interferons. A549 cells were transfected with pCR3 or Med23 and induction of a panel of cytokines and interferons determined by qRT-PCR. Expression was normalized to HPRT and calibrated to pCR3-transfected cells. Error bars represent the standard deviation of technical replicates. (b) Med23 directly interacts with IRF7. Haploid yeast expressing Med23-bait or IRF-prey constructs were mated in nutrition-deficient Media containing 4-MuX. Interactions were detected by measurement of fluorescence released by α-galactosidase cleavage of 4-MuX upon protein interaction. Relative fluorescence (RFU) was normalized to the negative control (−, empty bait mated with empty prey constructs). Error bars represent the standard deviation of technical quadruplicates. +, known interactors Myc and Max. (c) Med23 and IRF7 synergistically induce IFN-λ secretion. A549 cells were transfected with pCR3, Med23, IRF7 or Med23 with IRF7 and supernatant was harvested after 96 h to measure IFN-λ3 levels (pg/ml) by ELISA. Error bars represent the standard deviation of biological replicates, and the chart is representative of multiple experiments. (d) Quantification of Med23 and IRF7 following overexpression. A549 cells were transfected with pCR3, Med23, IRF7 or Med23 with IRF7 and mRNA expression levels of Med23 or IRF7 quantified by qRT-PCR 96 h post-transfection. Error bars represent the standard deviation of biological replicates, and the chart is representative of multiple experiments. (TIF) [file ppat.1003514.s009.tif]
